# Genetic variants associated with osteosarcoma risk: a systematic review and meta-analysis

**DOI:** 10.1038/s41598-024-53802-w

**Published:** 2024-02-15

**Authors:** Omneya Hassanain, Mahmoud Alaa, Mohamed K. Khalifa, Nehal Kamal, Aseel Albagoury, Ahmed M. El Ghoneimy

**Affiliations:** 1 Epidemiology and Biostatistics Unit, Clinical Research, Children’s Cancer Hospital Egypt-57357 (CCHE-57357), 1 Seket el Emam, el Sayeda Zeinab, Cairo, 11441 Egypt; 2 Basic Research, Children’s Cancer Hospital Egypt-57357 (CCHE-57357), Cairo, Egypt; 3 Molecular Pathology Laboratory, Children’s Cancer Hospital Egypt-57357 (CCHE-57357), Cairo, Egypt; 4 Department of Orthopedic Oncology, Children’s Cancer Hospital-57357 (CCHE-57357), Cairo, Egypt; 5 Department of Orthopedics, Faculty of Medicine, Cairo University, Cairo, Egypt

**Keywords:** Osteosarcoma, Bone tumors, Single nucleotide polymorphisms (SNPs), Cancer, Bone cancer, Cancer epidemiology, Cancer genomics

## Abstract

Osteosarcoma (OS) is the most common type of primary bone malignancy. Common genetic variants including single nucleotide polymorphisms (SNPs) have been associated with osteosarcoma risk, however, the results of published studies are inconsistent. The aim of this study was to systematically review genetic association studies to identify SNPs associated with osteosarcoma risk and the effect of race on these associations. We searched the Medline, Embase, Scopus from inception to the end of 2019. Seventy-five articles were eligible for inclusion. These studies investigated the association of 190 SNPs across 79 genes with osteosarcoma, 18 SNPs were associated with the risk of osteosarcoma in the main analysis or in subgroup analysis. Subgroup analysis displayed conflicting effects between Asians and Caucasians. Our review comprehensively summarized the results of published studies investigating the association of genetic variants with osteosarcoma susceptibility, however, their potential value should be confirmed in larger cohorts in different ethnicities.

## Introduction

Osteosarcoma (OS) is the most common primary bone malignancy, predominantly occurring during adolescence. Representing 2.4% of all childhood cancer, it is most commonly diagnosed between the ages of 10 and 30 years^1^. OS exhibits a slight male predominance and is more frequent in African Americans, Asian/Pacific Islanders, and Hispanics compared to the White population^2^. The etiology of Osteosarcoma remains elusive, but several epidemiological risk factors have been associated with an increased disease risk. Notably, a higher risk of osteosarcoma is documented in hereditary retinoblastoma, Li-Fraumeni syndrome, Rothmund-Thompson syndrome, and Bloom and Werner syndrome^3^. Other predisposing conditions, such as Paget’s disease of the bone, fibrous dysplasia, and exposure to irradiation, are also linked with an increased risk, particularly in older populations^4–6^. Interestingly, osteosarcoma is more frequently reported in individuals with tall stature relative to the normal population^7^.

Osteosarcoma encompasses various subtypes, most of which are high-grade and exhibit aggressive biological behavior^8^. Despite advances in OS treatment, outcomes remain suboptimal^9^. Significant variations in the response and toxicity of chemotherapy drugs are observed due to genetic variation^10^. Identifying the genetic basis for these variations could significantly alter the progress in treatment of such rare cancer. Single-nucleotide polymorphisms (SNPs), which are changes in a single base in exonic or intronic regions, have been implicated in altering gene expression or being in linkage disequilibrium with causal loci associated with cancer prognosis and/or risk^11^. Multiple genes, such as ABCB1, GSTP2, VEGD, GRM4, and key enzymes of DNA repair, have been identified as predictors of OS prognosis^12–14^. Systematic reviews have summarized evidence on SNPs associated with OS outcomes^15^.

Studies have identified common SNPs in genes important for growth and tumor suppression, hypothesized to modify osteosarcoma susceptibility, such as CTLA-4, ERCC2, and TP53^16–18^. However, the results of these studies are often inconsistent, limited by small sample sizes, and thus inconclusive^19^. Conflicting risk associations according to race, noted in gastric cancer and other health conditions, highlight the potential impact of race and the caution needed when combining results from different races^20–22^.

Some systematic reviews have assessed the evidence for an association between SNPs and individual genes with OS, but few have evaluated and summarized all SNPs associated with OS^23–26^. This underscores the need for a comprehensive synthesis and analysis.

systematic review addresses a significant gap in osteosarcoma research, focusing on the This comprehensive evaluation of single nucleotide polymorphisms (SNPs) and their association with OS risk. While previous meta-analyses have explored links between specific genes with OS^23,27–29^, a holistic summary encompassing all SNPs related to OS, particularly considering racial disparities, has been largely unexplored. The primary objective of this systematic review was to identify and analyze single nucleotide polymorphisms (SNPs) associated with osteosarcoma. This endeavor seeks to bridge a substantial gap in the current understanding of osteosarcoma genetics. Additionally, the review places a strong emphasis on examining the impact of racial disparities on genetic susceptibility to osteosarcoma, thereby contributing to a more nuanced understanding of the disease's genetic landscape.

## Methods

A review protocol was drafted and registered in the International Prospective Register of Systematic Reviews (PROSPERO); with registration number CRD42021236787. Reporting was performed in accordance with the Preferred Reporting Items for Systematic Reviews and Meta-analysis (PRISMA) statement^30^.

### Search strategy

We conducted a comprehensive search of MEDLINE, Ovid/Embase, Scopus, and the Cochrane databases for genetic association studies on Osteosarcoma from inception until the end of 2019. Keywords, including MeSH (Medical Subject Heading) terms and free-text words, were utilized in both titles and abstracts. The search terms included: “Neoplasms, Bone Tissue," “osteosarcoma,” “cancer(s),” “SNP,” “single nucleotide polymorphism,” “Disease Susceptibility,” and “Genetic Association Studies." Details of the search terms used are provided in the Supplementary material. To ensure comprehensive coverage, the search strategy was intentionally broad.

Additionally, the Scopus citation database was utilized to identify publications citing relevant previous works. A manual search was conducted in the reference lists of eligible papers and previously published systematic reviews. This strategy aimed to include all relevant published original peer-reviewed articles, imposing no restrictions on publication status.

Initial screening involved excluding irrelevant studies by scanning titles and abstracts. Subsequently, potentially eligible studies were retrieved for full-text review. The search was restricted to studies published in English. The eligibility criteria for inclusion were: (i) studies assessing the association between a genetic variant and osteosarcoma susceptibility, (ii) case–control studies in humans with osteosarcoma patients as cases and healthy subjects or patients with non-malignant diseases as controls, (iii) no restrictions based on race, geographical location, or disease stage, (iv) no age limit, and (v) availability of sufficient genotype data.

The primary exclusion criteria were: (i) reviews, conference reports, communications, or letters without primary data, (ii) data from cell lines and non-human experiments, (iii) studies not reporting genotype frequencies, and (iv) articles not in English. Any discrepancies encountered during the review process were resolved through group discussions.

### Data extraction

Three investigators (AG, MA, and NK) independently conducted data extraction from the eligible studies using a standardized form. The extracted information included: the first author's surname, publication journal and year, country of origin, participant sex and age, sample size, identified genetic mutations, frequencies of genotypes or alleles, and the genotyping methods used. For clarity and ease of cross-referencing, genetic polymorphisms were recorded using their most commonly accepted notations. Studies with vague, insufficient, or missing data that could not be resolved or supplemented through other measures were subsequently excluded.

### Qualitative evaluation

The assessment of the quality of the retrieved studies was independently conducted by two reviewers employing the Quality of Genetic Studies (Q-Genie) tool^31^. In cases of disagreement between the reviewers, a third reviewer was consulted to resolve any discordance.

### Statistical analysis

Pooled odds ratios (ORs) and their corresponding 95% confidence intervals (CIs) were determined using the random-effects model, specifically the Sidik-Jonkman method, with a *P*-value < 0.05 set as the threshold for statistical significance. In the absence of conclusive evidence regarding the most appropriate genetic model for identifying associations of SNPs with osteosarcoma, pooled ORs were calculated under five genetic models: homozygous, heterozygous, dominant, recessive, and allele. While adjustments for multiple tests were considered, we adhered to the Cochrane recommendation of not adjusting for multiple testing^32^. Hardy–Weinberg equilibrium (HWE) was tested in control groups using the chi-square test; a *P*-value < 0.05 indicated deviations from HWE. Pooled ORs were recalculated excluding studies deviating from HWE, and pooled estimates were reported both with and without these studies.

The association between polymorphisms and osteosarcoma was analyzed separately for Asian and Caucasian populations, as well as across all races. To ascertain any significant racial differences, the 95% CIs of the pooled ORs were compared. Study heterogeneity was evaluated using Cochran’s Q-test, with a *P*-value < 0.1 indicating significant heterogeneity. For single nucleotide variation (SNV) with more than five included studies, publication bias was assessed using Egger’s regression and funnel plot analysis. For genetic variants reported in more than three studies, the stability and sensitivity of the meta-analyses were gauged using a one-study-removed analysis, where pooled ORs were recalculated omitting one study at a time to check the consistency of the meta-analysis results. All analyses were performed using R Statistical Software (v4.0.5; R Core Team 2021), employing the ‘meta’ package^33^.

### Haplotype and linkage disequilibrium analysis

The haplotype and linkage disequilibrium (LD) analysis for this study involved an in silico all-populations based approach. The SNPs included in the study were initially clustered by chromosomes. Subsequently, each cluster containing two or more SNPs underwent analysis to ascertain if there were haplotype blocks linking these SNPs. This analysis was facilitated using the online tools LDhap and LDmatrix, accessible at https://ldlink.nci.nih.gov/?tab=home.

## Results

### Characteristics of eligible studies

**Figure 1:**
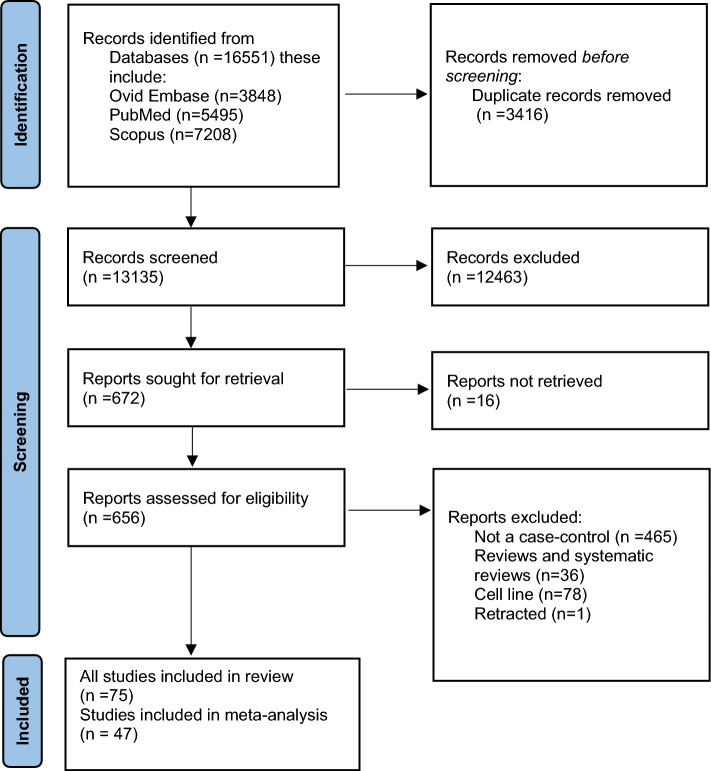
Preferred reporting items for systematic reviews and meta-analyses (PRISMA) flowchart.

A total of 16,551 potentially relevant studies were initially identified including 5495 from MEDLINE , 3848 from Ovid Embase, and 7208 from Scopus. Of which, 3416 were duplicate studies and 12,463 records were excluded after assessing the title and abstract. We sought to retrieve 672 reports of which 16 were unavailable. After evaluating the full text of 656 articles, only 75 articles were eligible. Among these 56 studies (74.6% of the eligible studies) were performed in the Chinese population, 6 studies in the United States (US), 5 studies in Italy, 1 study in Russia, 1 study in Iran and Brazil, and 2 studies each had 2 cohorts one from Spain and one from Slovenia with separate estimates reported for each cohort. Preferred Reporting Items for Systematic Reviews and Meta-Analyses (PRISMA) flowchart is shown in Fig. 1. The characteristics of Included Studies are available in Supplementary material Table S1. A summary of the study characteristics is shown in Supplementary Material Table S2.

### Characteristics of the retrieved genetic variants

The eligible articles reported 190 genetic polymorphisms across 79 genes: 184 Single-nucleotide polymorphism (SNPs), 4 Deletion/Insertion (DELINS), and 2 short tandem repeats (STRs). Detailed information on all the genetic variations is provided in Supplementary Material Table S3.

Based on the number of different genetic variations studied, the 8 most studied genes were: TP53(14 different SNPs), IGF2R (7different SNPs), NAT2 (7different SNPs), VEGF (6 different SNPs), BMP2, ERBB2/HER2, GRM4, and PRKCG each (5 different SNPs).

### Meta-analyses of genetic variations associated with osteosarcoma

#### Results of main and leave-one-out analyses

Only 48 genetic-association studies were included in this meta-analysis. At least two independent studies were available for 37 genetic variations (35 SNPs and 2 DELINS) in 23 different genes allowing us to perform 183 meta-analyses. We used a random-effects model to calculate the pooled odds ratio (OR) and 95% confidence for the 35 genetic variants included in the meta-analysis. The pooled ORs of 12 variants in 8 genes (CTLA-4, ERCC3, IL-8, PRCKG, RECQL5, TNF-α, XRCC3, and VEGF) were significantly associated with the risk of osteosarcoma in the main analysis. While MDM2, 2 SNP were only significantly associated with the risk of osteosarcoma in the sensitivity analysis. We calculated pooled OR for 5 genetic models (homozygous, heterozygous, dominant, recessive, and allele). We also investigated the effect of the race with separate estimates for Asians and Caucasians reported. Table 1 shows the genetic variants associated with osteosarcoma in the combined analysis or in one of the populations. Supplementary Material Table S4 shows the OR for genetic variants not associated with osteosarcoma.

**Table 1:** Genetic variants associated with osteosarcoma.

SNV	chr	Model	OR (95% CI) P-value	I^2^%	Q- value	Heterogeneity P-value	Cases versus Controls	#Studies in Asian	Asian OR (95% CI)	#Studies Caucasian	Caucasian OR (95% CI)
VEGF rs699947	6	CA versus CC	1.33 [1.10; 1.61] 0.018	0	0.86	0.83	709 versus 874 (4)	4^14,42–44^	1.33 [1.10; 1.61	0	
		AA versus CC	1.89[1.31; 2.72] 0.012	0	1.69	0.64	709 versus 874 (4)	4^14,42–44^	1.89[1.31; 2.72]	0	
		CA + AA versus CC	1.46[1.16; 1.84]0.014	0	1.48	0.69	709 versus 874 (4)	4^14,42–44^	1.46[1.16; 1.84	0	
		AA versus CC + CA	1.64 [1.24; 2.16] 0.011	0	1.10	0.77	709 versus 874 (4)	4^14,42–44^	1.64 [1.24; 2.16]	0	
		C versus. A	1.40 [1.16; 1.70] 0.011	0	1.9	0.59	709 versus 874 (4)	4^14,42–44^	1.40 [1.16; 1.70]	0	
VEGF rs3025039	6	CT versus CC	1.15 [1.08; 1.22]0.0008	0	1.24	0.99	1913 versus 2302 (9)	9^14,42–49^	1.15 [1.08; 1.22]	0	
		TT versus CC	1.73 [1.38; 2.16] 0.0005	0	4.83	0.77	1913 versus 2302 (9)	9^14,42–49^	1.73 [1.38; 2.16]	0	
		CT + TT versus CC	1.25 [1.17; 1.33 < 0.0001	0	0.56	0.99	1913 versus 2302 (9)	9^14,42–49^	1.25 [1.17; 1.33	0	
		TT versus CC + CT	1.61[0.92; 2.82] 0.09	75.3	32.37	< 0.0001	1913 versus 2302 (9)	9^14,42–49^	1.61[0.92; 2.82]	0	
		T versus C	1.27 [1.18; 1.36] < 0.0001	0	2.83	0.94	1913 versus 2302(9)	9^14,42–49^	1.27 [1.18; 1.36]	0	
VEGF rs1570360	6	GA versus AA	1.16 [0.95; 1.42] 0.08	0	0.25	0.88	527 versus 692 (3)	3^14,43,44^	1.16 [0.95; 1.42]	0	
		GG versus AA	1.44 [1.33; 1.55] 0.0023	0	0.02	0.99	527 versus 692 (3)	3^14,43,44^	1.44 [1.33; 1.55]	0	
		GA + GG versus AA	1.24 [0.996; 1.54]0.051	0	0.36	0.83	527 versus 692 (3)	3^14,43,44^	1.24 [0.996; 1.54]	0	
		GG versus AA + GA	1.36 [1.29; 1.43] 0.002	0	0.01	0.99	527 versus 692 (3)	3^14,43,44^	1.36 [1.29; 1.43]	0	
		G versus A	1.24 [1.02; 1.5] 0.042	0	0.47	0.79	527 versus 692 (3)	3^14,43,44^	1.24 [1.02; 1.5]	0	
VEGF rs2010963	6	CG versus CC	1.30 [1.13; 1.50] 0.004	0	2.61	0.86	1489 versus 1867(7)	7^14,43–45,48,49^	1.30 [1.13; 1.50]	0	
		GG versus CC	1.56 [1.12; 2.18] 0.018	37	69.55	0.15	1489 versus 1867(7)	7^14,43–45,48,49^	1.56 [1.12; 2.18]	0	
		CG + GG versus CC	1.35 [1.094; 1.65]0.013	13	6.93	0.33	1489 versus 1867(7)	7^14,43–45,48,49^	1.35 [1.094; 1.65]	0	
		GG versus CG + CC	1.47 [0.90; 2.50] 0.12	85	39.44	< 0.0001	1489 versus 1867(7)	7^14,43–45,48,49^	1.47 [0.90; 2.50]	0	
		G versus C	1.26 [1.06; 1.49] 0.017	44	10.69	0.098	1489 versus 1867(7)	7^14,43–45,48,49^	1.26 [1.06; 1.49]	0	
VEGF rs10434	6	GA versus GG	1.08 [0.98; 1.18] 0.11	0	0.92	0.97	1167 versus 1524 (6)	6^14,42–45,49^	1.08 [0.98; 1.18]	0	
		AA versus GG	1.21 [1.07; 1.37]] 0.012	0	0.81	0.98	1167 versus 1524 (6)	6^14,42–45,49^	1.21 [1.07; 1.37]]	0	
		GA + AA versus GG	1.12 [1.01; 1.21] 0.034	0	0.91	0.96	1167 versus 1524 (6)	6^14,42–45,49^	1.12 [1.01; 1.21]	0	
		AA versus GG + GA	1.17 [1.08; 1.27]0.004	0	041	0.99	1167 versus 1524 (6)	6^14,42–45,49^	1.17 [1.08; 1.27]	0	
		G versus A	1.10 [1.04; 1.17] 0.01	0	0.83	0.97	1167 versus 1524 (6)	6^14,42–45,49^	1.10 [1.04; 1.17]	0	
CTLA-4 rs5742909	2	CT versus CC	1.31 [0.06; 27.42] 0.47	50	2.01	0.16	389 versus 413 (2)	2^17,50^	1.31 [0.06; 27.42]	0	
		TT versus CC	2.5 [1.4; 4.4]0.032	0	0.01	0.91	389 versus 413 (2)	2^17,50^	2.5 [1.4; 4.4]0.032	0	
		CT + TT versus CC	1.41 [0.085; 23.48] 0.36	0	1.9	0.168	389 versus 413 (2)	2^17,50^	1.41 [0.085; 23.48]	0	
		TT versus CC + CT	2.30[1.71;3.08]0.017	47	0.001	0.95	389 versus 413 (2)	2^17,50^	2.30[1.71;3.08]	0	
		T versus C	1.43 [0.178; 11.47] 0.28	31	1.45	0.23	389 versus 413 (2)	2^17,50^	1.43 [0.178; 11.47]	0	
CTLA-4 rs231775	2	GA versus GG	1.02 [0.37; 2.8] 0.94	50	5.96	0.11	693 versus 754 (4)	3^17,50,51^	1.25 [0.89; 1.75]	1^26^	0.28 [0.08;1.001]
		AA versus GG	1.93 [0.96; 3.91] 0.06	0	2.79	0.43	693 versus 754 (4)	3^17,50,51^	2.24 [2.14; 2.34]	1^26^	0.72 [0.20; 2.59]
		GA + AA versus GG	0.69 [0.06; 7.80] 0.66	92	36.54	< 0.0001	693 versus 754 (4)	3^17,50,51^	1.40 [1.04; 1.88]	1^26^	2.40 (0.70–8.18)
		AA versus GG + GA	0.75[0.5;1.11] 0.1	0	2.17	0.54	693 versus 754 (4)	3^17,50,51^	1.98 [1.73; 2.26]	1^26^	2.24[1.21;4.15]
		G versus A	1.1[0.5; 2.4] 0.72	87	22.26	< 0.0001	693 versus 754 (4)	3^17,50,51^	1.38 [1.20; 1.59]	1^26^	1.36[0.87; 2.14]
ERCC3 rs4150506	19	CT versus CC	1.26 [1.04; 1.53]0.04	0	0.02	0.90	522 versus 1047(2)	2^52,53^	1.26 [1.04; 1.53]	0	
		TT versus CC	1.78 [1.20; 2.63] 0.004	0	0.40	0.53	522 versus 1047(2)	2^52,53^	1.78 [1.20; 2.63]	0	
		CT + TT versus CC	1.35 [1.09; 1.67] 0.006	0	0.1	0.75	522 versus 1047(2)	2^52,53^	1.35 [1.09; 1.67]	0	
		TT versus CC + CT	1.62 [1.11; 2.37] 0.01	0	0.47	0.49	522 versus 1047(2)	2^52,53^	1.62 [1.11; 2.3]	0	
		C versus T	1.33 [1.12; 1.57] 0.001	0	0.33	0.56	522 versus 1047(2)	2^52,53^	1.33 [1.12; 1.57]	0	
IL-8 rs4073	4	TA versus TT	1.60[0.99; 2.6] 0.0501	0	0.04	0.85	299 versus 299 (2)	2^54,55^	1.60[0.99; 2.6]	0	
		AA versus TT	1.95 [1.53; 2.50] 0.02	0	0.01	0.94	299 versus 299 (2)	2^54,55^	1.95 [1.53; 2.50]	0	
		TA + AA versus TT	1.70 [1.05; 2.73] 0.05	0	0.001	0.96	299 versus 299 (2)	2^54,55^	1.70 [1.05; 2.73]	0	
		AA versus TA + TT	1.67[1.39;2.01] 0.02	0	0.001	0.95	299 versus 299 (2)	2^54,55^	1.67[1.39;2.01]	0	
		A versus T	1.6 [1.03; 2.49] 0.05	0	0.07	0.8	299 versus 299 (2)	2^54,55^	1.6 [1.03; 2.49]	0	
IL-6 rs1800795	7	GC versus GG	1.09 [0.79; 1.51] 0.53	88	8.57	0.003	286 versus 366 (2)	1^56^	1.57 [1.04; 2.36]	1^57^	0.55 [0.31; 1.002]
		CC versus GG	1.0 [0.72; 1.54] 0.77	85	6.77	0.0093	286 versus 366 (2)	1^56^	1.77 [1.02; 3.07]	1^57^	0.63 [0.37; 1.10]
		GC + CC versus GG	1.14 [0.82; 1.59] 0.43	97	33.65	< 0.0001	286 versus 366 (2)	1^56^	2.03 [1.37; 3.0]	1^57^	0.51[0.29;1.005]
		CC versus GG + GC	1.06[0.002;721.8] 0.92	6	6.77	0.0093	286 versus 366 (2)	1^56^	0.45 [030; 1.001]	1^57^	0.70 [0.41; 1.21]
		C versus G	1.09[0.87;1.38]0.50	97	0.95	< 0.0001	286 versus 366 (2)	1^56^	1.76 [1.31; 2.36]	1^57^	0.45 [0.30;1.004]
IL-10 rs1800896	1	AG versus AA	1.23 [0.85; 1.76] 0.27	61	2.6	0.2	340 versus 420 (2)	1^58^	1.07 [0.72; 1.59]	1^57^	2.67 [0.95; 7.50]
		GG versus AA	1.92[0.64;5.75] 0.08	0	0.13	0.72	340 versus 420 (2)	1^58^	1.85 [1.10; 3.09]	1^57^	2.3 [0.79; 6.67]
		AG + GG versus AA	1.38[0.99;1.90] 0.052	0	0.88	0.35	340 versus 420 (2)	1^58^	1.29 [0.91; 1.82]	1^57^	2.02 [0.84; 4.89]
		GG versus AA + AG	1.36[0.03; 57.5] 0.48	58	2.39	0.12	340 versus 420 (2)	1^58^	1.80 [1.10; 3.0]	1^57^	1 [0.57; 1.75]
		G versus A	1.31[0.48;3.60]0.184	0	0.48	0.49	340 versus 420 (2)	1^58^	1.39 [1.06; 1.81]	1^57^	1.17 [0.79; 1.74]
GSTP1 rs1695	11	AG versus AA	1.19 [0.79; 1.81]0.21	0	1.09	0.58	400 versus 783 (3)	1^59^	1.43 [0.93; 2.18]	2^23,60^	1.08 [0.82; 1.40]
		GG versus AA	2.09 [0.64; 6.88] 0.17	21.1	2.54	0.28	400 versus 783 (3)	1^59^	3.33 [1.53; 7.30]	2^23,60^	1.50 [0.60; 3.70]
		AG + GG versus AA	1.30 [0.73 2.31] 0.18	10.3	2.23	0.33	400 versus 783 (3)	1^59^	1.66 [1.11; 2.49]	2^23,60^	1.1276 [0.99; 1.27]
		GG versus AA + AG	1.91 [0.69; 5.31] 0.11	0	1.98	0.37	400 versus 783 (3)	1^59^	2.85 [1.34; 6.05]	2^23,60^	1.44 [0.52; 3.98]
		G versus A	1.31 [0.78; 2.20] 0.15	0	3.06	0.22	400 versus 783 (3)	1^59^	1.6170 [1.19; 2.20]	2^23,60^	1.141 [1.05; 1.24]
PRKCG rs454006	19	CT versus TT	0.96[0.09;10.29]0.86	70	3.34	0.07	998 versus 998 (2)	2^61,62^	0.96[0.09;10.29]	0	
		CC versus TT	1.94 [1.69; 2.23] 0.01	0	0.01	0.94	998 versus 998 (2)	2^61,62^	1.94 [1.69; 2.23]	0	
		CT + CC versus TT	1.20 [0.34; 4.20] 0.32	15	1.18	0.28	998 versus 998 (2)	2^61,62^	1.20 [0.34; 4.20]	0	
		CC versus CT + TT	1.99 [0.82; 4.83] 0.07	0	0.28	0.60	998 versus 998 (2)	2^61,62^	1.99 [0.82; 4.83]	0	
		C versus T	1.35 [1.11; 1.63] 0.03	0	0.05	0.83	998 versus 998 (2)	2^63,64^	1.35 [1.11; 1.63]	0	
RECQL5 rs820196	17	TC versus TT	1.31 [1.11; 1.55] 0.03	0	0.01	0.94	397 versus 441 (2)	2^63,64^	1.31 [1.11; 1.55]	0	
		CC versus TT	2.52 [1.60; 4.0] < 0.0001	0	0.15	0.70	397 versus 441 (2)	2^63,64^	2.52 [1.60; 4.0]	0	
		TC + CC versus TT	1.49[1.03; 2.14] 0.05	0	0.04	0.84	397 versus 441 (2)	2^63,64^	1.49[1.03; 2.14]	0	
		CC versus TT + TC	2.15 [1.41; 3.29] 0.0004	0	0.15	0.79	397 versus 441 (2)	2^63,64^	2.15 [1.41; 3.29]	0	
		C versus T	1.44 [1.19; 1.76] 0.0003	0	0.11	0.74	397 versus 441 (2)	2^63,64^	1.44 [1.19; 1.76]	0	
TNF-α rs1800629	6	GA versus GG	1.18 [0.46; 3.05] 0.52	16	2.38	0.30	227 versus 370 (3)	1^65^	1.06 [0.56; 2.01]	2^57,66^	1.26 [0.77; 2.06]
		AA versus GG	3.46 [0.40; 29.90] 0.09	0	0.17	0.67	160 versus 259 (2)	1^65^	4.0 [1.41; 11.38]	1^57^	2.84 [0.83; 9.68]
		GA + AA versus GG	1.37 [0.50; 3.78] 0.31	33	2.98	0.23	227 versus 370 (3)	1^65^	1.39 [0.76; 2.53]	2^57,66^	1.37 [0.86; 2.18]
		AA versus GG + GA	3.26 [0.22; 48.28] 0.11	0	0.29	0.60	160 versus 259 (2)	1^65^	1.39 [0.76; 2.53]	1^57^	1.37 [0.86; 2.18]
		A versus G	1.46 [0.54; 3.93] 0.24	44	3.57	0.17	227 versus 370 (3)	1^65^	1.61 [1.04; 2.50]	2^57,66^	1.46 [0.54; 3.93]
TNF-α *rs*361525	6	GA versus GG	0.53 [0.28; 0.98] 0.04	70	3.34	0.07	143 versus 210 (2)	1^65^	0.80 [0.39; 1.63]	1^57^	0.17[0.04; 0.76]
		AA versus GG*	NA								
		GA + AA versus GG*	NA								
		AA versus GG + GA*	NA								
		A versus G	0.65 [0.38; 1.124]0.124		4.03	0.045	143 versus 210 (2)	1^65^	0.95 [0.51; 1.76]	1^57^	0.18 [0.04; 0.8]
TP53 rs1042522	17	CG versus CC	0.79 [0.34; 1.82] 0.43	75	12.0	0.01	615 versus 844 (4)	1^16^	1.50 [1.03; 2.20]	3^60,67,68^	0.59 [0.36; 1.001]
		GG versus CC	1.50 [0.66; 3.38] 0.21	31.6	4.38	0.22	615 versus 844 (4)	1^16^	1.89 [1.16; 3.07]	3^60,67,68^	1.16 [0.26; 5.08]
		CG + GG versus CC	0.98[0.49; 1.93] 0.91	66.2	8.90	0.03	615 versus 844 (4)	1^16^	1.57 [1.10; 2.25]	3^60,67,68^	0.77 [0.40; 1.50]
		GG versus CC + CG	1.70 [0.70; 4.13] 0.15	0	2.84	0.41	615 versus 844 (4)	1^16^	1.47 [0.96; 2.26]	3^60,67,68^	1.91 [0.28; 13.23]
		G versus C	1.182 [0.74; 1.88] 0.34	51.4	6.18	0.10	615 versus 844 (4)	1^16^	1.38 [1.09; 1.75]	3^60,67,68^	1.09 [0.47; 2.56]
XRCC3 rs861539	14	CT versus CC	1.37 [1.34; 1.39]0.0002	0	0.001	0.97	484 versus 910(3)	2^69,70^	1.36 [1.26; 1.47]	1^60^	1.37 [0.94; 1.99]
		TT versus CC	1.69 [1.2; 2.39]0.003	63.7	5.51	0.064	484 versus 910(3)	2^69,70^	2.57 [1.85; 3.59]	1^60^	1.12 [0.68; 1.83]
		CT + TT versus CC	1.47 [0.17; 12.47] 0.65	97.2	144	< 0.0001	711 versus 1581(5)	3^69–71^	1.56 [1.15; 2.11]	2^60,72^	0.98 [0.79; 1.22]
		TT versus CC + CT	1.40 [1.02; 1.91] 0.04	71.6	7.05	0.03	484 versus 910(3)	2^69,70^	2.22 [1.571; 3.15]	1^60^	0.93 [0.60; 1.45]
		T versus C	1.32 [1.12; 1.56] 0.001	52.7	4.22	0.12	484 versus 910(3)	2^69,70^	1.56 [1.18; 2.07]	1^60^	1.11 [0.87; 1.41]

*AA genotype is 0 in both cases and controls in one study. No pooling of ORs was possible.

Five SNPs across the Vascular Endothelial Growth factor gene (VEGF) were significantly associated with the risk of osteosarcoma under one or more genetic models. VEGF rs699947, VEGF rs1570360, and VEGF rs2010963 were significant under the 5 investigated genetic models. VEGF rs10434 was significant under all models except the heterozygous model while VEGF rs3025039 was significant under the homozygous model, recessive model, and allele model.

In leave-one-out analysis, VEGF rs699947 and VEGF rs1570360, pooled OR estimates were not stable and were insignificant with the removal of some studies. Conversely, VEGF rs3025039 and VEGF rs2010963 estimates were stable and were still associated with an increased risk of osteosarcoma in the sensitivity analysis. As for rs10434, the pooled estimates were stable for the allele and homozygous model and unstable for all other models. Supplementary material Figure S2.

IL-8 rs4073 was significantly associated with an increased risk of osteosarcoma under all genetic models except the heterozygous model. RecQ Like Helicase 5 (RECQL5) rs820196 was associated with an increased risk of osteosarcoma under all genetic models.

Cytotoxic T-lymphocyte associated protein 4 (CTLA-4) rs5742909 was significantly associated with osteosarcoma risk under the homozygous model (TT vs. CC) OR 2.5 95% CI = 1.4–4.4, *P* = 0.032. CTLA-4 rs231775 was not associated with risk of osteosarcoma in the main analysis but was significantly associated with osteosarcoma risk in the leave-one-out analysis with a decrease in heterogeneity and significance with the removal of Bilbao-Aldaiturriaga^26^ In subgroup analysis, the test of subgroup differences was significant indicating that there is a significant effect of race on the pooled OR (Spanish vs. Chinese). Table 1 and Supplementary material Figs. S1 and S2.

X-ray repair cross-complementing 3 (XRCC3) rs861539 under all genetic models except the dominant model, OR 1.47 95% CI = 0.17–12.47, *P* = 0.03; In subgroup analysis, the test of subgroup differences was significant (*p*-value < 0.001), indicating that there is a significant effect of race on the estimate with XRCC3 rs861539 significantly associated with osteosarcoma in Asians but not Caucasians. Table 1 and. Supplementary material Fig. S2.

Tumor necrosis factor-alpha (TNF-α) rs361525 was significantly associated with decreased risk of osteosarcoma under the heterozygous model (the only model investigated due to unavailability of allele frequency), GA vs GG: OR 0.53 95% CI = 0.28–0.98, *P* = 0.04. However, this association is only significant in Caucasians. (TNF-α) rs1800629 was not associated with osteosarcoma in the main analysis but showed a significant association with osteosarcoma in Asians under the homozygous and allele mode.

PRKCG rs454006 was significantly associated with an increased risk of osteosarcoma under the homozygous and the allele models in the main analysis. IL-6 was not significantly associated with the risk of osteosarcoma in main or sensitivity analysis, however, race affects the estimate, with a significant increase in the risk of osteosarcoma observed in Asians but not in Caucasians. The same was observed in TP53 rs1042522 and GSTP1 rs1695, however, in GSTP1 rs1695, the sub-group analysis comprised only one study in Asians. As for IL-10 rs1800896, race effect was also observed, however, with increased risk of osteosarcoma observed in Asians under the homozygous, recessive, and allele model but not in Caucasians, but, with only one study reported in each race and a non-significant p-value for subgroup analysis, the race effect cannot be concluded.

MDM2 rs1690916 was not significantly associated with the risk of osteosarcoma in the main analysis but in the leave-one-out sensitivity analysis, rs1690916 was significantly associated with a decreased risk of osteosarcoma under the allele and the dominant model. MDM2 rs2279744 was not associated with the risk of osteosarcoma in the main analysis. However, in the sensitivity analysis, upon the omission of Bilbao-Aldaiturriaga^26^, rs2279744 was significantly associated with an increased risk of osteosarcoma. Table 1 and Supplementary Material Fig. S2.

All other genetic variants investigated in the meta-analysis were not significantly associated with the risk of osteosarcoma in the main or sensitivity analysis. Table 1, Supplementary material Fig. S1.

#### Results after removal of studies with poor quality

Quality assessment with done by 2 authors independently (MA and AA) with a third author consulted (OH) in case of discrepancies. Eighteen papers reporting on 14 SNPs were deemed of poor quality and were removed in the sensitivity analysis (VEGF rs699947, VEGF rs833061, and VEGF rs1570360, TNF-α rs361525 TNF-α rs1800629, CTLA-4 rs231775, CTLA-4 s5742909, GSTM1, GSTM3 rs138440339, GSTP1 rs1695, GSTT1, XRCC3 rs861539, RAD51 rs1801320, RAD51rs1801321, RAD51rs1259335, NBN rs1805794, MDM2 rs1690916, IL-6 rs1800795 and IL-10 rs1800795). (Table S1) After omitting these papers, only 4 SNPs had 2 or more papers (MDM2 rs1690916, GSTT1, GSTM1, and CTLA-4 rs231775. The results of the meta-analysis were not affected by the omission except VEGF rs699947, and VEGF rs1570360 which became insignificant, and CTLA-4 rs231775, which showed a significant association with osteosarcoma after the omission which is consistent with having a race effect since all the remaining papers reported on the association in the Chinese population.

#### Results after removal of studies deviating from HWE

**Figure 2:**
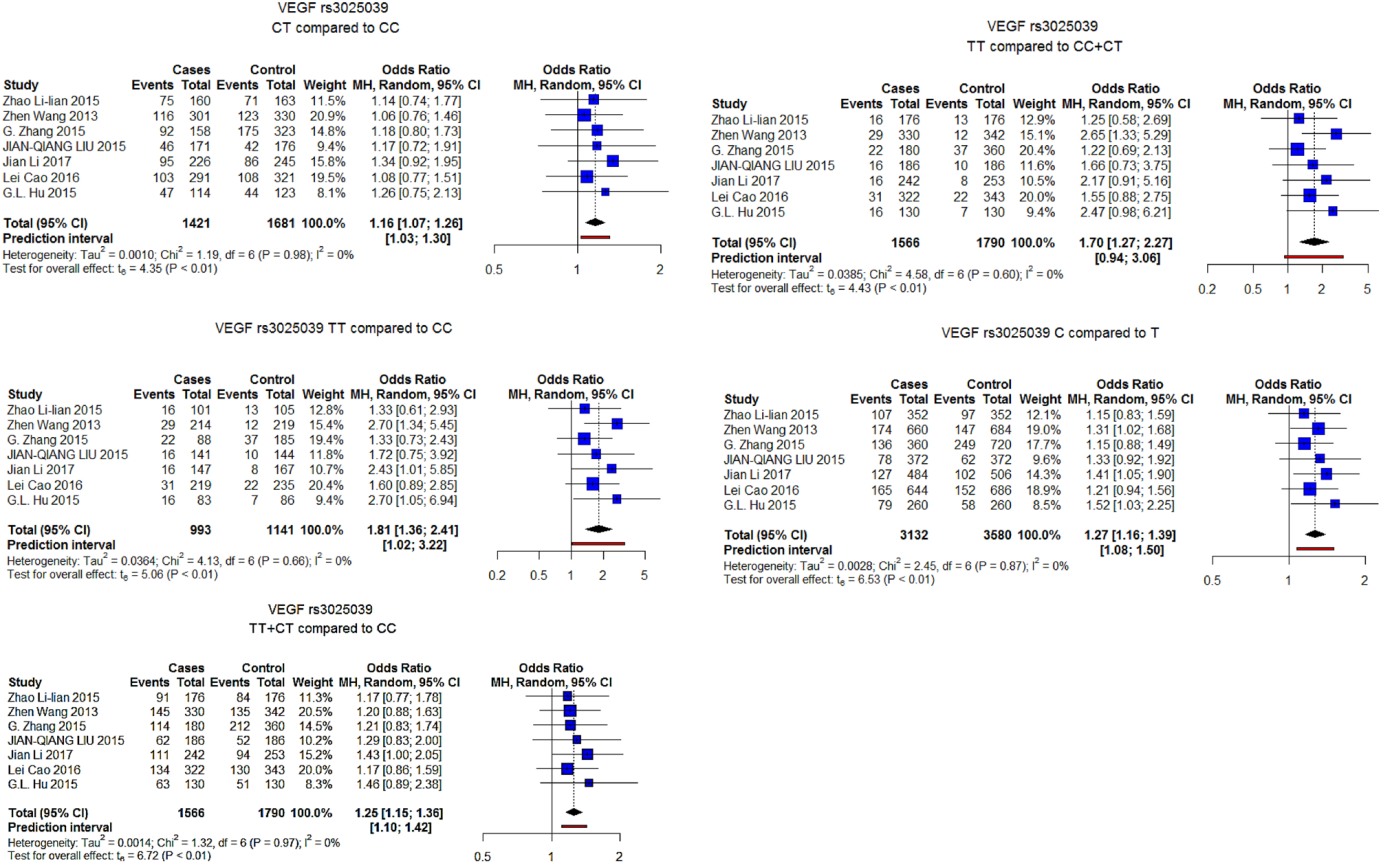
Association of VEGF rs3025039 with osteosarcoma under different genetic models considering only studies not deviating from HWE.

**Figure 3:**
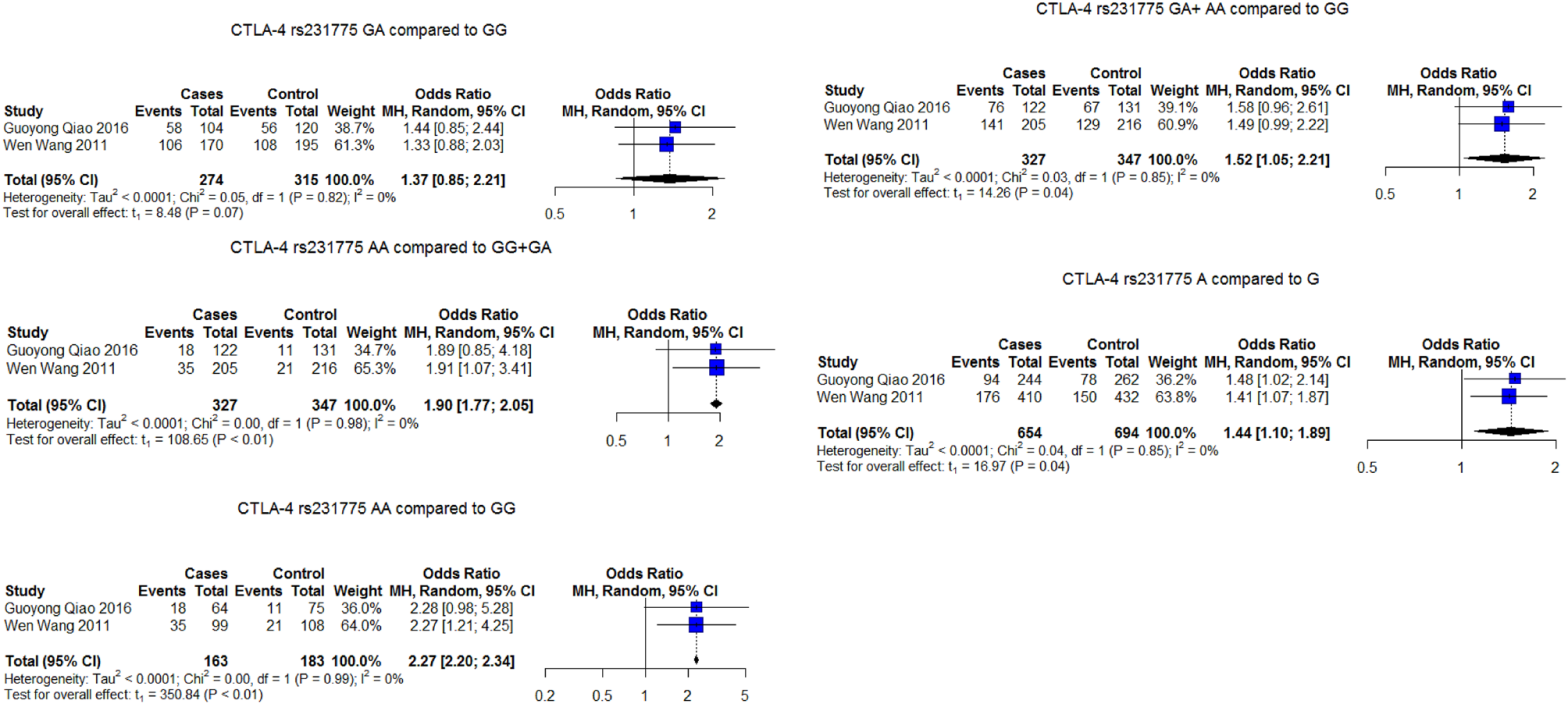
Association of CTLA-4 rs231775 with osteosarcoma under different genetic models considering only studies not deviating from HWE.

Of the studies included in the meta-analysis, six SNPs reported in 9 studies showed deviation from Hardy–Weinberg equilibrium (HWE) among control subjects. After removing these studies, the only SNPs with more than 2 studies available for analysis were VEGF rs3025039 and CTLA-4 rs231775. The pooled ORs for VEGF rs3025039 were significant under all genetic models with the omission of 2 studies deviating from HWE. Figure 2. As for CTLA-4 rs231775, although it was not associated with the risk of osteosarcoma in the main analysis, the omission of studies showing the deviation caused the SNV to be significantly associated with increased risk of osteosarcoma under all genetic models except the heterozygous model. Figure 3.

### Haplotype and linkage disequilibrium analysis

Included SNPs were segregated to 13 chromosomes, chr1, chr2, chr4, chr6, chr7, chr8, chr11, chr12, chr14, chr15, chr17, chr19 and chr22. Out of those 13 chromosomal clusters, only 8 chromosomes were found to have two or more SNPs from our studied SNPs; chr1, chr2, chr6, chr12, chr14, chr15, chr17, and chr19.

Based on the LDmatrix tool results out of those 4 LD blocks were identified: the first block was in chromosome 6 and linkage disequilibrium was detected between rs699947, rs833061, and rs1570360, the second block was in chromosomes 15 and the linkage disequilibrium was between rs1801321 and rs12593359, the third block was in chromosome 17 and the linked SNPs were rs1042522 and rs1642785 and the 4th block was detected in chromosome 19 and the linked SNPs were rs1800470 and rs1800469.

#### Publication bias

**Figure 4:**
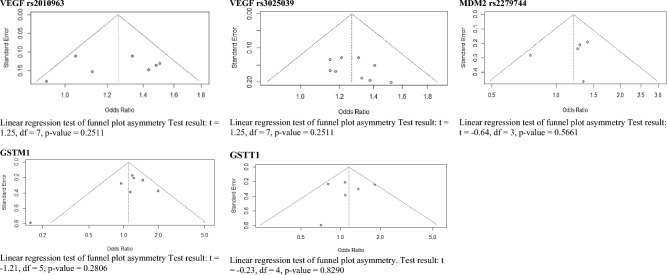
Funnel plots and Harbord's score test for funnel plot asymmetry for 5 variants with more than 5 studies: VEGF rs2010963, VEGF rs3025039, MDM2 rs2279744, GSTT1, and GSTM1.

Funnel plots and Harbord's score test for funnel plot asymmetry were performed to assess the publication bias for 5 variants with more than 5 studies: VEGF rs2010963, VEGF rs3025039, MDM2 rs2279744, GSTT1, and GSTM1. None of them showed a significant publication bias. Figure 4.

## Discussion

In this comprehensive meta-analysis, we explored the relationship between genetic variants and osteosarcoma risk. Our main analysis identified significant associations with osteosarcoma for 12 variants in 8 genes: CTLA-4, ERCC3, IL-8, PRCKG, RECQL5, TNF-α, XRCC3, and VEGF. Variants such as CTLA-4 rs231775 and VEGF rs699947 were linked to an increased risk, while others like IL-8 rs4073 indicated a decreased risk. The stability of these associations, except for a few such as VEGF rs699947 and rs1570360, which showed variability, was confirmed through additional analysis.

When studies deviating from Hardy–Weinberg Equilibrium (HWE) were omitted, CTLA-4 rs231775 emerged as significantly associated with an increased risk of osteosarcoma. A leave-one-out analysis revealed the instability of certain estimates, such as VEGF rs699947, VEGF rs1570360, and MDM2 rs1690916, which could not be explained by race effect in some polymorphisms.

Haplotype and linkage disequilibrium analyses of the included SNPs were performed to determine if any LD blocks could account for the SNPs' association with osteosarcoma and if these SNPs belong to a defined haplotype. Our results indicated that 9 SNPs are located in 4 linkage disequilibrium blocks. All meta-analyses tests of significance were in concordance with the LD status, except for VEGF rs699947, VEGF rs1570360, and VEGF rs833061. Despite strong linkage in all populations, and specifically in the Chinese population, the test of significance showed that rs699947 and rs1570360 were significantly associated with the risk of osteosarcoma and rs833061 was non-significant, contradicting their complete linkage. The removal of two poor-quality studies in the sensitivity analysis led to a change in the significance of VEGF rs699947 and rs1570360, reflecting concordance between the test of significance and the linkage disequilibrium status.

In prior meta-analyses, associations of genetic polymorphisms in VEGF, MDM2, CTLA-4, TNF-a, TNF-b1, PRCKG, RECQL5, XRCC3, and GST with osteosarcoma susceptibility were investigated^24,27,28,34–38^. We adopted distinct inclusion and exclusion criteria focusing on SNPs associated with osteosarcoma in population-based case–control studies and investigated racial variations in genetic susceptibility.

In total, 75 studies describing 190 polymorphisms across 79 genes were reviewed. Two or more studies were available for only 37 genetic variations across 23 genes of which 33 polymorphisms in 21 genes were reported in Asians and 20 polymorphisms in 15 genes in Caucasians. It’s worth noting that 75% of the eligible studies were conducted in the Chinese population, with limited studies in Caucasians and no studies in other ethnic groups. This highlights the need for future studies in other ethnicities to expand our perspective of the gene variants associated with osteosarcoma. The available studies provide evidence that race can significantly affect the association of certain polymorphisms with the risk of osteosarcoma.

This discrepancy in risk association may provide an explanation for the difference in the incidence of osteosarcoma by race^39^. A similar effect of race on the association of polymorphisms and risk of cancer was reported in gastric cancer^22^. This evident race effect from this study and from similar studies in other diseases prompts caution when combining data from different races.

The functional implications of the identified SNPs significantly associated with osteosarcoma suggest that these genetic variants potentially influence key biological processes related to cancer development and progression. Variants in genes like CTLA-4, ERCC3, and TNF-α might impact immune regulation and inflammatory responses, crucial in tumor microenvironment dynamics. SNPs in genes such as PRCKG, RECQL5, and XRCC3 are likely to affect cellular signaling and DNA repair mechanisms, contributing to genetic instability. Additionally, SNPs in VEGF could alter angiogenesis, influencing tumor growth and metastasis^16–18,40^. This multifaceted genetic influence underscores the complexity of osteosarcoma's etiology and highlights the importance of further research to elucidate the precise molecular mechanisms for targeted therapeutic strategies.

One limitation of this review is the quality of the retrieved studies. The quality of a systematic review is partly related to the quality of the studies included in the quantitative analysis. Unfortunately, a non-negligible number of studies retrieved were of poor or moderate quality, emphasizing the need to improve the reporting of genetic association studies.

Our ability to draw conclusions was also limited by the sample size, with few studies (3 or fewer) reporting on the same genetic variant, and thus type I and II error may have affected the results. This limited sample size may also have an impact on our ability to estimate heterogeneity and hence, the random-effects model was adopted being more conservative and providing wider confidence intervals as compared to the fixed-effect model^41^. It is important to note that heterogeneity in our meta-analysis might stem from diversity in the study populations, particularly in terms of age and other demographic or clinical characteristics. However, the ability to explore these potential sources of heterogeneity through subgroup analysis or meta-regression was limited. A significant number of the included studies did not offer detailed demographic or clinical data, thereby restricting our capacity to conduct such analyses. It’s worth noting that all the included studies have a case–control design, and though this design is most useful for the meta-analysis, it limits the ability to identify novel biomarkers.

In conclusion, this meta-analysis identified SNPs associated with the risk of osteosarcoma, emerging as potential biomarkers. These markers could provide critical insights into the likelihood of osteosarcoma occurrence and progression. Such information is invaluable for early detection and risk assessment, paving the way for more personalized and targeted therapeutic approaches. Additionally, understanding the variations in these genetic markers might also shed light on differential responses to osteosarcoma treatments, thereby assisting in the refinement of treatment regimens. Importantly, these variants may have prognostic implications, offering predictions about disease outcomes and survival rates. This aspect holds considerable significance for clinical decision-making and patient counseling, particularly in the context of a disease as complex as osteosarcoma. It must be emphasized, however, that the practical application of these findings in a clinical setting hinge on their validation in clinical trials and further studies. The integration of these genetic markers into clinical protocols has the potential to substantially alter the current management strategies for osteosarcoma, steering them towards more personalized and efficacy-driven treatments.

However, in consideration of our current meta-analytic findings, it is imperative to highlight the necessity for external validation through independent cohorts or additional datasets. This step is crucial for affirming the reliability and generalizability of our results, particularly given the intricate nature of genetic associations. Future investigative efforts should be directed towards employing large-scale genomic databases, to test the applicability of our findings across a broader population spectrum.

While our meta-analysis primarily focused on identifying SNPs associated with osteosarcoma, we acknowledge that the interplay between genetic predispositions and environmental factors could significantly impact disease risk and progression^42^. Due to lack of relevant data from the original studies, we could not explain gene–environment interactions. Future research should aim to incorporate comprehensive data that allows for the analysis of gene-environment interactions which will enable a more holistic understanding of osteosarcoma etiology and could lead to more effective prevention strategies. Future studies should also integrating multi-omics data, including transcriptomics, epigenomics, and proteomics, to complement and expand upon the genetic findings. By combining genetic information with insights into gene expression, epigenetic modifications, and protein-level changes, a more comprehensive understanding of the molecular mechanisms driving osteosarcoma can be achieved. This integrated approach has the potential to uncover novel therapeutic targets and facilitate the development of personalized treatment strategies, addressing the complexity of this disease and ultimately improving patient outcomes.

This study presents the most up-to-date evidence for osteosarcoma susceptibility variants emphasizing the need for further large-scale studies to identify new variants and validate these associations. It also highlights the effect of race on these associations highlighting the need for race-specific genetic risk panels and, illuminating the complex interplay of genetics and ethnicity in osteosarcoma, thus advancing the field towards more nuanced and personalized therapeutic strategies. However, further studies with broader multiethnic groups and exploration into the possible biological significance of these genetic variations in osteosarcoma is warranted.

### Supplementary Information

Supplementary Information.

## Data Availability

The datasets generated during the current study are available from the corresponding author.
