# Supplementary material for: Genetic variants associated with osteosarcoma risk: a systematic review and meta-analysis
Source: Sci Rep. 2024 Feb 15;14:3828. doi: 10.1038/s41598-024-53802-w (PMC10869693; doi:10.1038/s41598-024-53802-w)

**Supplementary Material**

**Genetic variants associated with Osteosarcoma risk: A systematic review and meta-analysis**

Omneya Hassanain^1*^; Mahmoud Alaa^2^; Mohamed K. Khalifa^3^; Nehal Kamal^2^; Aseel Albagoury ^2^; Ahmed M. El Ghoneimy^4,5^

1 Epidemiology and Biostatistics Unit, Clinical Research, Children’s Cancer Hospital Egypt-57357 (CCHE-57357), Cairo, Egypt,

2 Basic Research, Children’s Cancer Hospital Egypt-57357 (CCHE-57357), Cairo, Egypt

3 Molecular Pathology Laboratory, Children’s Cancer Hospital Egypt-57357 (CCHE-57357), Cairo, Egypt

4 Department of Orthopedic Oncology, Children’s Cancer Hospital-57357 (CCHE-57357), Cairo, Egypt,

5 Department of Orthopedics, Faculty of Medicine, Cairo University, Cairo, Egypt

***Corresponding Author:** Omneya Hassanain, Children’s Cancer Hospital 57357, 1 Seket el Emam, el Sayeda Zeinab, 11441, Cairo, Egypt, Email: Omneya.hassanain@57357.org, ORCID 0000-0001-5912-6773

**Search Terms**

# Search strategy for MEDLINE/PubMed

1. For Osteosarcoma the following MeSH headings and text words will be used:

osteosarcoma OR osteosarcoma*OR osteogenic sarcoma OR sarcoma, osteogenic OR sarcomas, osteogenic

1. For genetic variation the following MeSH headings and text words will be used:

Disease Susceptibility OR Genetic Variation OR Genetic Association Studies OR Sequence Analysis OR mutation OR Polymorphism, Genetic OR single nucleotide polymorphism OR SNP OR variant OR variation OR risk OR locus

The search thread is as follows:

(((OR "Disease Susceptibility"[Mesh]) OR "Genetic Variation"[Mesh]) OR "Genetic Association Studies"[Mesh] OR "Genotype"[Mesh] OR "Sequence Analysis"[Mesh] OR mutation[Text Word] OR Polymorphism, Genetic[Text Word] OR single nucleotide polymorphism[Text Word] OR SNP[Text Word] OR variant[Text Word] OR variation [Text Word] OR risk[Text Word] OR "Causality"[Mesh] OR locus[Text Word]) AND ((("Neoplasms, Bone Tissue"[Mesh] OR "Osteosarcoma"[Mesh] OR osteosarcoma OR osteosarcoma* OR osteogenic sarcoma OR sarcoma, osteogenic OR sarcomas, osteogenic)))

# Search strategy for Ovid/Embase

1. For Osteosarcoma

bone tumors.mp. or bone tumor/ or osteosarcoma/ or osteogenic sarcoma.mp. or osteosarcoma/or osteosarcoma cell/ or osteosarcoma cell/ or osteosarcoma/ or bone sarcoma.mp. or osteosarcoma/

1. for genetic variation

Disease Susceptibility.mp. or disease predisposition/ or Genetic Variation.mp. or genetic variability/ or genetic variation/ or genetics/ or Genetic Association Studies.mp. or genetic association study/ or genotype/ or Genotype.mp. /or Sequence Analysis.mp. or sequence analysis/ or exp mutation/ or Polymorphism, Genetic.mp. or genetic polymorphism/ or DNA polymorphism/ or single nucleotide polymorphism/ or genetic variability/ or genetic association/ or single nucleotide polymorphism/ or variant.mp. or genetic association/ or single nucleotide polymorphism/ or gene locus/

# Search strategy for Scopus

KEY ( osteosarcoma ) AND SUBJAREA ( bioc ) AND (genetics) OR ( disease AND susceptibility ) OR (disease AND predisposition) OR (genetic AND variation) OR (genetic AND variability) OR (genotype) OR (genetic AND association AND studies) OR (sequence AND analysis) OR (genetic AND polymorphism) OR (single nucleotide polymorphism) OR (genetic AND association) OR (gene AND locus) OR (variant) AND ( LIMIT-TO ( LANGUAGE , "English" ) ) AND ( LIMIT-TO ( EXACTKEYWORD , "Human" ) OR LIMIT-TO ( EXACTKEYWORD , "Humans" ) )

**PRISMA 2020 Checklist**

| **Section and Topic** | **Item #** | **Checklist item** | **Location where item is reported** |
| --- | --- | --- | --- |
| **TITLE** | | |  |
| Title | 1 | Identify the report as a systematic review. | Page 1 |
| **ABSTRACT** | | |  |
| Abstract | 2 | See the PRISMA 2020 for Abstracts checklist. | Page 2 |
| **INTRODUCTION** | | |  |
| Rationale | 3 | Describe the rationale for the review in the context of existing knowledge. | Page 3 |
| Objectives | 4 | Provide an explicit statement of the objective(s) or question(s) the review addresses. | Page 3 |
| **METHODS** | | |  |
| Eligibility criteria | 5 | Specify the inclusion and exclusion criteria for the review and how studies were grouped for the syntheses. | Page 4 |
| Information sources | 6 | Specify all databases, registers, websites, organisations, reference lists and other sources searched or consulted to identify studies. Specify the date when each source was last searched or consulted. |  |
| Search strategy | 7 | Present the full search strategies for all databases, registers and websites, including any filters and limits used. | Page 4 |
| Selection process | 8 | Specify the methods used to decide whether a study met the inclusion criteria of the review, including how many reviewers screened each record and each report retrieved, whether they worked independently, and if applicable, details of automation tools used in the process. | Page 4 |
| Data collection process | 9 | Specify the methods used to collect data from reports, including how many reviewers collected data from each report, whether they worked independently, any processes for obtaining or confirming data from study investigators, and if applicable, details of automation tools used in the process. | Page 4 |
| Data items | 10a | List and define all outcomes for which data were sought. Specify whether all results that were compatible with each outcome domain in each study were sought (e.g. for all measures, time points, analyses), and if not, the methods used to decide which results to collect. | Page 4 |
|  | 10b | List and define all other variables for which data were sought (e.g. participant and intervention characteristics, funding sources). Describe any assumptions made about any missing or unclear information. | Page 4 |
| Study risk of bias assessment | 11 | Specify the methods used to assess risk of bias in the included studies, including details of the tool(s) used, how many reviewers assessed each study and whether they worked independently, and if applicable, details of automation tools used in the process. | Page 4, 5 |
| Effect measures | 12 | Specify for each outcome the effect measure(s) (e.g. risk ratio, mean difference) used in the synthesis or presentation of results. | Page 5 |
| Synthesis methods | 13a | Describe the processes used to decide which studies were eligible for each synthesis (e.g. tabulating the study intervention characteristics and comparing against the planned groups for each synthesis (item #5)). | Page 4 |
|  | 13b | Describe any methods required to prepare the data for presentation or synthesis, such as handling of missing summary statistics, or data conversions. | Page 5 |
|  | 13c | Describe any methods used to tabulate or visually display results of individual studies and syntheses. | Page 5 |
|  | 13d | Describe any methods used to synthesize results and provide a rationale for the choice(s). If meta-analysis was performed, describe the model(s), method(s) to identify the presence and extent of statistical heterogeneity, and software package(s) used. | Page 5 |
|  | 13e | Describe any methods used to explore possible causes of heterogeneity among study results (e.g. subgroup analysis, meta-regression). | Page 5 |
|  | 13f | Describe any sensitivity analyses conducted to assess robustness of the synthesized results. | Page 5 |
| Reporting bias assessment | 14 | Describe any methods used to assess risk of bias due to missing results in a synthesis (arising from reporting biases). | Page 4 |
| Certainty assessment | 15 | Describe any methods used to assess certainty (or confidence) in the body of evidence for an outcome. | Page 4,5 |
| **RESULTS** | | |  |
| Study selection | 16a | Describe the results of the search and selection process, from the number of records identified in the search to the number of studies included in the review, ideally using a flow diagram. | Page 5,6 Figure 1 |
|  | 16b | Cite studies that might appear to meet the inclusion criteria, but which were excluded, and explain why they were excluded. | 5,6,Figure 1 |
| Study characteristics | 17 | Cite each included study and present its characteristics. | Supplementary File C1 |
| Risk of bias in studies | 18 | Present assessments of risk of bias for each included study. | Supplementary File C1 |
| Results of individual studies | 19 | For all outcomes, present, for each study: (a) summary statistics for each group (where appropriate) and (b) an effect estimate and its precision (e.g. confidence/credible interval), ideally using structured tables or plots. | Supplementary File C3 |
| Results of syntheses | 20a | For each synthesis, briefly summarise the characteristics and risk of bias among contributing studies. | Page 7,8,9,10 |
|  | 20b | Present results of all statistical syntheses conducted. If meta-analysis was done, present for each the summary estimate and its precision (e.g. confidence/credible interval) and measures of statistical heterogeneity. If comparing groups, describe the direction of the effect. | Page 6,7,8,9  Supplementary File C4, Table 1 |
|  | 20c | Present results of all investigations of possible causes of heterogeneity among study results. | Page 6,7,8 |
|  | 20d | Present results of all sensitivity analyses conducted to assess the robustness of the synthesized results. | Page 6,7,8,9 |
| Reporting biases | 21 | Present assessments of risk of bias due to missing results (arising from reporting biases) for each synthesis assessed. | Page 10 |
| Certainty of evidence | 22 | Present assessments of certainty (or confidence) in the body of evidence for each outcome assessed. | Page 7,8,9,10 |
| **DISCUSSION** | | |  |
| Discussion | 23a | Provide a general interpretation of the results in the context of other evidence. | Page 10, 11 |
|  | 23b | Discuss any limitations of the evidence included in the review. | Page 11 |
|  | 23c | Discuss any limitations of the review processes used. | Page 11 |
|  | 23d | Discuss implications of the results for practice, policy, and future research. | Page 11,12 |
| **OTHER INFORMATION** | | |  |
| Registration and protocol | 24a | Provide registration information for the review, including register name and registration number, or state that the review was not registered. | Page 3 |
|  | 24b | Indicate where the review protocol can be accessed, or state that a protocol was not prepared. | Page 3 |
|  | 24c | Describe and explain any amendments to information provided at registration or in the protocol. | NA |
| Support | 25 | Describe sources of financial or non-financial support for the review, and the role of the funders or sponsors in the review. | Page 12 |
| Competing interests | 26 | Declare any competing interests of review authors. | Page 12 |
| Availability of data, code and other materials | 27 | Report which of the following are publicly available and where they can be found: template data collection forms; data extracted from included studies; data used for all analyses; analytic code; any other materials used in the review. | Page 12 |

*From:*  Page MJ, McKenzie JE, Bossuyt PM, Boutron I, Hoffmann TC, Mulrow CD, et al. The PRISMA 2020 statement: an updated guideline for reporting systematic reviews. BMJ 2021;372:n71. doi: 10.1136/bmj.n71

For more information, visit: <http://www.prisma-statement.org/>

**Supplementary Table**

*Table S1:Characteristics of Included Studies*

| **First Author year** | **Journal** | **location** | **Genotyping method** | **Age cases** | **Age controls** | **M/F cases** | **M/F controls** | **Source of Controls** | **Quality Assessment** |
| --- | --- | --- | --- | --- | --- | --- | --- | --- | --- |
| Mirabello 2010[1] | Carcinogenesis | USA | Custom Infinium BeadChip | means(sd)  26.6 (16) | means(sd)  60.9 (9.9) | 56/43 | 872/493 | mixed | moderate |
| HE 2013[2] | Asian Pac J Cancer Prev | China | taqman (PCR) | 21.3±11.1 | 31.3 ±16.6 | 35/24 | 33/30 | Hospital based | moderate |
| Xiao 2017[3] | Sci Rep | China | taqman (PCR) |  |  | 213/155 | 355/261 | Hospital based | good |
| Hattinger 2016[4] | Oncotarget. | Italy | PSQ/taqman assay | < 14 years 60  ≥ 14 years 136 | median  age (16 years) | 122/74 | 285/185 | Hospital based | moderate |
| Zhao 2014[5] | Biomed Res Int. | China | PCR |  |  | 154/93 | 265/163 | Hospital based | moderate |
| Cong 2015[6] | Tumour Biol | China | taqman (PCR) | 28.0 ± 18.0 | 27.8 ± 17.2 | 132/80 | 226/180 | Hospital based | moderate |
| Wang 2011[7] | DNA and Cell Biology | China | (PCR-RFLP) &sequencing | - | - | 119/86 | 113/103 | Hospital based | moderate |
| Zhang 2019[8] | Med Sci Monit. | China | taqman (PCR) | 15.44±3.03 | 15.96±3.24 | 97/87 | 99/86 | Hospital based | moderate |
| He 2014[9] | Tumour Biol. | China | taqman (PCR) | 25.5 ± 12.6 | 28.4 ± 14.0 | 120/69 | 105/90 | Hospital based | moderate |
| Guo 2015[10] | Int J Clin Exp Pathol | China | PCR-RFLP | - | - | 90/51 | 208/133 | Hospital based | moderate |
| Bi 2016[11] | Sci.reports | China | Taqman PCR | - | - | 139/97 | 229/189 | Hospital based | moderate |
| Qiao 2016[12] | IJCEM | China | PCR-RFLP | - | - | 74/48 | 79/52 | Population based | poor |
| Liu 2011[13] | DNA Cell Biol | China | PCR-RFLP | - | - | 172/95 | 156/126 | Hospital based | moderate |
| Wang 2011[14] | Genet Test Mol Biomarkers | China | PCR-RFLP | - | - | 119/86 | 113/103 | Hospital based | moderate |
| Bilbao-Aldaiturriaga 2017 [15] | Neoplasma | Spain | PCR-RFLP | - | - | - | - | Hospital based | poor |
| Jin 2015[16] | Pak J Med Sci | China | PCR-RFLP | 17.7±8.2 | 19.2±5.3 | 91/57 | 181/115 | Hospital based | moderate |
| Xu 2017[17] | Mamm Genome | China | taqman (PCR) |  |  | 208/173 | 425/340 | Hospital based | good |
| Biason 2011[18] | Pharmacogenomics J | Italy | taqman (PCR) | Median 16 range(4–68) | median  age 35 years | 79/51 | 156/101 | Hospital based | moderate |
| Gómez-Díaz [19] | Oncol Lett | México | taqman (PCR) | Median 20.5, range (9-68) |  | 12/16 |  | Hospital based | poor |
| Ma 2016[20] | Genet Mol Res | China | PCR-RFLP | >20 total 53  <20 total 88 | >20 total 162  <20 total 82 | 87/54 | 174/108 | Hospital based | moderate |
| Wang 2015[21] | Tumour Biol. | China | taqman (PCR) | >20 total 48  <20 total 78 | >20 total 90  <20 total 78 | 70/56 | 88/80 | Hospital based | moderate |
| Jiang 2014[22] | Med Oncol. | China | sequencing | 20.09 ± 9.32 | 20.53 ± 11.79 | 98/70 | 134/82 | Hospital based | moderate |
| Barnette 2004[23] | Cancer Epidemiol Biomarkers Prev. | USA | fluorescent, allele-specific  PCR |  |  |  |  | Population based | poor |
| Salinas-Souza 2010[24] | Pharmacogenet Genomics | Brazil | PCR-RFLP | >15 total 40  <15 total 40 | >15 total 83  <15 total 77 | 47/33 | 94/66 | Hospital based | moderate |
| Lu2011[25] | APJCP | China | TaqMan PCR | 13.6±3.2 | 13.8±2.9 | 66/44 | 130/96 | Hospital based | moderate |
| Li 2015[26] | Genetics and molecular research | China | PCR |  |  |  |  | Hospital based | poor |
| Qu 2016[27] | Genetics and molecular research | China | PCR-RFLP | >20 total 50  <20 total 103 | >20 total 150  <20 total 102 | 61/61 | 138/114 | Hospital based | poor |
| Moghimi 2018[28] | APJCP | Iran | PCR-RFLP |  |  |  |  | Population based | poor |
| HE 2017[29] | Eur Rev Med Pharmacol Sci | China | Taqman PCR | >20 total 89  <20 total 104 | >20 total 198  <20 total 185 | 113/80 | 230/153 | Hospital based | moderate |
| Xin 2015[30] | Int J Clin Exp Pathol. | China | MassARRAY system | Median 19.6  Range(16-53) | Median 20.3  Range(12-51) | 53/37 | 59/41 | Hospital based | poor |
| Zhou 2016[31] | Oncotarget. | China | Sequenom MassARRAY | Meam 23.5±8.5 | Mean 23.3±7.1 | 537/363 | 533/367 | Hospital based | moderate |
| Savage 2007[32] | Cancer Epidemiol Biomarkers | USA | Taqman PCR |  |  |  |  | Hospital based | moderate |
| He 2014[33] | Int Orthop. | China | PCR-RFLP | Mean(SD) 23.7(12.8) | Mean(SD) 24.1(13) | 68/52 | 64/56 | Hospital based | moderate |
| Shang 2017[34] | Biomedical Research | China | PCR-RFLP | 28.5 ± 13.1 | 27.3 ± 17.9 | 150/41 | 158/49 | Hospital based | poor |
| Qi 2016[35] | Tumour Biol. | China | PCR-RFLP | (mean ± SD) 23.4 (±6.4) | (mean ± SD) 22.5 (±7.9) | 118/88 | 105/101 | Hospital based | poor |
| Oliveira 2007[36] | J Pediatr Hematol Oncol  . | Brazil | PCR-RFLP | First decade 8  Second decade 61  Third decade 10  Fouth decade |  | 47/33 |  | Hospital based | poor |
| Tian 2016[37] | Int J Clin Exp Med. | China | PCR-RFLP | >20 total 45  <20 total 64 | >20 total 50  <20 total 59 | 60/49 | 56/53 | Hospital based | moderate |
| Chen 2016[38] | Tumour Biol | China | PCR-RFLP | >20 total 82  <20 total 108 | >20 total 81  <20 total 99 | 91/99 | 96/94 | Hospital based | moderate |
| Cui 2016[39] | Tumour Biol | China | PCR-RFLP | 23.7 (10.2) | 24.9 (11.8) | 168/92 | 143/117 | Hospital based | moderate |
| Wang 2013[40] | DNA Cell Biol  . | China | PCR-RFLP | >20 total 34  <20 total 72 | >20 total 58  <20 total 152 | 68/38 | 130/80 | Population based | moderate |
| Tang 2016[41] | Sci Rep | China | PCR-RFLP | Median(range)  18.0 (6.0–58.0) | Median(range) 20.0 (12.0–59.0) | 216/142 | 241/161 | Hospital based | moderate |
| Tang 2014[42] | Medicine | China | PCR-RFLP | Mean 27.5±8.5 | Mean 27.9±7.8 | 105/55 | 155/95 | Population based | moderate |
| Yang 2014[43] | Med Oncol | China | Taqman PCR | Mean ± SD  21.25 ± 11.04 | Mean ± SD  30.99 ± 16.57 | 70/48 | 66/60 | Hospital based | moderate |
| Liu 2012[44] | PLoS One | China | PCR-RFLP | >20 total 94  <20 total 232 | >20 total 128  <20 total 305 | 188/138 | 252/181 | Hospital based | moderate |
| Bilbao-Aldaiturriaga 2016[45] | Pediatr Res | Spain | ARMS and RFLP |  |  |  |  | Hospital based | moderate |
| Bilbao-Aldaiturriaga 2016[45] | Pediatr Res | Slovenia | ARMS and RFLP |  |  |  |  | Hospital based | moderate |
| Naumov 2012[46] | Bull Exp Biol Med | Russia | MALDI-TOF minisequencing | 16-64 years |  | 36/32 |  | Not specified | poor |
| Mirabello 2011[47] | BMC Cancer | USA | Custom Infinium Beadchip | mean age (SD) 26.6(16.5) | mean age (SD) 24.7(15.1) | 54/42 | 34/29 |  | moderate |
| Toffoli 2009[48] | Clin Cancer Res  . | Italy | Pyrosequencing | Median 16 years (range, 4-73). | Range 18-40 | 123/78 |  | Population based | moderate |
| Cui 2016[49] | Biomarkers | China | PCR-RFLP | Mean(±SD)  23.49 (10.23) | Mean(±SD)  24.26 (11.38) | 149/102 | 153/98 | Hospital based | moderate |
| Zhi 2016[50] | J Cancer | China | MALDI–TOF | 21.00±4.7 | 21.02±4.3 | 146/138 | 498/483 | Hospital based | good |
| Huang 2015[51] | Onco Targets Ther. | China | Taqman PCR | Mean ± SD  15.90±3.37 | Mean ± SD  16.24±3.30 | 151/109 | 159/127 | Hospital based | moderate |
| Goricar 2015[52] | J Med Biochem. | Slovenia | PCR-RFLP | Median (range)  19 (15–38) | Median  (range)  30 (23–53.5) | 40/37 | 219/153 | Population based | poor |
| Zhang 2014[53] | Tumour Biol | China | TaqMan (PCR) | 24.4 ± 8.7 | 24.3 ± 8.8 | 365/245 | 320/300 | Population based | good |
| Lu 2015[54] | Tumour Biol | China | Sequencing |  |  | 208/180 | 193/195 | Hospital based | good |
| Zhi 2014[55] | Tumour Biol | China | Taqman PCR | 42.3±12.4 | 43.1±13.1 | 121/101 | 130/110 | Hospital based | moderate |
| Dong 2015[56] | Genet Mol Res. | China | Taqman PCR | 32.3 ± 12.1 | 33.1 ± 12.4 | 97/88 | 101/100 | Hospital based | poor |
| Wu 2015[57] | Tumour Biol | China | Taqman PCR | 21.25±11.04 | 30.99±16.57 | 66/58 | 78/58 | Hospital based | moderate |
| Xu 2014[58] | DNA Cell Biol  . | China | PCR-RFLP | >20 total 65  <20 total 137 | >20 total 66  <20 total 150 | 125/77 | 127/89 | Hospital based | moderate |
| Zhao 2015[59] | Int J Clin Exp Pathol. | China | PCR-RFLP | 14.68 (5-22 years) | 15.6(12-24) | 57/23 | 54/46 | Hospital based | poor |
| Patino-Garcia 2000[60] | J Medical Genetics | Spain | PCR |  |  |  |  | Hospital based | good |
| Savage 2007[61] | Pediatr Blood Cancer | USA | taqman PCR | Mean(SD)  26.4 (16.2) | Mean(SD)  24.9 (14.5) | - | - | Hospital based | good |
| Ru 2015[62] | Int J Clin Exp Pathol | china | taqman PCR | 28.0 ± 18.0 | 27.9 ± 17.3 | 130/80 | 240/180 | Hospital based | moderate |
| Zhang 2018[63] | Cancer Biol Ther. | China | taqman PCR | - | - | 690/610 | 675/625 | Not specified | moderate |
| Zhang 2015[64] | Genet Mol Res. | China | PCR-RFLP | 20.6 ± 11.6 | 21.4 ± 12.1 | 106/76 | 106/63 | Population based | poor |
| Zhao 2015[65] | Pak J Med Sci. | China | PCR-RFLP | 18.4±11.5 | 19.5±10.8 | 109/67 | 109/67 | Hospital based | poor |
| Tie 2014[66] | Int J Clin Exp Pathol  . | China | PCR-RFLP | 19.1 ± 6.7 | 19.6 ± 7.2 | 108/108 | 215/115 | Hospital based | moderate |
| Liu 2015[67] | Oncol Lett  . | China | PCR-RFLP | 18.5±10.3 | 19.2±11.8 | 114/72 | 114/72 | Hospital based | moderate |
| Wang 2013[68] | Tumour Biol. | China | PCR-RFLP | 27.3 ± 13.6 | 28.2 ± 14.3 | 188/142 | 176/166 | Population based | moderate |
| Zhang 2015[69] | Genet Mol Res | China | PCR-RFLP | ≤20y 123  >20y 57 | ≤20y 236  >20y 124 | 110/70 | 220/140 | Population based | moderate |
| Li 2017[70] | Biomedical Research | China | MALDI-TOF MS | 26.1 ± 16.6 | 28.1 ± 12.2 | 118/124 | 122/131 | Hospital based | moderate |
| Cao 2016[71] | Int J Clin Exp Pathol | China | PCR-RFLP | 18.1±10.2 | 19.3±10.3 | 231/91 | 242/101 | Hospital based | good |
| Hu 2015[72] | Genet Mol Res. | China | PCR-RFLP | <20 80  ≥20 50 | <20 77  ≥20 53 | 77/76 | 76/54 | Hospital based | moderate |
| Guo 2015[73] | Genet Mol Res. | China | PCR-RFLP | 17.82 ± 5.40 | 17.31 ± 5.52 | 84/52 | 77/59 | Hospital based | poor |
| Yang 2015[74] | Int J Clin Exp Pathol. | China | PCR-RFLP | <20years 94  ≥ 20 years 58 | <20years 182  ≥20 years 122 | 88/64 | 176/128 | Hospital based | moderate |
| Koshkina 2007[75] | J Pediatr Hematol Oncol. | USA | PCR-RFLP | 13.0±4.0 | 49.1±11.4 | 70/35 | 245/265 | Population based | moderate |

*Table S2: Summary of characteristics of included studies*

| Total number of studies | 75 |
| --- | --- |
| * Location of study:  Brazil  China  Iran  Italy  Russia  Slovenia  Spain  USA | 1(1.3%)  56(74.7%)  1(1.3%)  5(6.7 %)  1(1.3%)  2(2.7%)  2(2.7%)  6(8%) |
| Genotyping Methods  Genotyping method  PCR-RFLP  TaqMan PCR  PCR  Custom Infinium BeadChip  MALDI-TOF minisequencing  Sequencing  (PCR-RFLP) &sequencing  ARMS and RFLP  fluorescent, allele-specific  MALDI–TOF  MassARRAY system  PSQ/taqman assay  Pyrosequencing  Sequenom MassARRAY | 36(48%)  23(30.7%)  2(2.7%)  2(2.7%)  2(2.7%)  2(2.7%)  1(1.3%)  1(1.3%)  1(1.3%)  1(1.3%)  1(1.3%)  1(1.3%)  1(1.3%)  1(1.3%)  1(1.3%) |
| Type of sample  Blood  peripheral blood DNA, mouthwash DNA, or buccal swab DNA collection or Guthrie Card samples | 74(98.7%)  1(1.3%) |
| Type of controls  Hospital based  Population-based  Not specified | 61(81.3%)  12(16%)  3(34%) |
| Controls cases ratio  <1:1  1:1  2:1  >2:1 | 2(2.7%)  40(53.3%)  22(29.3%)  11(14.7%) |
| Cases  Median  Minimum  Maximum | 168  12  1300 |
| Controls  Median  Minimum  Maximum | 240  60  1518 |

| *Table S3: Summary of all genetic variations* | **Gene** | **chr** | **Polymorphism** | **Functional Consequence** | **First Author year** | **Journal** | **location** | **Cases/**  **Controls** | **Genotype frequency cases** | **Genotype frequency controls** | **HWE** | **Genetic Model** | **P-value** |
| --- | --- | --- | --- | --- | --- | --- | --- | --- | --- | --- | --- | --- | --- |
|  | CASC21 | 8 | rs185852 | Intron variant | Mirabello 2010[1] | Carcinogenesis | USA | 99/1430 | GG,AG,AA  60,31,8 | GG,AG,AA  934,440,54 | 0.8 | AG vs GG  AA vs GG | 0.69  0.039 |
|  | CASC8 | 8 | rs896324 | Intron variant, Genic downstream transcript variant | Mirabello 2010[1] | Carcinogenesis | USA | 99/1430 | AA,AG,GG  76,21,2 | AA,AG,GG  1225,191,13 | 0.1 | AG vs AA  GG vs AA | 0.025  0.19 |
|  |  |  | rs10808555 | Intron variant, Genic downstream transcript variant | Mirabello 2010[1] | Carcinogenesis | USA | 99/1430 | AA,AG,GG  37,52,10 | AA,AG,GG  679,595,156 | 0.1 | AG vs AA  GG vs AA | 0.033  0.67 |
|  |  |  | rs17766217 | NA | Mirabello 2010[1] | Carcinogenesis | USA | 99/1430 | TT,TC,CC  47,42,10 | TT,TC,CC  531,692,206 | 0.4 | TC vs TT  CC vs TT | 0.096  0.1 |
|  | 8q24 region | 8 | rs12155672 | NA | Mirabello 2010[1] | Carcinogenesis | USA | 99/1430 | GG,AG,AA  23,42,34 | GG,AG,AA  366,728,336 | 0.5 | AG vs GG  AA vs GG | 0.76  0.098 |
|  |  |  | rs7386167 | NA | Mirabello 2010[1] | Carcinogenesis | USA | 99/1430 | GG,AG,AA  37,40,22 | GG,AG,AA  618,626,186 | 0.2 | AG vs GG  AA vs GG | 0.78  0.018 |
|  | CASC11 | 8 | rs9642880 | Intron variant,  genic downstream transcript variant | Mirabello 2010[1] | Carcinogenesis | USA | 99/1430 | GG,GT,TT  39,42,18 | GG,GT,TT  416,704,306 | 0.8 | GT vs GG  TT vs GG | 0.048  0.11 |
|  | AKT | 7 | rs6973569 | Intron variant, Genic downstream transcript variant | HE 2013[2] | Asian Pac J Cancer Prev | China | 59/63 | AA,AG,GG 20,21,18 | AA,AG,GG  17,24,22 | 0.06 | AG vs AA  GG vs AA | 0.7 |
|  | APEX1 | 14 | rs1130409 | Synonymous variant,  missense variant, coding sequence variant | Xiao 2017[3] | Sci Rep | China | 378/616 | TT,TG,GG  134,178,66 | TT,TG,GG  208,287,121 | 0.2 | TG vs TT  GG vs TT  TG+GG vs TT  G vs T | 0.83  0.4  0.63  0.40 |
|  |  |  |  |  | Hattinger 2016[4] | Oncotarget. | Italy | 196/470 | TT,TG,GG  31,98,67 | TT,TG,GG  94,230,146 | 0.8 | GG  GT  TT | >0.05 |
|  |  |  | rs1760944 | 2KB upstream variant, upstream transcript variant | Xiao 2017[3] | Sci Rep | China | 378/616 | TT,TG,GG  163,163,52 | TT,TG,GG  194,299,123 | 0.7 | TG vs TT  GG vs TT  TG+GG vs TT  G vs T | 0.003  <0.01  <0.01  <0.01 |
|  |  |  | rs17111750 | NA | Xiao 2017[3] | Sci Rep | China | 378/616 | CC,CT,TT  230,111,37 | CC,CT,TT  339,182,41 | 0.02 | CT vs CC  TT vs CC  CT+TTvs CC  T vs C | 0.51  0.27  0.89  0.66 |
|  |  |  | rs1130409 | Synonymous variant, missense variant, coding sequence variant | Hattinger 2016[4] | Oncotarget. | Italy | 196/470 | GG,GT,TT  31,98,67 | GG,GT,TT  94,230,146 | 0.8 | GG  GT  TT | >0.05 |
|  | OSGEP | 14 | rs2275008 | Intron variant | Xiao 2017[3] | Sci Rep | China | 378/616 | TT,TC,CC  276,94,8 | TT,TC,CC  473,128,15 | 0.08 | TC vs TT  CC vs TT  TC+CC vs TT  C vs T | 0.16  0.51  0.2  0.28 |
|  | PIP4P1 | 14 | rs1760941 | 2KB upstream variant, upstream transcript variant | Xiao 2017[3] | Sci Rep | China | 378/616 | CC,CA,AA  213,134,31 | CC,CA,AA  325,230,61 | 0.04 | CA vs CC  AA vs CC  CA+AA vs CC  A vs C | 0.4  0.3  0.29  0.22 |
|  | ARHGAP35 | 19 | rs1052667 | 3 prime UTR variant, genic downstream transcript variant | Zhao 2014[5] | Biomed Res Int. | China | 247/428 | CC,CT,TT  133,70,44 | CC,CT,TT  341,72,15 | <0.01 | CT vs CC  TT vs CC  CT+TTvs CC  T vs C | <0.001  <0.001  <0.001  <0.001 |
|  | BMP2 | 20 | rs3178250 | 3 prime UTR variant | Cong 2015[6] | Tumour Biol | China | 203/406 | CC,CT,TT  35,113,54 | CC,CT,TT  107,219,80 | 0.09 | CT vs CC  TT vs CC  CT+TTvs CC | 0.006 |
|  |  |  | rs235768 | Coding sequence variant,synonymous variant, missense variant | Cong 2015[6] | Tumour Biol | China | 203/406 | TT, TA, AA  123,71,8 | TT, TA, AA  241,148,17 | 0.3 | TA vs TT  AA vs TT  TA+AA vs TT | 0.72 |
|  |  |  | rs1005464 | Intron variant | Cong 2015[6] | Tumour Biol | China | 203/406 | GG,GA,AA 112,77,13 | GG,GA,AA 181,177,48 | 0.6 | GA vs GG  AA vs GG  GA+AA vs GG | 0.05 |
|  |  |  | rs235770 | NA | Cong 2015[6] | Tumour Biol | China | 203/406 | CC,CT,TT  82,102,18 | CC,CT,TT  169,199,38 | 0.06 | CT vs CC  TT vs CC  CT+TTvs CC | 0.92 |
|  |  |  | rs235764 | Intron variant | Cong 2015[6] | Tumour Biol | China | 203/406 | GG,GA,AA 120,72,10 | GG,GA,AA  248,140,18 | 0.8 | GA vs GG  AA vs GG  GA+AA vs GG | 0.66 |
|  | CD86 | 3 | rs1129055 | Coding sequence variant, missense variant | Wang 2011[7] | DNA and Cell Biology | China | 205/216 | GG,GA,AA 27,105,73 | GG,GA,AA 46,113,57 | 0.5 | GA vs GG  AA vs GG  A vs G | 0.097  0.008  0.011 |
|  | CDKN2B | 9 | rs1063192 | 3 prime UTR variant, intron variant | Zhang 2019[8] | Med Sci Monit. | China | 184/185 | GG,GA,AA  54,88,42 | GG,GA,AA  52,89,44 | 0.6 | GA vs GG  AA vs GG  GA+AA vs GG | 0.329  0.321  0.421 |
|  |  |  | rs3217992 | 3 prime UTR variant, intron variant | Zhang 2019[8] | Med Sci Monit. | China | 184/185 | GG,GA,AA  114,63,7 | GG,GA,AA  142,42,1 | 0.3 | GA vs GG  AA vs GG  GA+AA vs GG | 0.024  0.006  0.013 |
|  |  |  | r rs3217986 | 3 prime UTR variant, intron variant | Zhang 2019[8] | Med Sci Monit. | China | 184/185 | CC,CA,AA  66,90,28 | CC,CA,AA  69,86,30 | 0.7 | CA vs CC  AA vs CC  CA+AA vs CC | 0.235  0.22  0.311 |
|  | CDKN2B-AS1 | 9 | rs3218009 | intron variant | Zhang 2019[8] | Med Sci Monit. | China | 184/185 | CC,CG,GG  78,83,23 | CC,CG,GG  86,79,20 | 0.8 | CG vs CC  GG vs CC  CG+GG vs CC | 0.311  0.229  0.32 |
|  | CDKN2A-DT | 9 | rs3731257 | 3 prime UTR variant, intron variant | Zhang 2019[8] | Med Sci Monit. | China | 184/185 | CC,CT,TT  115,57,12 | CC,CT,TT  119,53,13 | 0.05 | CT vs CC  TT vs CC  CT+TT vs CC | 0.521  0.771  0.32 |
|  | COL1A1 | 17 | rs1061970 | 3 prime UTR variant | He 2014[9] | Tumour Biol. | China | 189/195 | TT,CT,CC  72,90,27 | TT,CT,CC  114,,57,24 | 0.0003 | TC vs TT  CCs TT  C vsT | 0.02  0.295 0.022 |
|  |  |  | rs2075559 | intron variant | He 2014 [9] | Tumour Biol. | China | 189/195 | CC,CG,GG  27,108,54 | CC,CG,GG  60,84,51 | 0.06 | CG vs CC  GG vs CC  G vs C | 0.027  0.103  0.029 |
|  | COL18A1 | 21 | rs12483377 | Intron variant, coding sequence variant, missense variant, genic downstream transcript variant | Guo 2015[10] | Int J Clin Exp Pathol | China | 141/341 | DD,DN,NN  119,14,8 | DD,DN,NN  312,28,1 | 0.7 | DN vs DD  NN vs DD | 0.43  <0.001 |
|  |  |  | c.4309G> A  (p.D104N) |  | Bi 2016[11] | Sci.reeports | China | 236/418 | WT,HET,VAR  205,23,8 | WT,HET,VAR  376,39,3 |  | WT  HET  VAR | >0.05 |
|  | CTLA4 | 2 | rs5742909 | Upstream transcript variant, 2KB upstream variant | Qiao 2016[12] | IJCEM | China | 122/131 | CC,CT,TT  74,40,8 | CC,CT,TT  97,30,4 | 0.38 | CT vs CC  TT vs CC | 0.598  0.115 |
|  |  |  |  |  | Liu 2011[13] | DNA Cell Biol  . | China | 267/282 | CC,CT,TT  175,77,15 | CC,CT,TT  195,80,7 | 0.38 | CT vs CC  TT vs CC | 0.713  0.057 |
|  |  |  | rs4553808 | Upstream transcript variant, 2KB upstream variant | Liu 2011[13] | DNA Cell Biol  . | China | 267/282 | AA,AG,GG  177,76,14 | AA,AG,GG  197,73,12 | 0.2 | AG vs AA  GG vs AA  AG+GG vs AA  G vs A | 0.447  0.52  0.37  0.33 |
|  |  |  | rs3087243 | Downstream transcript variant ,500B downstream variant | Liu 2011[13] | DNA Cell Biol  . | China | 267/282 | GG,GA,AA  176,77,14 | GG,GA,AA  188,83,11 | 0.6 | GA vs AA  GG vs AA  GG+GA vs AA  G vs A | 0.962  0.459  0.853  0.66 |
|  |  |  | rs231775 | Missense variant, coding sequence variant | Liu 2011[13] | DNA Cell Biol  . | China | 267/282 | GG,GA,AA  120,140,22 | GG,GA,AA  99,128,40 | <0.05 | GA vs GG  AA vs GG | 0.574  0.007 |
|  |  |  |  |  | Wang 2011[14] | Genet Test Mol Biomarkers | China | 205/216 | GG,GA,AA  64,106,35 | GG,GA,AA  87,108,21 | 0.16 | GA vs GG  AA vs GG | 0.178  0.01 |
|  |  |  |  |  | Qiao 2016[12] | IJCEM | China | 122/131 | GG,GA,AA  46,58,18 | GG,GA,AA  64,56,56 | 0.92 | GA vs GG  AA vs GG | 0.178  0.01 |
|  |  |  |  |  | Bilbao-Aldaiturriaga 2017 [15] | Neoplasma | Spain | 99/125 | GG,GA,AA  5,83,37 | GG,GA,AA  6,28,32 | <0.05 | AA vs AG, AA vs GG  AA vs AG+GG | 0.005  0.011 |
|  | ERCC1 | 19 | rs11615 | Coding sequence variant, synonymous variant | Jin 2015[16] | Pak J Med Sci | China | 148/296 | TT,TC+CC  135,46 | TT,TC+CC  63,52 | - | TC+CC vs TT | 0.58 |
|  |  |  |  |  | Xu 2017[17] | Mamm Genome | China | 381/765 | TT,TC,CC  33,149,199 | TT,TC,CC  44,275,446 | 0.91 | TC vs TT  CC vs TT |  |
|  |  |  |  |  | Biason 2011[18] | Pharmacogenomics J | Italy | 130/250 | TT,TC,CC  37,59,30 | TT,TC,CC  86,111,53 | 0.15 | TC vs TT  CC vs TT  TC+CC vs TT | >0.05  0.37  0.35 |
|  |  |  |  |  | Hattinger 2016[4] | Oncotarget. | Italy | 196/470 | TT,TC,CC  67,84,45 | TT,TC,CC  164,221,85 | 0.5 | TT  TC  CC | >0.05 |
|  |  |  |  |  | Gómez-Díaz [19] | Oncol Lett | México | 28/97 | CC,TC,TT  16,9,3 | CC,TC,TT  59,32,6 | 0.6 | GA vs GG  AA vs GG | 0.94  0.42 |
|  |  |  | rs3212981 | Genic downstream transcript variant,  intron variant | Xu 2017[17] | Mamm Genome | China | 381/765 | CC,CA,AA  197,145,39 | CC,CA,AA  442,272,51 | 0.3 | CA vs CC  AA vs CC |  |
|  |  |  | rs3212986 | Genic downstream transcript variant ,coding sequence variant,3 prime UTR variant, missense variant | Jin 2015[16] | Pak J Med Sci | China | 148/296 | GG,GA+AA  79,69 | GG,GA+AA  169,129 | - | GA+AA vs GG | 0.5 |
|  |  |  |  |  | Hattinger 2016[4] | Oncotarget. | Italy | 196/470 | GG,GT,TT  121,63,12 | GG,GT,TT  254,183,33 | 0.99 | GG  GT  TT | >0.05 |
|  | ERCC2 | 19 | rs1799793 | Missense variant, coding sequence variant, non-coding transcript variant | Gómez-Díaz [19] | Oncol Lett | México | 28/97 | CC,CT,TT  74,26,8 | CC,CT,TT  81,23,15 | <0.001 | CT vs CC  TT vs CC  CC+CT vs CC | 0.52  0.24  0.94 |
|  |  |  |  |  | Jin 2015[16] | Pak J Med Sci | China | 148/296 | GG,GA+AA  84,64 | GG,GA+AA  201,97 | - | GA+AA vs GG | 0.03 |
|  |  |  |  |  | Ma 2016[20] | Genet Mol Res | China | 141/282 | AA,AG,GG  60,62,19 | AA,AG,GG  134,117,31 | 0.5 | AG vs AA  GG vs AA  Ag+GG vs AA | 0.45  0.34  0.33 |
|  |  |  |  |  | Hattinger 2016[4] | Oncotarget. | Italy | 196/470 | GG,GA,AA  84,79,33 | GG,GA,AA  207,193,70 | 0.03 | GG  GA  AA | >0.05 |
|  |  |  | rs13181 | Genic downstream transcript variant, coding sequence variant, missense variant,500B downstream variant | Gómez-Díaz [19] | Oncol Lett | México | 28/97 | TT,TG,GG  21,7,0 | TT,TG,GG  64,31,2 | <0.001 | AC vs AA  CC vs AA  AC+CC vs AA | 0.74  0.91  0.79 |
|  |  |  |  |  | Hattinger 2016[4] | Oncotarget. | Italy | 196/470 | TT,TG,GG  78,88,30 | TT,TG,GG  179,216,75 | 0.5 | TT  TG  GG | >0.05 |
|  |  |  |  |  | Ma 2016[20] | Genet Mol Res | China | 141/282 | TT,TG,GG  96,32,13 | TT,TG,GG  206,58,18 | <0.001 | TG vs TT  GG vs TT  TG +GG vs TT | 0.5  0.25  0.29 |
|  |  |  |  |  | Jin 2015[16] | Pak J Med Sci | China | 148/296 | TT, TG+GG  117,62 | TT, TG+GG  181,86 | - | TG+GG vs TT | 0.59 |
|  | ERCC3 | 19 | rs4150441 | intron variant | Xu 2017[17] | Mamm Genome | China | 381/765 | GG,GA,AA  190,152,39 | GG,GA,AA  500,231,34 | 0.32 | GA vs GG  AA vs GG  GA+AA vs GG |  |
|  |  |  |  |  | Ma 2016[20] | Genet Mol Res | China | 141/282 | GG,GA,AA  56,66,19 | GG,GA,AA  123,129,30 | 0.73 | GA vs GG  AA vs GG  GA+AA vs GG |  |
|  |  |  | rs4150506 | intron variant | Xu 2017[17] | Mamm Genome | China | 381/765 | CC,CT,TT  231,133,35 | CC,CT,TT  486,238,41 | 0.12 | CT vs CC  TT vs CC |  |
|  |  |  |  |  | Ma 2016[20] | Genet Mol Res | China | 141/282 | CC,CT,TT  190,152,39 | CC,CT,TT  148,109,29 | 0.43 | CT vs CC  TT vs CC | 0.34  0.25 |
|  | GRM4 | 6 | rs1906953 | intron variant | Wang 2015[21] | Tumour Biol. | China | 126/168 | TT, TC, CC  29,65,32 | TT, TC, CC  60,80,28 | 0.12 | TT(ref)  TC  CC | 0.034 |
|  |  |  |  |  | Jiang 2014[22] | Med Oncol. | China | 168/216 | TT, TC, CC  45,82,41 | TT, TC, CC  39,101,79 | 0.43 | TT(ref)  TC  CC  C vs T | 0.010  0.002 |
|  |  |  | rs2229901 | 3 prime UTR variant | Wang 2015[21] | Tumour Biol. | China | 126/168 | GG,GA,AA  33,94,31 | GG,GA,AA  33,62,41 | 0.3 | GG(ref)  GA  AA | 0.37 |
|  |  |  | rs733457 | intron variant | Wang 2015[21] | Tumour Biol. | China | 126/168 | TT,TG,GG | TT,TG,GG  79,58,31 | 0.001 | TT(ref)  TG  GG | 0.8 |
|  |  |  | rs7591996 | NA | Jiang 2014[22] | Med Oncol. | China | 168/216 | AA,AC,CC  15,70,83 | AA,AC,CC  21.94,101 | 0.9 | AA(ref)  AC  CC | 0.9 |
|  |  |  | rs17206779 | Intron variant, genic downstream transcript variant | Jiang 2014[22] | Med Oncol. | China | 168/216 | TT,TC,CC  24,74,70 | TT,TC,CC  35,96,85 | 0.4 | TT(ref)  TC  CC | 0.83 |
|  | GSTM1 | 1 | rs_GSTM1_gen_null | 2KB upstream variant, upstream transcript variant(DELINS) | Barnette 2004[23] | Cancer Epidemiol Biomarkers Prev. | USA | 12/326 | Null ,Present  2,10 | Null ,Present  183,143 | - | A0 versus 00  B0 versus 00 | 0.038  0.002 |
|  |  |  |  |  | Salinas-Souza 2010[24] | Pharmacogenet Genomics | Brazil | 80/160 | Null ,Present  35,45 | Null ,Present  72,88 | - | Null vs present | 0.9 |
|  |  |  |  |  | Lu2011[25] | APJCP | China | 110/226 | Null ,Present 61,49 | Null ,Present 104,122 |  | Null vs present | >0.05 |
|  |  |  |  |  | Li 2015[26] | Genetics and molecular research | China | 52/79 | Null ,Present 16,36 | Null ,Present  37,42 |  | Null vs present | <0.05 |
|  |  |  |  |  | Qu 2016[27] | Genetics and molecular research | China | 153/252 | Null ,Present  65/88 | Null ,Present  95/157 |  | Null vs present | 0.34 |
|  |  |  |  |  | Moghimi 2018[28] | APJCP | Iran | 51/60 | Null ,Present  22,29 | Null ,Present  24,36 |  | Null vs present |  |
|  |  |  |  |  | Hattinger 2016[4] | Oncotarget. | Italy | 196/470 | Null ,Present  112,84 | Null ,Present  249,221 |  | WT  HET  VAR | >0.05 |
|  | GSTM3 | 1 |  | Non-coding transcript varaint | Barnette 2004[23] | Cancer Epidemiol Biomarkers Prev. | USA | 12/326 | TT,TC,CC  7,5,0 | TT,TC,CC  224,71,5 | 0.817 |  |  |
|  |  |  |  | Non-coding transcript varaint | Moghimi 2018[28] | APJCP | Iran | 51/60 | TT,TC,CC  34,16,1 | TT,TC,CC  38,21,1 | 0.315 |  |  |
|  | GSTP1 | 11 | rs1695 | Coding sequence variant, missense variant | Moghimi 2018[28] | APJCP | Iran | 51/60 | AA,AG,GG  32,16,3 | AA,AG,GG  39,19,2 | 0.8 |  |  |
|  |  |  |  |  | Qu 2016[27] | Genetics and molecular research | China | 153/252 | AA,AG,GG  64,71,19 | AA,AG,GG  135,105,12 | 0.14 | AG vs AA  GG vs AA  AG+GG vs AA | 0.09  0.002  0.02 |
|  |  |  |  |  | Hattinger 2016[4] | Oncotarget. | Italy | 196/470 | AA,AG,GG  94,86,16 | AA,AG,GG  240,202,28 | 0.09 | AA  AG  GG | >0.05 |
|  | GSTT1 | 22 | rs_GSTT1_gen_null | reduced or no protein activity(DELINS) | Barnette 2004[23] | Cancer Epidemiol Biomarkers Prev. | USA | 12/326 | Null ,Present  2,10 | Null ,Present  66.234 |  | Null vs Present |  |
|  |  |  |  |  | Salinas-Souza 2010[24] | Pharmacogenet Genomics | Brazil | 80/160 | Null ,Presen  26,54 | Null ,Present  42,118 |  | Null vs Present | 0.3 |
|  |  |  |  |  | Qu 2016[27] | Genetics and molecular research | China | 153/252 | Null ,Present  70,40 | Null ,Present  111,115 |  | Null vs Present | 0.7 |
|  |  |  |  |  | Lu2011[25] | APJCP | China | 110/226 | Null ,Present  68,85 | Null ,Present  107,145 |  | Null vs Present | <0.05 |
|  |  |  |  |  | Moghimi 2018[28] | APJCP | Iran | 51/60 | Null ,Present 24,26 | Null ,Present  27,34 |  | Null vs Present |  |
|  |  |  |  |  | Hattinger 2016[4] | Oncotarget. | Italy | 196/470 | Null ,Present  31,165, | Null ,Present  89,381 |  | WT  HET  VAR | >0.05 |
|  | H19 | 11 | rs2735971 | Intron variant ,genic downstream transcript variant, genic upstream transcript variant | HE 2017[29] | Eur Rev Med Pharmacol Sci | China | 193/383 | CC,CT,TT  88,94,11 | CC,CT,TT  169,182,32 | 0.8 | CT vs CC  TT vs CC | 0.816  0.674 |
|  |  |  | rs217727 | Intron variant, non- coding transcript variant, genic downstream transcript variant | HE 2017[29] | Eur Rev Med Pharmacol Sci | China | 193/383 | GG,GA,AA  79,102,12 | GG,GA,AA  195,165,23 | 0.1 | GA vs GG  AA vs GG | 0.024  0.004 |
|  |  |  | rs2839698 | upstream transcript variant, noncoding transcript variant,intron variant,2KB upstream variant,genic downstream | HE 2017[29] | Eur Rev Med Pharmacol Sci | China | 193/383 | GG,GA,AA  83,98,12 | GG,GA,AA  178,175,30 | 0.15 | GA vs GG  AA vs GG | 0.112  0.892 |
|  |  |  | rs3024270 | Intron variant ,genic downstream transcript variant | HE 2017[29] | Eur Rev Med Pharmacol Sci | China | 193/383 | GG,GC,CC  85,91,17 | GG,GC,CC  173,179,31 | 0.1 | GC vs GG  CC vs GG | 0.882  0.457 |
|  | ERBB2/HER2 |  | rs1058808 | non-coding transcript variant,coding sequence variant, missense variant,genic downstream transcript variant | Xin 2015[30] | Int J Clin Exp Pathol. | China | 90/100 | CC,CG ,GG  48,28,14 | CC,CG ,GG  69,26,5 | 0.2 | CG vs CC  GG vs CC  C vs G | 0.02  3.00×10^-3^ |
|  |  |  | rs1136201 | Intron variant ,genic downstream transcript variant, coding sequence variant, missense variant | Xin 2015[30] | Int J Clin Exp Pathol. | China | 90/100 | AA,AG ,GG  20,47,23 | AA ,AG ,GG  38,46,16 | 0.7 | AG vs AA  GG vs AA  A vs G | 0.04  0.01 |
|  |  |  | rs2952156 | Genic downstream transcript variant,intron variant | Xin 2015[30] | Int J Clin Exp Pathol. | China | 90/100 | GG,GA,AA  47,26,17 | GG,GA,AA  46,41,13 | 0.4 | GA vs GG  AA vs GG  G vs A | 0.18  0.97 |
|  |  |  | rs1810132 | Intron variant | Xin 2015[30] | Int J Clin Exp Pathol. | China | 90/100 | CC ,CT ,TT  19,39,32 | CC ,CT ,TT  31,41,28 | 0.07 | CT vs CC  TT vs CC  T vs C | 0.26  0.09 |
|  |  |  | rs2952155 | Intron variant | Xin 2015[30] | Int J Clin Exp Pathol. | China | 90/100 | CC ,CT ,TT  33,31,26 | CC ,CT ,TT  26,41,33 | 0.08 | CT vs CC  TT vs CC  T vs C | 0.28  0.15 |
|  | HOTAIR | 12 | rs7958904 | Non-coding transcript variant | Zhou 2016[31] | Oncotarget. | China | 900/900 | GG,GC,CC  524,320,56 | GG,GC,CC  466,346,88 | >0.05 | GC vs GG  CC vs GG | 6.77×10^−4^ |
|  |  |  | rs874945 | None | Zhou 2016[31] | Oncotarget. | China | 400/400 | GG,GA,AA  267,106,27 | GG,GA,AA  270,108,22 | >0.05 | GA vs GG  AA vs GG | 0.614 |
|  |  |  | rs4759314 | Intron variant | Zhou 2016[31] | Oncotarget. | China | 500/500 | AA,AG,GG  423,62,15 | AA,AG,GG  425,64,11 | >0.05 | AG vs AA  GG vs AA | 0.64 |
|  | IGF2R | 6 | rs1570070 | Coding sequence variant, synonymous variant | Savage 2007[32] | Cancer Epidemiol Biomarkers | USA | 140/74  Orthopedic controls | AA,AG,GG  48,42,14 | AA,AG,GG  33,34,7 | 0.7 | Additive  dominant | 0.6  0.8 |
|  |  |  | rs894817 | Coding sequence variant,synonymous variant | Savage 2007[32] | Cancer Epidemiol Biomarkers | USA | 140/74  Orthopedic controls | GG,GA,AA  48,45,11 | GG,GA,AA  34,32,8 | 0.9 | Additive  dominant | 0.99  0.98 |
|  |  |  | rs998075 | Coding sequence variant,synonymous variant | Savage 2007[32] | Cancer Epidemiol Biomarkers | USA | 140/74  Orthopedic controls | GG,GA,AA  18,54,31 | GG,GA,AA  24,40,10 | 0.3 | Additive  dominant | 0.01  0.02 |
|  |  |  | rs998074 | Intron variant | Savage 2007[32] | Cancer Epidemiol Biomarkers | USA | 140/74  Orthopedic controls | CC,CT,TT  18,53,33 | CC,CT,TT  24,40,10 | 0.3 | Additive  dominant | 0.008  0.019 |
|  |  |  | rs629849 | Coding sequence variant,synonymous variant, missense variant | Savage 2007[32] | Cancer Epidemiol Biomarkers | USA | 140/74  Orthopedic controls | GG,GA,AA  75,26,2 | GG,GA,AA  61,12,1 | 0.64 | Additive  dominant | 0.33  0.14 |
|  |  |  | rs2282140 | Intron variant | Savage 2007[32] | Cancer Epidemiol Biomarkers | USA | 140/74  Orthopedic controls | CC,CT,TT  78,17,2 | CC,CT,TT  47,21,1 | 0.4 | Additive  dominant | 0.15  0.07 |
|  |  |  | rs1803989 | 500B downstream variant, coding sequence variant, downstream transcript variant, synonymous variant | Savage 2007[32] | Cancer Epidemiol Biomarkers | USA | 140/74  Orthopedic controls | TT,TC,CC  97,6,0 | TT,TC,CC  63,11,0 | 0.5 | Additive  dominant | 0.04  0.04 |
|  | IL1B | 2 | rs1143627 | 5 prime UTR variant,genic upstream transcript variant, upstream transcript variant | He 2014[33] | Int Orthop. | China | 120/120 | TT,TC,CC  60,50,10 | TT,TC,CC  50,49,21 | 0.15 | Tc vs TT  CC vs TT  C vs T | 0.56  0.03  0.04 |
|  |  |  | rs16944 | Upstream transcript variant, 2KB upstream variant | He 2014[33] | Int Orthop. | China | 120/120 | CC,CT,TT  59,52,9 | CC,CT,TT  52,48,20 | 0.13 | CT vs CC  TT vs CC  T vs C | 0.87  0.04  0.08 |
|  |  |  | rs1143634 | Synonymous variant, coding sequence variant | He 2014[33] | Int Orthop. | China | 120/120 | CC,CT,TT  77,30,13 | CC,CT,TT  79,32,9 | 0.04 | CT vs CC  TT vs CC  T vs C | 0.9  0.4  0.51 |
|  | IL6 | 7 | rs1800796 | Non-coding transcript variant,upstream transcript variant, intron variant,  genic upstream transcript variant | Shang 2017[34] | Biomedical Research | China | 191/207 | CC,CG,CG  85,88,17 | CC,CG,CG  124,68,15 | 0.2 | CG vs CC  GG vs CC  CG+GG vs CC  G vs CC | 0.003  0.19  0.002  0.001 |
|  |  |  | rs1800795 | Upstream transcript variant, intron variant, genic upstream transcript variant | Qi 2016[35] | Tumour Biol. | China | 206/206 | GG,GC,CC  94,83,39 | GG,GC,CC  130,62,24 | 0.0003 | GC vs GG  CC vs GG  GC+CC vs GG | 0.028  0.022  0.029 |
|  |  |  |  |  | Oliveira 2007[36] | J Pediatr Hematol Oncol  . | Brazil | 64/160 | GG,GC,CC  9,23,32 | GG,GC,CC  10,68,82 | 0.4 | - | - |
|  |  |  | rs1800796 | non_coding transcript variant, upstream transcript variant, intron variant, genic upstream transcript variant | Qi 2016[35] | Tumour Biol. | China | 206/206 | GG,GC,CC  94,99,23 | GG,GC,CC  95,89,32 | 0.14 | GC vs GG  CC vs GG  GC+CC vs GG  C vs G | 0.289  0.189  0.193  0.172 |
|  | IL8 | 4 | rs4073 | 2KB upstream variant, upstream transcript variant | Tian 2016[37] | Int J Clin Exp Med. | China | 109/109 | TT,TA,AA  58,32,19 | TT,TA,AA  73,24,12 | 0.0004 | TA vs TT  AA vs TT | 0.014  0.025 |
|  |  |  |  |  | Chen 2016[38] | Tumour Biol  . | China | 190/190 | TT,TA,AA  99,63,28 | TT,TA,AA  122,50,18 | 0.001 | TA vs TT  AA vs TT | 0.021  0.018 |
|  |  |  | rs2227306 | 2KB upstream variant, upstream transcript variant | Chen 2016[38] | Tumour Biol  . | China | 190/190 | CC,CT,TT  86,90,14 | CC,CT,TT  90,88,12 | 0.11 | CT vs CC  TT vs CC  CT+TT vs CC  T vs C | 0.271  0.262  0.159  0.105 |
|  | IL10 | 1 | rs1800896 | Genic upstream transcript variant ,intron variant, 2KB upstream variant upstream transcript variant | Cui 2016[39] | Tumour Biol  . | China | 260/260 | AA,AG,GG  139,73,48 | AA,AG,GG  155,76,29 | >0.05 | AG vs AA  GG vs AA  AG+GG vs AA  G vs A | 0.017  0.01  0.019  0.026 |
|  |  |  |  |  | Oliveira 2007[36] | J Pediatr Hematol Oncol  . | Brazil | 80/157 | AA,AG,GG  5,44,29 | AA,AG,GG  23,76,58 | 0.8 | - | - |
|  |  |  | rs1800871 | Genic upstream transcript variant,intron variant, 2KB upstream variant ,upstream transcript variant | Cui 2016[39] | Tumour Biol  . | China | 260/260 | CC,CT,TT  106,120,34 | CC,CT,T  99,118,43 | 0.4 | CT vs CC  TT vs CC  CT+TT vs CC  T vs C | 0.159  0.137  0.161  0.251 |
|  |  |  | rs1800872 | Genic upstream transcript variant,intron variant, 2KB upstream variant ,upstream transcript variant | Cui 2016[39] | Tumour Biol  . | China | 260/260 | AA, AC,CC  108,125,27 | AA, AC,CC  100,128,32 | 0.4 | AC vs AA  CC vs AA  AC+CC vs AA  C vs A | 0.195  0.152  0.204  0.195 |
|  | IL 12A | 3 | rs568408 | 3 prime UTR variant, intron variant | Wang 2013[40] | DNA Cell Biol  . | China | 106/210 | GG,GA,AA  66,37,3 | GG,GA,AA  156,47,7 | 0.15 | GA vs GG  AA vs GG  GA+AA vs GG | <0.05  >0.05  <0.05 |
|  |  |  | rs2243115 | Intron variant, 2KB_upstream variant ,upstream transcript variant | Wang 2013[40] | DNA Cell Biol  . | China | 106/210 | TT,TG,GG  89,16,1 | TT,TG,GG  175,31,4 | 0.07 | TG vs GG  TT vs GG  TG+TT vs GG | >0.05  >0.05  >0.05 |
|  |  |  | rs3212227 | 3 prime UTR variant | Wang 2013[40] | DNA Cell Biol | China | 106/210 | AA,AC,CC  27,50,29 | AA,AC,CC  78,101,31 | 0.9 | AC vs AA  CC vs AA  AC+CC vs AA | >0.05  <0.05  <0.05 |
|  | IL16 | 15 | rs4778889 | Intron variant, genic upstream transcript variant ,upstream transcript variant | Tang 2016[41] | Sci Rep | China | 358/402 | TT,TC,CC  215,127,16 | TT,TC,CC  240,140,22 | 0.8 | TC vs TT  CC vs TT  C vs T | 0.94  0.55  0.76 |
|  |  |  | rs11556218 | Coding sequence variant, non-coding transcript variant, missense variant | Tang 2016[41] | Sci Rep | China | 358/402 | TT,TG,GG  165,174,19 | TT,TG,GG  235,151,16 | 0.17 | TG vs TT  GG vs GG  G vs T | 0.001  0.14  0.002 |
|  |  |  | rs4072111 | Coding sequence variant,missense variant ,5 prime UTR variant, genic upstream transcript variant, non-coding | Tang 2016[41] | Sci Rep | China | 358/402 | CC,CT,TT  229,158,15 | CC,CT,TT  218,124,16 | 0.8 | CT vs CC  TT vs CC  T vs C | 0.19  0.75  0.45 |
|  | IL27 | 16 | rs153109 | Intron variant, upstream transcript variant, genic upstream transcript variant | Tang 2014[42] | Medicine | China | 160/250 | AA,AG,GGG  56,85,19 | AA,AG,GGG  100,124,26 | 0.17 | AG vs AA  GG vs AA  G vs A | 0.355  0.44  0.347 |
|  |  |  | rs17855750 | Missense variant, coding sequence variant | Tang 2014[42] | Medicine | China | 160/250 | TT,TG,GG 132,28,0 | TT,TG,GG  205,45,0 | 0.12 | TG vs TT  GG vs TT  G vs T | 0.897  …  0.902 |
|  |  |  | rs181206 | Missense variant, coding sequence variant | Tang 2014[42] | Medicine | China | 160/250 | TT,TC,CC  131,29,0 | TT,TC,CC  207,43,0 | 0.14 | TC vs TT  CC vs TT  C vs T | 0.81  …  0.819 |
|  | ITGA3 | 17 | rs2230392 | Missense variant, non-coding transcript variant, coding sequence variant | Yang 2014[43] | Med Oncol | China | 118/126 | AA,AG,GG  50,44,24 | AA,AG,GG  33,54,39 | > 0.05 | AA  AG  GG  G vs A | 0.02  0.003 |
|  |  |  | rs2285524 | Synonymous variant, non-coding transcript variant, coding sequence variant | Yang 2014[43] | Med Oncol | China | 118/126 | AA,AG,GG  36,47,35 | AA,AG,GG  31,57,38 | > 0.05 | AA  AG  GG  G vs A | 0.55  0.48 |
|  |  |  | rs16948627 | Intron variant | Yang 2014[43] | Med Oncol | China | 118/126 | AA,AC,CC  37,48,33 | AA,AC,CC  47,24,55 | < 0.05 | AA  AC  CC  C vs A | 0.001  0.28 |
|  | LOX | 5 | -22 G/C |  | Liu 2012[44] | PLoS One | China | 326/433 | GG,GC,CC  306,9,11 | GG,GC,CC  425,5,3 | >0.05 | CG vs GG  CC vs GG  C vs G | 0.093*  0.006  4.18×10^−5^ |
|  |  |  | 225C/G |  | Liu 2012[44] | PLoS One | China | 326/433 | CC,CG,GG  315,11,0 | CC,CG,GG  416,17,0 | >0.05 | CG vs CC  G vs C | 0.69  0.693 |
|  |  |  | rs1800449 | Coding sequence variant, genic upstream transcript variant, missense variant ,upstream transcript variant | Liu 2012[44] | PLoS One | China | 326/433 | GG,GA,AA  209,86,31 | GG,GA,AA  301,112,20 | >0.05 | GA vs GG  AA vs GG  A vs G | 0.552  0.006  0.013 |
|  | MDM2 | 12 | rs1690916 | 3 prime UTR variant | Bilbao-Aldaiturriaga 2016[45] | Pediatr Res | Spain | 99/125 | GG,GA,AA  38,49,12 | GG,GA,AA  73,74,20 | >0.05 | GG  GA  AA | 0.68 |
|  |  |  |  |  | Bilbao-Aldaiturriaga 2016[45] | Pediatr Res | Slovenia | 27/91 | GG,GA,AA  3,17,7 | GG,GA,AA  26,51,4 | >0.05 | GG  GA  AA | 0.11 |
|  |  |  |  |  | Naumov 2012[46] | Bull Exp Biol Med | Russia | 24/86 | GG,GA,AA  12,9,3 | GG,GA,AA  23,45,18 | >0.05 | GG  GA  AA | 0.1 |
|  |  |  |  |  | Mirabello 2011[47] | BMC Cancer | USA | 96/1416 | GG,GA,AA  44,44,8 | GG,GA,AA  468,691,257 | 0.94 | GG  GA  AA | 0.006 |
|  |  |  | rs2279744 | Intron variant, genic upstream transcript variant, upstream transcript variant | Bilbao-Aldaiturriaga 2016[45] | Pediatr Res | Spain | 99/125 | TT,TG,GG  44,37,13 | TT,TG,GG  65,72,27 | >0.05 | TT  TG  GG | 0.53 |
|  |  |  |  |  | Bilbao-Aldaiturriaga 2016[45] | Pediatr Res | Slovenia | 27/91 | TT,TG,GG  11,14,1 | TT,TG,GG  40,38,14 | >0.05 | TT  TG  GG | 0.19 |
|  |  |  |  |  | Mirabello 2011[47] | BMC Cancer | USA | 96/1416 | TT,TG,GG  32,46,17 | TT,TG,GG  580,662,174 | >0.05 | TT  TG  GG | 0.20 |
|  |  |  |  |  | Toffoli 2009[48] | Clin Cancer Res  . | Italy | 201/250 | TT,TG,GG  69,86,46 | TT,TG,GG  111,107,32 | >0.05 | TT  TG  GG | 0.52 |
|  |  |  |  |  | Hattinger 2016[4] | Oncotarget. | Italy | 196/470 | TT,TG,GG  67,82,47 | TT,TG,GG  221,193,56 | 0.2 | TT  TG  GG | < 0.001 |
|  | MMP2 | 16 | rs243865 | Upstream transcript variant, 2KB upstream variant | Cui 2016[49] | Biomarkers | China | 251/251 | CC,CT,TT  148,72,31 | CC,CT,TT  179,52,20 | >0.05 | CT vs CC  TT vs CC  CT+TT vs CC  T vs C | 0.014  0.028  0.032  0.018 |
|  | MMP3 | 11 | rs3025058 | Upstream transcript variant ,2KB upstream variant | Cui 2016[49] | Biomarkers | China | 251/251 | 6A/6A,6A/5A  ,5A/5A  76,126,,49 | 6A/6A,6A/5A  ,5A/5A  95,104,52 | >0.05 | 6A/5A  5A/5A  6A/5A + 5A/5A  6A vs 5A | 0.131  0.228  0.109  0.091 |
|  | MMP9 | 20 | rs3918242 | 2KB upstream variant, Upstream transcript variant | Cui 2016[49] | Biomarkers | China | 251/251 | CC,CT,TT  79,128,45 | CC,CT,TT  97,102,52 | >0.05 | CT vs CC  TT vs CC  CT+TT vs CC  T vs C | 0.2  0.18  0.5  0.07 |
|  | MTAP | 9 | rs7023329 | Intron variant | Zhi 2016[50] | J Cancer | China | Discovery stage 284/981 | AA,AG,GG  90,42,52 | AA,AG,GG  238,492,251 | >0.05 | AA  AG  GG  G vs A | *0.05*  0.05 |
|  |  |  |  | Intron variant | Zhi 2016[50] | J Cancer | China | Validation Stage  108/597 | AA,AG,GG  37,51,20 | AA,AG,GG  135,301,161 | >0.05 | AA  AG  GG  G vs A | 0.049  0.047 |
|  |  |  | rs7027989 | Intron variant | Zhi 2016[50] | J Cancer | China | 108/597 | GG,GA,AA  65,38,5 | GG,GA,AA  405,38,5 | >0.05 | AA  AG  GG  G vs A | 0.2914  0.1054 |
|  | NAT2 | 8 | rs1799929 | Coding sequence variant synonymous variant | Huang 2015[51] | Onco Targets Ther  . | China | 260/280 | CC,CT,TT  236,20,4 | CC,CT,TT  254,30,2 | 0.295 | CT vs CC  TT vs CC  CT+TT vs CC | 0.272  0.379  0.452 |
|  |  |  | rs1208 | Missense variant, coding sequence variant | Huang 2015[51] | Onco Targets Ther  . | China | 260/280 | AA,AG,GG  248,9,3 | AA,AG,GG  272,13,1 | 0.063 | AG vs AA  GG vs AA  AG+GG | 0.534  0.304  0.878 |
|  |  |  | rs1041983 | Coding sequence variant synonymous variant | Huang 2015[51] | Onco Targets Ther  . | China | 260/280 | CC,CT,TT  120,107,33 | CC,CT,TT  109,145,32 | 0.115 | CT vs CC  TT vs CC  CT+TT vs CC | 0.029  0.816  0.057 |
|  |  |  | rs1801280 | Missense variant, coding sequence variant | Huang 2015[51] | Onco Targets Ther  . | China | 260/280 | TT,TC,CC  238,20,2 | TT,TC,CC  247,36,3 | 0.205 | TC vs CC  TT vs CC  TC+TT vs CC | 0.06  0.688  0.057 |
|  |  |  | rs1799930 | Missense variant, coding sequence variant | Huang 2015[51] | Onco Targets Ther  . | China | 260/280 | GG,GA,AA  158,73,29 | GG,GA,AA  167,98,21 | 0.216 | GA vs GG  AA vs GG  AA+GA vs GG | 0.209  0.218  0.572 |
|  |  |  | rs1799931 | Missense variant, coding sequence variant | Huang 2015[51] | Onco Targets Ther  . | China | 260/280 | GG,GA,AA 177,77,6 | GG,GA,AA  164,112,10 | 0.081 | GA vs GG  AA vs GG  AA+GA vs GG | 0.014  0.266  0.01 |
|  |  |  | rs1801279 | Missense variant, coding sequence variant | Huang 2015[51] | Onco Targets Ther  . | China | 260/280 | GG,GA,AA 251,8,1 | GG,GA,AA 270,15,1 | 0.125 | GA vs GG  AA vs GG  AA+GA vs GG | 0.213  0.959  0.238 |
|  | NBN | 8 | rs709816 | Missense variant, coding sequence variant, synonymous varianr | Jin 2015[16] | Pak J Med Sci. | China | 148/298 | GG,GC+CC  55,93 | GG,GC+CC  115,183 | >0.05 | GC+CC vs GG | 0.78 |
|  |  |  |  |  | Goricar 2015[52] | J Med Biochem. | Slovenia | 79/373 | CC,CT+TT  29,50 | CC,CT+TT  139,234 |  | CT+TT vs CC | 0.926 |
|  |  |  | rs1805794 | Missense variant, 5 prime UTR variant, coding sequence variant, intron variant | Jin 2015[16] | Pak J Med Sci. | China | 148/298 | GG,GC+CC  52,96 | GG,GC+CC  127,171 | >0.05 | GC+CC vs GG | <0.001 |
|  |  |  |  |  | Goricar 2015[52] | J Med Biochem. | Slovenia | 79/373 | GG,GC+CC  11,68 | GG,GC+CC  33,340 |  | GC+CC vs GG | 0.17 |
|  |  |  | rs1063054 | 3 prime UTR variant, genic downstream transcript variant | Goricar 2015[52] | J Med Biochem. | Slovenia | 79/373 | AA,AC+CC  38,38 | AA,AC+C  163,210 |  | AC+CC vs AA | 0.32 |
|  | PRKCG | 19 | rs454006 | Intron variant | Zhang 2014[53] | Tumour Biol | China | 610/1610 | CC,CT,TT  90,215,305 | CC,CT,TT  52,213,345 | VEGF | CC  CT  TT | 0.001 |
|  |  |  |  |  | Lu 2015[54] | Tumour Biol | China | 388/388 | CC,CT,TT  98,123,167 | CC,CT,TT  53,162,173 | >0.05 | TC+CC vs. TT  CC vs. TT+TC  CC vs. TT  C vs T | 0.66  0.001  0.001 0.008 |
|  |  |  | rs2242245 | Intron variant | Zhang 2014[53] | Tumour Biol | China | 610/1610 | GG,GA,AA  27,168,415 | GG,GA,AA  20,163,427 | 0.4 | AA  AG  GG | 0.52 |
|  |  |  |  |  | Lu 2015[54] | Tumour Biol | China | 388/388 | TT,TC,CC  285,84,19 | TT,TC,CC  282,93,13 | >0.05 | TC+CC vs. TT  CC vs. TT+TC  CC vs. TT  C vs T | 0.8  0.3  0,3  0.8 |
|  |  |  | rs8103851 | Intron variant | Zhang 2014[53] | Tumour Biol | China | 610/1610 | GG,GC,CC  113,286,211 | GG,GC,CC  137,278,195 | 0.05 | CC  CG  GG | 0.2 |
|  |  |  |  |  | Lu 2015[54] | Tumour Biol | China | 388/388 | GG,GC,CC  87,169,132 | GG,GC,C  81,172,135 | >0.05 | GG+GC vs. CC  GG vs. CC+CG  GG vs. CC  G vs C | 0.8  0.6  0.6  0.6 |
|  |  |  | rs2547362 | Coding sequence variant, synonymous variant | Lu 2015[54] | Tumour Biol | China | 388/388 | TT,TC,CC  283,81.24 | TT,TC,CC  287,88,13 | >0.05 | TC+CC vs. TT  CC vs. TT+TC  CC vs. TT  C vs T | 0.7  0.06  0.07  0.3 |
|  |  |  | rs3745406 | Missense variant, coding sequence variant, synonymous variant | Lu 2015[54] | Tumour Biol | China | 388/388 | TT,TC,CC  120,196,72 | TT,TC,CC  149,176,53 | >0.05 | TC+CC vs. TT  CC vs. TT+TC  CC vs. TT  C vs T | 0.014  0.09  0.016  0.009 |
|  | RECQL5 | 17 | rs820196 | Non coding transcript variant, coding sequence variant, missense variant, 5 prime UTR variant, genic downstream variant | Zhi 2014[55] | Tumour Biol | China | 212/240 | CC,CT,TT  20,122,98 | CC,CT,TT  37,109,66 | 0.48 | CC  CT  TT | 0.005 |
|  |  |  |  |  | Dong 2015[56] | Genet Mol Res. | China | 185/201 | CC,CT,TT  30,96,59 | CC,CT,TT  18,102,81 | >0.05 | CC  CT  TT  C vs T | 0.005  0.001 |
|  |  |  | rs820200 | Intron variant, genic upstream transcript variant , genic downstream transcript variant, 3 prime UTR variant | Zhi 2014[55] | Tumour Biol | China | 212/240 | GG,GT,TT | GG,GT,TT  39,132,69 | 0.07 | GG  GT  TT | 0.59 |
|  |  |  | rs4789223 | Intron variant, genic upstream transcript variant, genic downstream transcript variant, 2KB upstream variant, upstream transcript variant | Zhi 2014[55] | Tumour Biol | China | 212/240 | AA,AG,GG  46,128,38 | AA,AG,GG  34,114,92 | 0.9 | AA  AG  GG  A vs G | <0.001  <0.001 |
|  | TGF-β1 | 19 | rs1800470 | Missense variant, coding sequence variant | Wu 2015[57] | Tumour Biol | China | 124/136 | TT,TC,CC  52,45,27 | TT,TC,CC  37,59,40 | >0.5 | TT  TC  CC  T vs C | 0.04  0.001 |
|  |  |  |  |  | Xu 2014[58] | DNA Cell Biol  . | China | 202/216 | TT,TC,CC  42,102,58 | TT,TC,CC  66,110,40 | 0.6 | TC vs TT  CC vsTT | 0.11  0.001 |
|  |  |  | rs180xc0469 | Downstream transcript variant ,500B downstream variant, 2KB upstream variant ,upstream transcript variant | Wu 2015[57] | Tumour Biol | China | 124/136 | CC,CT,TT  31,65,40 | CC,CT,TT  36,47,41 | >0.5 | CC  CT  TT  C vs T | 0.26  0.77 |
|  |  |  |  |  | Xu 2014[58] | DNA Cell Biol  . | China | 202/216 | CC,CT,TT  60,104,38 | CC,CT,TT  69,109,38 | 0.62 | CT vs CC  TT vs CC | 0.678  0.629 |
|  |  |  | rs1982073 | Missense variant ,coding sequence variant | Wu 2015[57] | Tumour Biol | China | 124/136 | TT,TC,CC  39,50,35 | TT,TC,CC  38,56,42 | >0.5 | TT  TC  CC  T vs C | 0.8  0.48 |
|  | TNF-α | 6 | rs1800629 | 2KB upstream variant, upstream transcript varia | Zhao 2015[59] | Int J Clin Exp Pathol. | China | 80/99 | GG,GA,AA  30,34,16 | GG,GA,AA  45,48,6 | 0.14 | GA vs GG  AA vs GG  G vs A | 0.9  0.007  0.031 |
|  |  |  |  |  | Oliveira 2007[36] | J Pediatr Hematol Oncol. | Brazil | 80/160 | GG,GA,AA  55,19,6 | GG,GA,AA  130,25,5 |  | - | - |
|  |  |  |  |  | Patino-Garcia 2000[60] | J Medical Genetics | Spain | 63/111 | GG,GA,AA  49,14,0 | GG,GA,AA  83,28,0 | 0.12 | - | - |
|  |  |  | rs361525 | 2KB upstream variant, upstream transcript variant | Zhao 2015[59] | Int J Clin Exp Pathol. | China | 80/99 | GG,GA,AA  62,16,2 | GG,GA,AA  74,24,1 | 0.14 | GA vs GG  AA vs GG  G vs A | 0.4  0.5  0.03 |
|  |  |  |  |  | Patino-Garcia 2000[60] | J Medical Genetics | Spain | 63/111 | GG,GA,AA  61,2,0 | GG,GA,AA  93,18,0 | 0.4 | - | - |
|  | TNF-β | 6 | rs909253 | Intron variant | Oliveira 2007[36] | J Pediatr Hematol Oncol. | Brazil | 80/160 | GG,GA,AA  34,37,9 | GG,GA,AA  77,66,17 | 0.6 |  |  |
|  | TP53 | 17 | rs17878362 | TP53_PIN3_IVS3 + 16 bp  (STR) | Hattinger 2016[4] | Oncotarget. | Italy | 196/470 | WT,HET,VAR 133,49,14 | WT,HET,VAR 324,127,19 |  | WT  HET  VAR | >0.05 |
|  |  |  | rs8079544 | Upstream transcript variant, intron variant, genic upstream transcript variant | Savage 2007[61] | Pediatr Blood Cancer | USA | 103/73 | GG,GA,AA  97,6,0 | GG,GA,AA  66,7,0 | >0.05 | Additive  Dominant  Recessive | 0.35  0.35  no homozygote variants. |
|  |  |  | rs1642785 | Upstream transcript variant, intron variant, genic upstream transcript variant, 5 prime UTR variant | Savage 2007[61] | Pediatr Blood Cancer | USA | 99/68 | CC,CG,GG  47,43,9 | CC,CG,GG  31,36,1 | >0.05 | Additive  Dominant  Recessive | 0.95  0.81  0.041 |
|  |  |  |  |  | Hattinger 2016[4] | Oncotarget. | Italy | 196/470 | CC,CG,GG  16,64,116 | CC,CG,GG  263,169,38 |  | HET + VAR | < 0.0001 |
|  |  |  | rs1042522 | Missense variant, upstream transcript variant, genic upstream transcript variant, coding sequence variant | Savage 2007[61] | Pediatr Blood Cancer | USA | 98/67 | CC,CG,GG  47,41,10 | CC,CG,GG  30,36,1 | >0.05 | Additive  Dominant  Recessive | 0.054  0.069  0.028 |
|  |  |  |  |  | Ru 2015[62] | Int J Clin Exp Pathol | china | 210/420 | CC,CG,GG  59,106,44 | CC,CG,GG  162,194,64 | 0.64 | CC  CG  GG | 0.007 |
|  |  |  |  |  | Hattinger 2016[4] | Oncotarget. | Italy | 196/470 | CC,CG,GG  139,41,16 | CC,CG,GG  277,160,33 | >0.05 | Wild type | 0.005 |
|  |  |  |  |  | Toffoli 2009[48] | Clin Cancer Res  . | Italy | 201/250 | CC,CG,GG  16,43,142 | CC,CG,GG  17,87,146 | >0.05 | CG vs GG  CC vs GG | 0.002  1 |
|  |  |  | rs9895829 | Upstream transcript variant, intron variant, genic upstream transcript variant | Savage 2007[61] | Pediatr Blood Cancer | USA | 101/71 | TT,TC,CC  94,7,0 | TT,TC,CC  64,7,0 | >0.05 | Additive  Dominant  Recessive | 0.49  0.49  NH |
|  |  |  | rs2909430 | Upstream transcript variant, intron variant, genic upstream transcript variant | Savage 2007[61] | Pediatr Blood Cancer | USA | 95/66 | AA,AG,GG  65,27,3 | AA,AG,GG  45,21,0 | >0.05 | Additive  Dominant  Recessive | 0.33  0.97  0.15 |
|  |  |  | rs1800372 | Synonymous variant ,coding sequence variant | Savage 2007[61] | Pediatr Blood Cancer | USA | 97/65 | AA,AG,GG  94,3,0 | AA,AG,GG  60,5,0 | >0.05 | Additive  Dominant  Recessive | 0.19  0.19  NH |
|  |  |  | rs1625895 | Intron variant | Savage 2007[61] | Pediatr Blood Cancer | USA | 98/66 | AA,AG,GG  66,30,2 | AA,AG,GG  43,22,1 | >0.05 | Additive  Dominant  Recessive | 0.91  0.77  0.81 |
|  |  |  | E3346_28# |  | Savage 2007[61] | Pediatr Blood Cancer | USA | 99/68 | GG,GC,CC  97,2,0 | GG,GC,CC  63,5,0 | >0.05 | Additive  Dominant  Recessive | 0.09  0.09  NH |
|  |  |  | rs12947788 | Intron variant | Savage 2007[61] | Pediatr Blood Cancer | USA | 102/72 | CC,CT,TT  84,16,2 | CC,CT,TT  62,10,0 | >0.05 | Additive  Dominant  Recessive | 0.46  0.51  0.23 |
|  |  |  | rrs1614984 | 500B downstream variant ,downstream transcript variant | Savage 2007[61] | Pediatr Blood Cancer | USA | 98/66 | CC,CT,TT  34,53,11 | CC,CT,TT  28,29,9 | >0.05 | Additive  Dominant  Recessive | 0.44  0.32  0.64 |
|  |  |  | rs9894946 |  | Savage 2007[61] | Pediatr Blood Cancer | USA | 96/66 | TT,TC,CC  65,28,3 | TT,TC,CC  40,25,1 | >0.05 | Additive  Dominant  Recessive | 0.44  0.35  0.52 |
|  |  |  | E3355_424# |  | Savage 2007[61] | Pediatr Blood Cancer | USA | 103/72 | AA,AG,GG  87,16,0 | AA,AG,GG  62,10,,0 | >0.05 | Additive  Dominant  Recessive | 0.76  0.76  NH |
|  |  |  | rs12951053 | Intron variant | Ru 2015[62] | Int J Clin Exp Pathol | China | 210/420 | AA,AC,CC  54,103,53 | AA,AC,CC  140,198,82 | >0.05 | AA  AC  CC | 0.03 |
|  |  |  | rs8064946 | 2KB upstream variant,upstream transcript variant, intron variant, genic upstream transcript variant | Ru 2015[62] | Int J Clin Exp Pathol | China | 210/420 | GG,GC,CC  68,100,41 | GG,GC,CC  135,189,96 | >0.05 | GG  GC  CC | 0.56 |
|  |  |  | rs12602273 | Genic upstream transcript variant, intron variant | Ru 2015[62] | Int J Clin Exp Pathol | China | 210/420 | CC,CG,GG | CC,CG,GG | >0.05 | CC  CG  GG | 0.15 |
|  | TP53/VMP1 | 17 | rs1295925 | Intron variant | Zhang 2018[63] | Cancer Biol Ther. | China | 1300/1300 | TT,TC,CC  476,583,241 | TT,TC,CC  399,631,241 | >0.05 | TC vs TT  CC vs TT  C vs T | 0.004  0.009  0.003 |
|  | TP53/BCAS1 | 20 | rs3787547 | Intron variant ,genic upstream transcript ariant | Zhang 2018[63] | Cancer Biol Ther. | China | 1300/1300 | GG,AG,AA  654,513,133 | GG,AG,AA  570,566,163 | >0.05 | AG vs GG  AA vs GG  A vs G | 0.003  0.006  4.0 × 10^−4^ |
|  | TP53/BCAS1 | 20 | rs290392 | 2KB upstream variant, upstream transcript variant | Zhang 2018[63] | Cancer Biol Ther. | China | 500/500 | AA,AG,GG  164,259,77 | AA,AG,GG  176,241,83 | >0.05 | AG vs AA  GG vs AA | 0.31  0.982 |
|  | VEGF | 6 | rs699947 | Intron variant, genic downstream transcript variant,2KB upstream variant, upstream transcript variant | Zhang 2015[64] | Genet Mol Res. | China | 182/182 | CC,CA,AA  68,79,35 | CC,CA,AA  88,71,23 | 0.16 | CA vs CC  AA vs CC  CA+AA vs CC | 0.11  0.03  0.03 |
|  |  |  |  |  | Zhao 2015[65] | Pak J Med Sci. | China | 176/176 | CC,CA,AA  62,78,36 | CC,CA,AA  84,71,21 | >0.05 | CA vs CC  AA vs CC  CA+AA vs CC | 0.09  <0.05  <0.05 |
|  |  |  |  |  | Tie 2014[66] | Int J Clin Exp Pathol  . | China | 165/330 | CC,CA,AA  64,72,29 | CC,CA,AA  159,136,35 | 0.46 | CA vs CC  AA vs CC  A vs C | 0.19  0.001  0.008 |
|  |  |  |  |  | Liu 2015[67] | Oncol Lett  . | China | 186/186 | CC,CA,AA  79,75,107 | CC,CA,AA  87,73,26 | >0.05 | CA vs CC  AA vs CC | 0.59  0.32 |
|  |  |  | rs3025039 | 3 prime UTR variant ,intron variant,  genic downstream transcript variant | Zhang 2015[64] | Genet Mol Res. | China | 182/182 | CC,CT,TT  128,35,19 | CC,CT,TT  138,32,12 | <0.05 | CT vs CC  TT vs CC  CT+TT vs CC | 0.55  0.17  0.24 |
|  |  |  |  |  | Zhao 2015[65] | Pak J Med Sci. | China | 176/176 | CC,CT,TT  85,75,16 | CC,CT,TT  92,71,13 | >0.05 | CT vs CC  TT vs CC  CT+TT vs CC | 0.55  0.41  0.46 |
|  |  |  |  |  | Wang 2013[68] | Tumour Biol. | China | 330/342 | CC,CT,TT  185,116,29 | CC,CT,TT  207,123,12 | >0.05 | CT vs CC  TT vs CC  CT+TT vs CC | 0.74  0.005  0.04 |
|  |  |  |  |  | Tie 2014[66] | Int J Clin Exp Pathol  . | China | 165/330 | CC,CT,TT  111,39,15 | CC,CT,TT  232,74,24 | < 0.001 | CT vs CC  TT vs CC  CT+TT vs CC | 0.67  0.44  0.33 |
|  |  |  |  |  | Zhang 2015[69] | Genet Mol Res | China | 180/360 | CC,CT,TT  66,92,22 | CC,CT,TT  148,175,37 | 0.16 | CT vs CC  TT vs CC  T vs C | 0.4  0.35  0.3 |
|  |  |  |  |  | Liu 2015[67] | Oncol Lett. | China | 186/186 | CC,CT,TT  125,46,16 | CC,CT,TT  134,42,10 | >0.05 | CT vs CC  TT vs CC | 0.52  0.2 |
|  |  |  |  |  | Li 2017[70] | Biomedical Research | China | 242/253 | CC,CT,TT  131,95,16 | CC,CT,TT  159,86,8 | 0.4 | CT vs CC  TT vs CC  CT+TT vs CC  C vs T | 0.12  0.004  0.04  0.02 |
|  |  |  |  |  | Cao 2016[71] | Int J Clin Exp Pathol | China | 322/343 | CC,CT,TT  188,103,31 | CC,CT,TT  213,108,22 | 0.1 | CT vs CC  TT vs CC | 0.33  0.13 |
|  |  |  |  |  | Hu 2015[72] | Genet Mol Res. | China | 130/130 | CC,CT,TT  67,47,16 | CC,CT,TT  79,44,7 | 0.79 | CT vs CC  TT vs CC  CT+TT vs CC | 0.39  0.03  0.13 |
|  |  |  | rs833061 | Intron variant ,genic downstream transcript variant,2KBupstream variant ,upstream transcript variant | Zhang 2015[64] | Genet Mol Res. | China | 182/182 | TT,TC,CC  65,91,26 | TT,TC,CC  77,86,24 | 0.83 | TC vs TT  CC vs TT  CT+TC vs TT | 0.49  0.58  0.45 |
|  |  |  |  |  | Zhao 2015[65] | Pak J Med Sci. | China | 176/176 | TT,TC,CC  48,89,39 | TT,TC,CC  66,85,25 | >0.05 | TC vs TT  CC vs TT  CT+TC vs TT | 0.13  <0.05  <0.05 |
|  |  |  | rs1570360 | Intron variant ,genic downstream transcript variant,2KBupstream variant ,upstream transcript variant | Zhao 2015[65] | Pak J Med Sci. | China | 176/176 | AA,AG,GG  93,73,11 | AA,AG,GG  97,71,8 | >0.05 | AG vs AA  GG vs AA  GG+GA vs AA | 0.75  0.46  0.63 |
|  |  |  |  |  | Tie 2014[66] | Int J Clin Exp Pathol  . | China | 165/330 | AA,AG,GG  90,45,30 | AA,AG,GG  203,81,46 | < 0.001 | AG vs AA  GG vs AA | 0.32  0.15 |
|  |  |  |  |  | Liu 2015[67] | Oncol Lett. | China | 186/186 | AA,AG,GG  107,53,27 | AA,AG,GG  116,49,21 | <0.05 | AG vs AA  GG vs AA | 0.51  0.3 |
|  |  |  | rs2010963 | 5 prime UTR variant,genicupstream transcript variant, upstream transcript variant ,intron variant, genic downstream transcript variant | Zhao 2015[65] | Pak J Med Sci. | China | 176/176 | CC,CG,GG  61,85,30 | CC,CG,GG  67,81,28 | >0.05 | CG vs CC  GG vs CC | 0.55  0.61 |
|  |  |  |  |  | Wang 2013[68] | Tumour Biol. | China | 330/342 | CC,CG,GG  50,165,115 | CC,CG,GG  58.166,118 | 0.84 | CC vs. GG  GC vs. GG  CC/GC vs. GG  CC vs. GC/GG  C vs. G | 0.6  0.9  0.9  0.5 |
|  |  |  |  |  | Tie 2014[66] | Int J Clin Exp Pathol  . | China | 165/330 | CC,CG,GG  42,80,43 | CC,CG,GG  120,151,59 | 0.34 | CG vs CC  GG vs CC | 0.07  0.006 |
|  |  |  |  |  | Zhang 2015[64] | Genet Mol Res. | China | 182/182 | CC,CG,GG  48,90,42 | CC,CG,GG  138,170,53 | 0.95 | CG vs CC  GG vs CC | 0.05  0.002 |
|  |  |  |  |  | Liu 2015[67] | Oncol Lett. | China | 186/186 | CC,CG,GG  50,91,45 | CC,CG,GG  69,86,31 | >0.05 | CG vs CC  GG vs CC | 0.11  0.02 |
|  |  |  |  |  | Cao 2016[71] | Int J Clin Exp Pathol | China | 322/343 | CC,CG,GG  93,156,73, | CC,CG,GG  123,165,55 | 0.97 | CG vs CC  GG vs CC | 0.055  0.031 |
|  |  |  |  |  | Hu 2015[72] | Genet Mol Res. | China | 130/130 | CC,CG,GG  20,68,42 | CC,CG,GG  18,65,46 | 0.51 | CG vs CC  GG vs CC | 0.62  0.61 |
|  |  |  | rs10434 | 3 prime UTR variant ,intron variant, genic downstream transcript variant | Zhao 2015[65] | Pak J Med Sci. | China | 176/176 | GG,GA,AA  77,80,19 | GG,GA,AA  80,78,18 | 0.87 | GA vs GG  AA vs GG | 0.78  0.8 |
|  |  |  |  |  | Wang 2013[68] | Tumour Biol. | China | 330/342 | GG,GA,AA  95,157,78 | GG,GA,AA  97,172,73 | 0.84 | GA vs GG  AA vs GG | 0.62  0.7 |
|  |  |  |  |  | Tie 2014[66] | Int J Clin Exp Pathol  . | China | 165/330 | GG,GA,AA  68,76,20 | GG,GA,AA  151,146,33 | 0.79 | CT vs CC  TT vs Cc | 0.48  0.35 |
|  |  |  |  |  | Zhang 2015[64] | Genet Mol Res. | China | 182/182 | GG,GA,AA  77,80,23 | GG,GA,AA  163,155,42 | 0.58 | CT vs CC  TT vs Cc | 0.65  0.62 |
|  |  |  |  |  | Liu 2015[67] | Oncol Lett. | China | 186/186 | GG,GA,AA  75,86,25 | GG,GA,AA  84,83,19 | 0.82 | GA vs GG  AA vs GG | 0.5  - |
|  |  |  |  |  | Hu 2015[72] | Genet Mol Res. | China | 130/130 | GG,GA,AA  41,61,28 | GG,GA,AA  46,60,24 | 0.58 | GA vs GG  AA vs GG | 0.64  0.44 |
|  | XRCC1 | 19 | rs25487 | Missense variant, coding sequence variant | Hattinger 2016[4] | Oncotarget. | Italy | 196/470 | GG,GA,AA  82,86,28 | GG,GA,AA  207,211,52 | 0.9 | GG  GA  AA | >0.05 |
|  | XRCC3 | 14 | rs861539 | Genic downstream transcript variant, missense variant, intron variant, coding sequence variant | Jin 2015[16] | Pak J Med Sci. | China | 148/298 | CC,CT+TT  77,71 | CC,CT+TT  173,125 | - | CT vs CC  TT vs Cc | 0.17 |
|  |  |  |  |  | Hattinger 2016[4] | Oncotarget. | Italy | 196/470 | CC,CT,TT  63,100,33 | CC,CT,TT  179,207,84 |  | CC  CT  TT | >0.05 |
|  |  |  |  |  | Guo 2015[73] | Genet Mol Res. | China | 136/136 | CC,CT,TT  54,55,27 | CC,CT,TT  70,52,14 | 0.36 | CT vs CC  TT vs Cc | 0.23  0.01 |
|  |  |  |  |  | Yang 2015[74] | Int J Clin Exp Pathol. | China | 152/304 | CC,CT,TT  66,63,22 | CC,CT,TT  166,117,21 | 0.95 | CT vs CC  TT vs Cc | 0.15  0.003 |
|  |  |  |  |  | Goricar 2015[52] | J Med Biochem. | Slovenia | 79/373 | CC,CT+TT  39,40 | CC,CT+TT  153,220 |  | CT vs CC  TT vs Cc | 0.23 |
|  |  |  | rs1799794 | 5 prime UTR variant,downstream transcript variant, intron variant, 500B downstream variant | Goricar 2015[52] | J Med Biochem. | Slovenia | 79/373 | AA,AG+GG  47,31 | AA,AG+GG  247,126 |  | AG+GG vs AA | 0.31 |
|  | ABCB1 | 7 | rs1045642 | Synonymous variant, missense variant, coding sequence variant | Hattinger 2016[4] | Oncotarget. | Italy | 196/470 | TT,TC,CC  51,86,59 | TT,TC,CC  113,244,113 | 0.4 | TT  TC  CC | >0.05 |
|  |  |  | rs1128503 | Synonymous variant, coding sequence variant | Hattinger 2016[4] | Oncotarget. | Italy | 196/470 | CC,CT,TT  63,88,45 | CC,CT,TT  155,235,80 | 0.6 | CC  CT  TT | >0.05 |
|  |  |  | rs2032582 | missense variant, coding sequence variant | Hattinger 2016[4] | Oncotarget. | Italy | 196/470 | GG,GA,AA  57,94,45 | GG,GA,AA  132,249,89 | 0.14 | GG  GA  AA | >0.05 |
|  | ABCC2 | 10 | rs2273697 | Coding sequence variant, missense variant, non-coding transcript variant | Hattinger 2016[4] | Oncotarget. | Italy | 196/470 | GG,GA,AA  98,90,8 | GG,GA,AA  296,160,14 | 0.17 | GG  GA  AA | 0.011 |
|  |  |  | rs717620 | Genic upstream transcript variant, 5 prime UTR variant, non-coding transcript variant | Hattinger 2016[4] | Oncotarget. | Italy | 196/470 | GG,GA,AA 118,72,6 | GG,GA,AA  310,141,19 | 0.6 | GG  GA  AA | >0.05 |
|  |  |  | rs3740066 | Coding sequence variant, synonymous variant, genic downstream transcript variant, missense variant | Hattinger 2016[4] | Oncotarget. | Italy | 196/470 | GG,GA,AA  90,71,35 | GG,GA,AA  221,207,42 | 0.5 | GG  GA  AA | 0.030 |
|  | ABCG2 | 4 | rs2231137 | Missense variant, coding sequence nvariant | Hattinger 2016[4] | Oncotarget. | Italy | 196/470 | GG,GA,AA  165,31,0 | GG,GA,AA  414,52,4 | 0.11 | GG  GA  AA | >0.05 |
|  |  |  | rs2231142 | Missense variant, coding sequence variant | Hattinger 2016[4] | Oncotarget. | Italy | 196/470 | CC,CA,AA  167,25,4 | CC,CA,AA  376,89,5 | 0.9 | CC  CA  AA | >0.05 |
|  | RFC | 21 | rs1051266 | Genic upstream transcript variant, 5 prime UTR variant, missense variant, coding sequence variant | Hattinger 2016[4] | Oncotarget. | Italy | 196/470 | GG,GA,AA  77,88,31 | GG,GA,AA  174,230,66 | 0.5 | GG  GA  AA | >0.05 |
|  | hMLH1 | 3 | rs1799977 | Coding sequence variant missense variant, 5 prime UTR variant | Hattinger 2016[4] | Oncotarget. | Italy | 196/470 | AA,AG,GG  65,106,25 | AA,AG,GG  211,193,66 | 0.05 | AA  AG  GG | 0.010 |
|  | hMSH2 | 2 | rs2303428 | Intron variant | Hattinger 2016[4] | Oncotarget. | Italy | 196/470 | TT,TC,CC  167,27,2 | TT,TC,CC  404,61,5 | 0.13 | TT  TC  CC | >0.05 |
|  | hOGG1 | 3 | rs1052133 | Genic downstream transcript variant, 500B downstream variant, missense variant, non-coding transcript variant | Hattinger 2016[4] | Oncotarget. | Italy | 196/470 | CC,CG,GG 127,57,12 | CC,CG,GG  296,155,19 | 0.8 | CC  CG  GG | >0.05 |
|  | ERCC5/XPG | 13 | rs17655 | Missense variant, coding sequence variant | Hattinger 2016[4] | Oncotarget. | Italy | 196/470 | GG,GA,AA 112,61,23 | GG,GA,AA  254,193,23 | 0.07 | GG  GA  AA | 0.002 |
|  | DHFR | 5 | rs70991108 | Upstream transcript variant, intron variant, 2KB upstream variant  **(Ins)^** | Hattinger 2016[4] | Oncotarget. | Italy | 196/470 | Ins/Ins, Ins/del,Del/Del 67,98,31 | Ins/Ins, Ins/del,Del/Del  165,258,47 | <0.0001 | Ins/Ins  Ins/Del  Del/Del | >0.05 |
|  | FORLI | 11 | rs3833748 | Genic upstream transcript variant, upstream transcript variant, intron variant  (DELINS)# | Hattinger 2016[4] | Oncotarget. | Italy | 196/470 | Ins/Ins, Ins/del,Del/Del  188,8,0 | Ins/Ins, Ins/del,Del/Del  456,14,0 | 0.7 | Ins/Ins  Ins/Del  Del/Del | >0.05 |
|  | GGH | 8 | rs11545078 | Coding sequence variant, missense variant | Hattinger 2016[4] | Oncotarget. | Italy | 196/470 | CC,CT,TT  161,29,6 | CC,CT,TT  371,94,5 | 0.7 | CC  CT  TT | >0.05 |
|  |  |  | rs3758149 | 2KB upstream variant, upstream transcript variant | Hattinger 2016[4] | Oncotarget. | Italy | 196/470 | CC,CT,TT  104,61,31 | CC,CT,TT  244,188,38 | 0.042 | CC  CT  TT | 0.042 |
|  |  |  | rs1800909 | missense variant, coding sequence variant | Hattinger 2016[4] | Oncotarget. | Italy | 196/470 | TT,TC,CC  124,43,29 | TT,TC,CC  268,164,38 | 0.07 | TT  TC  CC | 0.011 |
|  | MTHFD1 | 14 | rs2236225 | missense variant, coding sequence variant | Hattinger 2016[4] | Oncotarget. | Italy | 196/470 | CC,CT,TT  53,104,39 | CC,CT,TT  132,235,103 | 0.9 | CC  CT  TT | >0.05 |
|  | MTHFR | 1 | rs1801133 | Non-coding transcript variant, missense variant, coding sequence variant | Hattinger 2016[4] | Oncotarget. | Italy | 196/470 | CC,CT,TT  59,96,41 | CC,CT,TT  150,259,61 | <0.001 | CC  CT  TT | >0.05 |
|  |  |  | rs1801131 | Non-coding transcript variant, missense variant, coding sequence variant | Hattinger 2016[4] | Oncotarget. | Italy | 196/470 | AA,AC,CC  92,86,18 | AA,AC,CC  193,221,56 | 0.6 | AA  AC  CC | >0.05 |
|  | SHMT | 17 | rs2273029 | Intron variant | Hattinger 2016[4] | Oncotarget. | Italy | 196/470 | CC,CT,TT  102,74,20 | CC,CT,TT  273,160,37 | 0.05 | CC  CT  TT | >0.05 |
|  | TYMS | 18 | rs34743033 | Genic upstream transcript variant, intron variant,5 prime UTR variant  **STR (3 repeats)** | Hattinger 2016[4] | Oncotarget. | Italy | 196/470 | WT,HET,VAR 86,77,33 | WT,HET,VAR 150,212,108 | 0.05 | WT  HET  VAR | 0.012 |
|  |  |  | rs16430 | 6 bp deletion in micro-RNA binding site**(DELINS)** | Hattinger 2016[4] | Oncotarget. | Italy | 196/470 | WT,HET,VAR 59,106,31 | WT,HET,VAR 174,221,75 | 0.7 | WT  HET  VAR | >0.05 |
|  | ATM | 11 | rs1800054 | 5 prime UTR variant,non-coding transcript variant,coding sequennce variant, missense variant | Hattinger 2016[4] | Oncotarget. | Italy | 196/470 | CC,CG,GG  192,4,0 | CC,CG,GG  451,19,0 | 0.7 | CC  CG  GG | >0.05 |
|  |  | 11 | rs1801516 | non-coding transcript variant,coding sequennce variant, missense variant | Hattinger 2016[4] | Oncotarget. | Italy | 196/470 | GG,GA,AA  141,49,6 | GG,GA,AA  362,103,5 | 0.4 | GG  GA  AA | >0.05 |
|  | CDKN1A/P21 | 6 | rs1801270 | coding sequennce variant, missense variant,geneic downstream transcript variant, synonymous variant | Hattinger 2016[4] | Oncotarget. | Italy | 196/470 | TT,TG,GG  165,29,2 | TT,TG,GG  400,66,4 | 0.5 | TT  TG  GG | >0.05 |
|  | CYP2C19 | 10 | rs4244285 | Coding sequence variant, synonymous | Hattinger 2016[4] | Oncotarget. | Italy | 196/470 | GG,GA,AA  145,45,6 | GG,GA,AA  343,118,9 | 0.8 | GG  GA  AA | >0.05 |
|  | CYP2B6*6 |  | rs3745274 and rs2279343 | haplotype | Hattinger 2016[4] | Oncotarget. | Italy | 196/470 | WT,HET,VAR  129,53,14 | WT,HET,VAR  273,178,19 |  | WT  HET  VAR | 0.03 |
|  | CYP2B6*7 |  | rs3745274 and rs2279343 and rs3211371 | Haplotype | Hattinger 2016[4] | Oncotarget. | Italy | 196/470 | WT,HET,VAR  194,2,0 | WT,HET,VAR  451,19,0 |  | WT  HET  VAR | 0.032 |
|  | CYP2C9 |  | rs1799853 | Coding sequence variant ,missense variant | Hattinger 2016[4] | Oncotarget. | Italy | 196/470 | CC,CT,TT  147,45,4 | CC,CT,TT  338,122,10 | 0.8 | CC  CT  TT | >0.05 |
|  |  |  | rs1057910 | Coding sequence variant ,missense variant | Hattinger 2016[4] | Oncotarget. | Italy | 196/470 | AA,AC,CC  169,23,3 | AA,AC,CC  385,80,5 | 0.9 | AA  AC  CC | >0.05 |
|  | CYP3A4 | 10 | rs2740574 | 2KB upsteam variant, upstream transcript variant | Hattinger 2016[4] | Oncotarget. | Italy | 196/470 | AA,AG,GG  178,18,0 | AA,AG,GG  446,24,0 | 0.6 | AA  AG  GG | >0.05 |
|  | COL18A1 | 21 | c.4309G> A  (p.D104N) |  | Bi 2016[11] | Sci.reeports | China | 236/418 | WT,HET,VAR  205,23,8 | WT,HET,VAR  376,39,3 |  | WT  HET  VAR | >0.05 |
|  | RAD51 | 15 | rs1801320 | 2KB upstream variant,5 prime UTR variant,genic upstream transcript variant,intron variant, upstream transcript | Jin 2015[16] | Pak J Med Sci | China | 148/296 | GG,GC+CC  101,47 | GG,GC+CC  213,85 | - | GC+CC vs GG | 0.48 |
|  |  |  |  |  | Goricar 2015[52] | J Med Biochem. | Slovenia | 79/373 | GG,GC+CC  69,9 | GG,GC+CC  304,69 | - | GC+CC vs GG | 0.337 |
|  |  |  | rs1801321 | 2KB upstream variant,5 prime UTR variant,genic upstream transcript variant, intron variant, upstream transcript | Jin 2015[16] | Pak J Med Sci | China | 148/296 | GG,GT+TT  88,60 | GG,GT+TT  187,111 |  | GT+TT vs GG | 0.5 |
|  |  |  |  |  | Goricar 2015[52] | J Med Biochem. | Slovenia | 79/373 | GG,GT+TT  25,54 | GG,GT+TT  133,240 |  | GT+TT vs GG | 0.802 |
|  |  |  | rs12593359 | 3 prime UTR variant, downstream transcript variant, genic downstream transcript variant | Jin 2015[16] | Pak J Med Sci | China | 148/296 | TT,TG+GG  49,99 | TT,TG+GG  105,193 |  | TG+GG vs TT | 0.66 |
|  |  |  |  |  | Goricar 2015[52] | J Med Biochem. | Slovenia | 79/373 | TT,TG+GG  22,57 | TT,TG+GG  103,270 |  | TG+GG vs TT | 0.702 |
|  | Fas | 10 | -1377 G>A |  | Koshkina 2007[75] | J Pediatr Hematol Oncol. | USA | 123/510 | GG,GA,AA  99,22,2 | GG,GA,AA  400,100,10 | 0.2 | GG  GA  AA | 0.88 |
|  |  |  | rs1800682 | Genic upstream transcript variant, upstream transcript variant,intron variant | Koshkina 2007[75] | J Pediatr Hematol Oncol. | USA | 123/510 | AA,AG,GG  31,21,31 | AA,AG,GG  145,251,114 | 0.8 | AA  AG  GG | 0.7 |
|  |  |  | exon 3 18272 A>G |  | Koshkina 2007[75] | J Pediatr Hematol Oncol. | USA | 123/510 | AA,AG,GG  99,24,0 | AA,AG,GG  449,61,0 | 0.2 | AA  AG  GG | 0.028 |
|  |  |  | exon 7 22628 C>T |  | Koshkina 2007[75] | J Pediatr Hematol Oncol. | USA | 123/510 | CC,CTC,TT  67,51,5 | CC,CT,TT  288,199,31 | 0.6 | CC  CT  TT | 0.66 |

*p<0.0167 was considered significant after Bonferroni correction, ^ Ins=Insertion,# DELINS=Deletion/Insertion, WT=wild type, HET=heterozygous, VAR=variant,STR=Short tandem repeats (microsatellite) variation

NH=No homoyzous, SD=Standard Deviation

#Assay identifier from http://snp500cancer.nci.nih.gov

Table S4:Genetic variants not associated with osteosarcoma

| SNV | chr | Model | OR (95% CI) P-value | | I^2^% | Q- value | Heterogeneity  P-value | Cases vs Controls | #Studies in Asian | Asian OR  (95% CI) | | #Studies  Caucasian | | Caucasian  OR (95% CI) | |  |
| --- | --- | --- | --- | --- | --- | --- | --- | --- | --- | --- | --- | --- | --- | --- | --- | --- |
| APEX1 rs1130409 | 14 | TG vs TT | | 1.06 [0.18; 6.2] 0.74 | 9 | 1.1 | 0.29 | 574 vs 1086 (2) | 1[3] | 0.96 [0.72; 1.28] | | 1[4] | | 1.29 [0.81; 2.07] | |  |
|  |  | GG vs TT | | 1.05 [0.05; 24.22] 0.87 | 59 | 2.46 | 0.12 | 574 vs 1086 (2) | 1[3] | 0.85 [0.58; 1.23] | | 1[4] | | 1.39 [0.85; 2.29] | |  |
|  |  | TG+GG vs TT | | 1.06 [0.12; 9.75] 0.79 | 46 | 1.84 | 0.17 | 574 vs 1086 (2) | 1[3] | 0.93 [0.71; 1.21] | | 1[4] | | 1.33 [0.85; 2.08] | |  |
|  |  | GG vs TT+TG | | 1.06 [0.12; 9.75] 0.79 | 25 | 1.34 | 0.25 | 574 vs 1086 (2) | 1[3] | 0.87 [0.62; 1.21] | | 1[4] | | 1.15 [0.81; 1.64] | |  |
|  |  | G vs T | | 1.02 [0.24; 4.32] 0.88 | 55 | 2.21 | 0.14 | 574 vs 1086 (2) | 1[3] | 0.92 [0.77; 1.11] | | 1[4] | | 1.16 [0.91; 1.47] | |  |
| VEGF rs833061 | 6 | TC vs TT | | 1.29 [0.35; 4.76] 0.24 | 0 | 0.38 | 0.53 | 354 vs 358 (2) | 2[64, 65] | 1.29 [0.35; 4.76] | | 0 | |  | |  |
|  |  | CC vs TT | | 1.61 [0.04; 65.6] 0.34 | 37 | 1.39 | 0.24 | 354 vs 358 (2) | 2[64, 65] | 1.61 [0.04; 65.6] | | 0 | |  | |  |
|  |  | CC+TC vs TT | | 1.36 [0.2; 9.42] 0.29 | 0 | 0.84 | 0.36 | 354 vs 358 (2) | 2[64, 65] | 1.36 [0.2; 9.42] | | 0 | |  | |  |
|  |  | CC vs TT+TC | | 1.39 [0.08; 24] 0.38 | 0 | 0.17 | 0.27 | 354 vs 358 (2) | 2[64, 65] | 1.39 [0.08; 24] | | 0 | |  | |  |
|  |  | C vs T | | 1.27 [0.23; 6.94] 0.3253 | 35 | 1.53 | 0.23 | 354 vs 358 (2) | 2[64, 65] | 1.27 [0.23; 6.94] | | 0 | |  | |  |
| ERCC1 rs11615 | 19 | TC vs TT | | 0.92 [0.60; 1.39]0.56 | 0 | 2.65 | 0.45 | 735 vs 1582 (4) | 1[17] | 0.72 [0.44; 1.18] | | 3[4, 18, 19] | | 1.01 [0.55; 1.87] | |  |
|  |  | CC vs TT | | 0.94 [0.46; 1.95]0.82 | 57.7 | 7.09 | 0.07 | 735 vs 1582 (4) | 1[17] | 0.60 [0.37;1.0] | | 3[4, 18, 19] | | 1.24 [0.67; 2.29] | |  |
|  |  | TC+CC vs TT | | 0.97 [0.70; 1.3] 0.80 | 14.8 | 4.7 | 0.32 | 883 vs 1878 (5) | 2[16, 17] | 0.90 [0.66; 1.22] | | 3[4, 18, 19] | | 1.05 [0.80 1.38] | |  |
|  |  | TT vs CC+TC | | 0.99 [0.64; 1.52] 0.92 | 46.7 | 5.62 | 0.13 | 735 vs 1582 (4) | 1[17] | 0.78 [0.61; 1.0] | | 3[4, 18, 19] | | 1.18 [0.74; 1.9] | |  |
|  |  | C vs T | | 0.96 [0.70; 1.30] 0.69 | 52.4 | 6.3 | 0.1 | 735 vs 1582 (4) | 1[17] | 0.79 [0.65; 1.0] | | 3[4, 18, 19] | | 1.08 [0.81; 1.44] | |  |
| ERCC2 rs13181 | 19 | TG vs TT | | 1.02 [0.24; 4.33] 0.89 | 0 | 0.57 | 0.45 | 365 vs 849 (3) | 1[20] | 1.18 [0.72; 1.94] | | 2[4,19] | | 0.93 [0.65; 1.34] | |  |
|  |  | GG vs TT | | 1.07 [0.71; 1.62] 0.74 | 22.4 | 1.29 | 0.26 | 337 vs 772 (2) | 1[20] | 1.55 [0.73; 3.29] | | 1[4] | | 0.92[0.56; 1.51] | |  |
|  |  | TG+GG vs TT | | 1.04 [0.76; 1.43]0.69 | 0 | 2.33 | 0.51 | 513 vs 1145(4) | 2[20,16] | 1.19 [0.89; 1.61] | | 2[4, 19] | | 0.89 [0.65; 1.23] | |  |
|  |  | GG vs TT+TG | | 1.11 [0.08; 16.16] 0.72 | 0.6 | 1.01 | 0.32 | 337 vs 772 (2) | 1[20] | 1.49 [0.71; 3.13] | | 1[4] | | 0.95 [0.60; 1.51] | |  |
|  |  | G vs T | | 1.02 [0.5; 2.02] 0.92 | 47.6 | 1.91 | 0.17 | 365 vs 849 (3) | 1[20] | 1.29 [0.90; 1.86] | | 2[4,19] | | 0.95 [0.75; 1.21] | |  |
| ERCC3 rs4150441 | 19 | GA vs GG | | 1.31 [0.12; 14.56]0.40 | 60 | 2.48 | 0.122 | 522 vs 1047(2) | 2[17, 20] | 1.31 [0.12; 14.56] | | 0 | |  | |  |
|  |  | AA vs GG | | 2.12 [0.016; 85.55]0.30 | 71 | 3.45 | 0.06 | 522 vs 1047(2) | 2[17, 20] | 2.12 [0.016; 85.55] | | 0 | |  | |  |
|  |  | GA+AA vs GG | | 1.54 [0.07; 31.6] 0.32 | 74 | 3.81 | 0.051 | 522 vs 1047(2) | 2[17, 20] | 1.54 [0.07; 31.6] | | 0 | |  | |  |
|  |  | AA vs GG+GA | | 1.85 [0.03; 97.96] 0.30 | 60 | 2.51 | 0.11 | 522 vs 1047(2) | 2[17, 20] | 1.85 [0.03; 97.96] | | 0 | |  | |  |
|  |  | G vs A | | 1.46 [0.09; 22.1] 0.33 | 82 | 5.48 | 0.019 | 522 vs 1047(2) | 2[17, 20] | 1.46 [0.09; 22.1] | | 0 | |  | |  |
| GRM4 rs1906953 | 6 | TC vs TT | | 1.04 [0.69; 1.82] 0.2 | 0 | 0.06 | 0.81 | 294 vs 384 (2) | 2[21, 22] | 1.04 [0.69; 1.82] | | 0 | |  | |  |
|  |  | CC vs TT | | 0.85 [0.60; 1.20]0.4 | 99 | 9.03 | 0.0027 | 294 vs 384 (2) | 2[21, 22] | 0.85 [0.60; 1.20] | | 0 | |  | |  |
|  |  | CC+TC vs TT | | 0.99 [0.70; 1.40]0.9 | 91 | 11.14 | 0.0008 | 294 vs 384 (2) | 2[21, 22] | 0.99 [0.70; 1.40] | | 0 | |  | |  |
|  |  | CC vs TT+TC | | 0.98 [0.02;2.22] 0.9 | 66 | 14.7 | 0..001 | 294 vs 384 (2) | 2[21, 22] | 0.98 [0.02;2.22] | | 0 | |  | |  |
|  |  | C vs T | | 0.94 [0.75; 1.16]0.5 | 94 | 15.71 | 0­.0001 | 294 vs 384 (2) | 2[21, 22] | 0.94 [0.75; 1.16] | | 0 | |  | |  |
| MDM2 rs1690916 | 12 | GA vs GG | | 0.91 [0.28; 2.40] 0.81 | 67 | 9.02 | 0.03 | 246 vs 1718(4) | 0 |  | | 4[45–47] | | 0.91 [0.28; 2.40] | |  |
|  |  | AA vs GG | | 0.8 [0.12; 5.13] 0.72 | 75 | 11.9 | 0.007 | 246 vs 1718(4) | 0 |  | | 4[45–47] | | 0.8 [0.12; 5.13] | |  |
|  |  | GA +AA vs GG | | 0.70 [0.18; 2.79] 0.47 | 62 | 7.82 | 0.051 | 246 vs 1718(4) | 0 |  | | 4[45–47] | | 0.70 [0.18; 2.79] | |  |
|  |  | GG+GA vs AA | | 0.72[0.25;2.10] 0.40 | 50 | 5.9 | 0.11 | 246 vs 1718(4) | 0 |  | | 4[45–47] | | 0.72[0.25;2.10] | |  |
|  |  | A vs G | | 0.77 [0.34; 1.78] 0.40 | 70 | 10.11 | 0.018 | 246 vs 1718(4) | 0 |  | | 4[45–47] | | 0.77 [0.34; 1.78] | |  |
| MDM2 rs2279744 | 12 | GT vs TT | | 1.20 [0.90; 1.62] 0.15 | 0 | 3.5 | 0.50 | 440 vs 1919 (5) | 0 |  | | 5[4, 45, 47, 48] | | 1.20 [0.90; 1.62] | |  |
|  |  | GG vs TT | | 1.57[0.62; 4.0] 0.25 | 68.4 | 12.7 | 0.01 | 619 vs 2352 (5) | 0 |  | | 5[4, 45, 47, 48] | | 1.57[0.62; 4.0] | |  |
|  |  | GT +GG vs TT | | 1.0[0.39; 2.55] 0.99 | 87.2 | 31.27 | < 0.0001 | 619 vs 2352 (5) | 0 |  | | 5[4, 45, 47, 48] | | 1[0.39; 2.55] | |  |
|  |  | GG vs TT+GT | | 1.21 [0.42; 3.52] 0.64 | 74.8 | 36.72 | < 0.0001 | 619 vs 2352 (5) | 0 |  | | 5[4, 45, 47, 48] | | 1.21 [0.42; 3.52] | |  |
|  |  | G vs T | | 1.057[0.53; 2.10] 0.83 | 89 | 36.27 | < 0.0001 | 619 vs 2352 (5) | 0 |  | | 5[4, 45, 47, 48] | | 1.057[0.53; 2.10] | |  |
| GSTM1 gen_null | 1 | Null vs. Present | | 1.10[0.62; 1.96] 0.7 | 40.1 | 10.2 | 0.12 | 654 vs 1573 (7) | 3[25–27] | 1.42 [0.84; 2.40] | | 4[4, 23, 24, 28] | | 0.80 [0.22; 2.95] | |  |
| GSTM3 rs138440339 | 1 | TC vs TT | | 1.13[0.58; 2.19]0.7 | 44 | 1.8 | 0.18 | 63 vs 360 (2) | 0 |  | | 2[23, 28] | | 1.13[0.58; 2.19] | |  |
|  |  | CC vs TT | | 1.01 [0.06; 16.55] 0.96 | 0 | 0.03 | 0.87 | 63 vs 360 (2) | 0 |  | | 2[23, 28] | | 1.01 [0.06; 16.55] | |  |
|  |  | TC+CC vs TT | | 1.11[0.58; 2.14]0.75 | 35 | 1.53 | 0.22 | 63 vs 360 (2) | 0 |  | | 2[23, 28] | | 1.11[0.58; 2.14] | |  |
|  |  | CC vs TT+TC | | 0.97[0.08; 11.33] 0.98 | 0 | 0.07 | 0.80 | 63 vs 360 (2) | 0 |  | | 2[23, 28] | | 0.97[0.08; 11.33] | |  |
|  |  | C vs T | | 1.007 [0.13; 7.57]0.97 | 0 | 0.3 | 0.59 | 63 vs 360 (2) | 0 |  | | 2[23, 28] | | 1.007 [0.13; 7.57] | |  |
| GSTT1  gen_null | 22 | Null vs present | | 1.16 [0.83; 1.62] 0.32 | 27.1 | 6.68 | 0.23 | 602 vs 1468 (6) | 2[25] | 1.38 [0.05; 36.06] | | 4[4, 23, 24, 28] | | 0.99 [0.64; 1.53] | |  |
| PRKCG rs8103851 | 19 | CG vs CC | | 0.97[0.69;1.40]0.48 | 0 | 0.07 | 0.79 | 998 vs 998 (2) | 2[53, 54] | | 0.97[0.69;1.40] | | 0 | |  | |
|  |  | GG vs CC | | 0.90 [0.09; 9.06] 0.66 | 51 | 2.06 | 0.15 | 998 vs 998 (2) | 2[53, 54] | | 0.90 [0.09; 9.06] | | 0 | |  | |
|  |  | CG+GG vs CC | | 0.94 [0.37; 2.42] 0.58 | 0 | 0.62 | 0.43 | 998 vs 998 (2) | 2[53, 54] | | 0.94 [0.37; 2.42] | | 0 | |  | |
|  |  | GG v s CC+CG | | 0.91[0.11; 7.52] 0.68 | 54 | 2.19 | 0.14 | 998 vs 998 (2) | 2[53, 54] | | 0.91[0.11; 7.52] | | 0 | |  | |
|  |  | G vs C | | 0.95 [0.30; 2.96] 0.66 | 41 | 1.9 | 0.17 | 998 vs 998 (2) | 2[53, 54] | | 0.95 [0.30; 2.96] | | 0 | |  | |
| TGF-β1 rs1800470 | 19 | TC vs TT | | 0.98 [0.68; 1.4] 0.90 | 85 | 6.81 | 0.009 | 326vs 352 (2) | 2[57,58] | 0.98 [0.68; 1.4] | | 0 | |  | |  |
|  |  | CC vs TT | | 1.16 [0.77; 1.75] 0.47 | 92.2 | 13 | 0.0003 | 326vs 352 (2) | 2[57,58] | 1.16 [0.77; 1.75] | | 0 | |  | |  |
|  |  | TC+CC vs TT | | 1.02 [0.73; 1.42] 0.91 | 91.2 | 11.31 | 0.0008 | 326vs 352 (2) | 2[57,58] | 1.02 [0.73; 1.42] | | 0 | |  | |  |
|  |  | CC vs TT+TC | | 1.20 [0.84; 1.70] 0.31 | 85.5 | 6.9 | 0.0085 | 326vs 352 (2) | 2[57,58] | 1.20 [0.84; 1.70] | | 0 | |  | |  |
|  |  | C vs T | | 1.10 [0.87; 1.33] 0.49 | 93 | 14.32 | 0.0002 | 326vs 352 (2) | 2[57,58] | 1.10 [0.87; 1.33] | | 0 | |  | |  |
| TGF-β1 rs1800469 | 19 | CT vs CC | | 0.86 [0.03; 30.30]0.69 | 54 | 2.19 | 0.14 | 326 vs 352 (2) | 2[57,58] | 0.86 [0.03; 30.30] | | 0 | |  | |  |
|  |  | TT vs CC | | 1.03 [0.2; 5.43] 0.88 | 0 | 0.36 | 0.55 | 326 vs 352 (2) | 2[57,58] | 1.03 [0.20; 5.43] | | 0 | |  | |  |
|  |  | CT+TT vs CC | | 0.94 [0.06; 13.73]0.80 | 32 | 1.48 | 0.22 | 326 vs 352 (2) | 2[57,58] | 0.94 [0.06; 13.73] | | 0 | |  | |  |
|  |  | TT vs CT+CC | | 1.13 [0.65; 1.98] 0.22 | 0 | 0.06 | 0.81 | 326 vs 352 (2) | 2[57,58] | 1.13 [0.65; 1.98] | | 0 | |  | |  |
|  |  | T vs C | | 1.02 [0.48; 2.18] 0.80 | 0 | 0.3 | 0.60 | 326 vs 352 (2) | 2[57,58] | 1.02 [0.48; 2.18] | | 0 | |  | |  |
| TP53 rs1642785 | 17 | CG vs CC | | 2.24 [0; 124.85] 0.58 | 95.6 | 22.57 | < 0.0001 | 295 vs 538 (2) | 0 |  | | 2[61,4] | | 2.24 [0; 124.85] | |  |
|  |  | GG vs CC | | 22.07 [0; 110.80] 0.21 | 72 | 3.57 | 0.06 | 295 vs 538 (2) | 0 |  | | 2[61,4] | | 22.07 [0; 110.80] | |  |
|  |  | CG+ GG vs CC | | 14.72 [0.48; 452.29]0.06 | 0 | 0.95 | 0.42 | 295 vs 538 (2) | 0 |  | | 2[61,4] | | 14.72 [0.48; 452.29] | |  |
|  |  | GG vs CC+CG | | 3.66[0; 129.08] 0.52 | 97.6 | 42.41 | < 0.0001 | 295 vs 538 (2) | 0 |  | | 2[61,4] | | 3.66[0; 129.08] | |  |
|  |  | G vs C | | 3.18 [0; 116.50] 0.46 | 98 | 51.2 | < 0.0001 | 295 vs 538 (2) | 0 |  | | 2[61,4] | | 3.18 [0; 116.50] | |  |
| NBN rs1805794 | 8 | GC vs GG | | NA |  |  |  |  |  |  | |  | |  | |  |
|  |  | GG vs GG | | NA |  |  |  |  |  |  | |  | |  | |  |
|  |  | GC+CC vs GG | | 1.15[0.80; 1.64]0.50 | 73 | 3.76 | 0.04 | 227 vs 671 (2) | 1[16] | 1.37 [0.91; 2.06] | | 1[52] | | 0.60 [0.29; 1.25] | |  |
|  |  | CC vs GG+GC | | NA |  |  |  |  |  |  | |  | |  | |  |
|  |  | G vs C | | NA |  |  |  |  |  |  | |  | |  | |  |
| RAD51 rs1801320 | 15 | GC vs GG | | NA |  |  |  |  |  |  | |  | |  | |  |
|  |  | GG vs GG | | NA |  |  |  |  |  |  | |  | |  | |  |
|  |  | GC+CC vs GG | | 0.87 [0.01; 7.9] 0.76 | 64 | 2.75 | 0.097 | 227 vs 671 (2) | 1[16] | 1.17 [0.76; 1.79] | | 1[52] | | 0.57 [0.27; 1.19] | |  |
|  |  | CC vs GG+GC | | NA |  |  |  |  |  |  | |  | |  | |  |
|  |  | G vs C | | NA |  |  |  |  |  |  | |  | |  | |  |
| RAD51 rs1801321 | 15 | GT vs GG | | NA |  |  |  |  |  |  | |  | |  | |  |
|  |  | TT vs GG | | NA |  |  |  |  |  |  | |  | |  | |  |
|  |  | GT+TT vs GG | | 0.17 [0.9; 1.5] 0.08 | 0 | 0.02 | 0.90 | 227 vs 671 (2) | 1[16] | 1.15 [0.77; 1.72] | | 1[52] | | 1.20 [0.71; 2.01] | |  |
|  |  | TT vs GG+GT | | NA |  |  |  |  |  |  | |  | |  | |  |
|  |  | G vs T | | NA |  |  |  |  |  |  | |  | |  | |  |
| RAD51 rs12593359 | 15 | TG vs TT | | NA |  |  |  |  |  |  | |  | |  | |  |
|  |  | GG vs TT | | NA |  |  |  |  |  |  | |  | |  | |  |
|  |  | TG+GG vs TT | | 1.06 [0.55; 2.03] 0.45 | 0 | 0.09 | 0.76 | 227 vs 671 (2) | 1[16] | 1.10 [0.72; 1.67] | | 1[52] | | 0.99 [0.58; 1.70] | |  |
|  |  | GG vs TT+TG | | NA |  |  |  |  |  |  | |  | |  | |  |
|  |  | G vs T | | NA |  |  |  |  |  |  | |  | |  | |  |

**References**

1. Mirabello, L., Berndt, S. I., Seratti, G. F., Burdett, L., Yeager, M., Chowdhury, S., … R.N., H. (2010). Genetic variation at chromosome 8q24 in osteosarcoma cases and controls. *Carcinogenesis*, *31*(8), 1400–1404. https://doi.org/http://dx.doi.org/10.1093/carcin/bgq117

2. He, M., Wu, Y., Zhao, J., & Wang, Z. (2013). PIK3CA and AKT gene polymorphisms in susceptibility to osteosarcoma in a Chinese population. *Asian Pacific journal of cancer prevention : APJCP*, *14*(9), 5117–5122. Retrieved from http://ovidsp.ovid.com/ovidweb.cgi?T=JS&PAGE=reference&D=emed14&NEWS=N&AN=602068938

3. Xiao, X., Yang, Y., Ren, Y., Zou, D., Zhang, K., & Wu, Y. (2017). rs1760944 Polymorphism in the APE1 Region is Associated with Risk and Prognosis of Osteosarcoma in the Chinese Han Population. *Scientific reports*, *7*(1), 9331. https://doi.org/10.1038/s41598-017-09750-9

4. Hattinger, C., Biason, P., Iacoboni, E., Gagno, S., Fanelli, M., Tavanti, E., … G., T. (2016). Candidate germline polymorphisms of genes belonging to the pathways of four drugs used in osteosarcoma standard chemotherapy associated with risk, survival and toxicity in non-metastatic high-grade osteosarcoma. *Oncotarget*, *7*(38), 61970–61987. https://doi.org/http://dx.doi.org/10.18632/oncotarget.11486

5. Zhao, J., Xu, H., He, M., Wang, Z., Wu, Y., J., Z., … Wu, Y. (2014). Rho GTPase-activating protein 35 rs1052667 polymorphism and osteosarcoma risk and prognosis. *BioMed Research International*, *2014*, 396947. https://doi.org/http://dx.doi.org/10.1155/2014/396947

6. Cong, Y., Li, C.-J., Zhao, J.-N., Liu, X.-Z., & Shi, X. (2015). Associations of polymorphisms in the bone morphogenetic protein-2 gene with risk and prognosis of osteosarcoma in a Chinese population. *Tumour biology : the journal of the International Society for Oncodevelopmental Biology and Medicine*, *36*(3), 2059–2064. https://doi.org/10.1007/s13277-014-2813-0

7. Wang, W., Song, H., Liu, J., Song, B., Wang, W., Song, H., … Cao, X. (2011). CD86+1057G/A polymorphism and susceptibility to osteosarcoma. *DNA and Cell Biology*, *30*(11), 925–929. https://doi.org/http://dx.doi.org/10.1089/dna.2011.1211

8. Zhang, H., Mao, J., Huahui, Z., Mao, J.-S., Hu, W.-F., Huahui, Z., … Wei-F, H. (2019). Functional genetic single-nucleotide polymorphisms (SNPs) in cyclin-dependent kinase inhibitor 2A/B (CDKN2A/B) locus are associated with risk and prognosis of osteosarcoma in Chinese populations. *Medical Science Monitor*, *25*, 1307–1313. https://doi.org/http://dx.doi.org/10.12659/MSM.915001

9. He, M., Wang, Z., Zhao, J., Chen, Y., & Wu, Y. (2014). COL1A1 polymorphism is associated with risks of osteosarcoma susceptibility and death. *Tumour biology : the journal of the International Society for Oncodevelopmental Biology and Medicine*, *35*(2), 1297–1305. https://doi.org/10.1007/s13277-013-1172-6

10. Guo, Z., Zhang, T., Wu, J., Wang, H., & Liu, Z. (2015). Genetic polymorphisms in COL18A1 influence the development of osteosarcoma. *International journal of clinical and experimental pathology*, *8*(9), 11531–11536. Retrieved from http://ovidsp.ovid.com/ovidweb.cgi?T=JS&PAGE=reference&D=emed16&NEWS=N&AN=615198785

11. Bi, W.-Z., Li, D.-W., Luo, S., Song, Z.-G., Wang, Y. Y. Y., Jin, H., … M., X. (2015). A high risk of osteosarcoma in individuals who are homozygous for the p.D104N in endostatin. *Scientific reports*, *5*, 16392. https://doi.org/http://dx.doi.org/10.1038/srep16392

12. Qiao, G., Miao, H., Yi, Y., Wang, D., Liu, B., Zhang, Y., & Chen, X. (2016). Genetic association between CTLA-4 variations and osteosarcoma risk: Case-control study. *International Journal of Clinical and Experimental Medicine*, *9*(6), 9598–9602. Retrieved from https://www.scopus.com/inward/record.uri?eid=2-s2.0-84977134465&partnerID=40&md5=06a3f13cdc1d02fba46eabbbf44e27b7

13. Liu, Y., He, Z., Feng, D., Shi, G., Gao, R., Wu, X., … Wen, Y. (2011). Cytotoxic T-lymphocyte antigen-4 polymorphisms and susceptibility to osteosarcoma. *DNA and Cell Biology*, *30*(12), 1051–1055. https://doi.org/http://dx.doi.org/10.1089/dna.2011.1269

14. Wang, W., Wang, J., Song, H., Liu, J., Song, B., & Cao, X. (2011). Cytotoxic T-lymphocyte antigen-4 +49G/A polymorphism is associated with increased risk of osteosarcoma. *Genetic testing and molecular biomarkers*, *15*(7–8), 503–506. https://doi.org/10.1089/gtmb.2010.0264

15. Bilbao-Aldaiturriaga, N., Patino-Garcia, A., Martin-Guerrero, I., Bilbao-Aldaiturriaga, N., Patino-Garcia, A., & Martin-Guerrero, I. (2017). Cytotoxic T lymphocyte-associated antigen 4 rs231775 polymorphism and osteosarcoma. *Neoplasma*, *64*(2), 299–304. https://doi.org/http://dx.doi.org/10.4149/neo_2017_218

16. G., J., M., W., W., C., W., S., J., Y., G, J., … Yin, J. (2015). Single nucleotide polymorphisms of nucleotide excision repair and homologous recombination repair pathways and their role in the risk of osteosarcoma. *Pakistan Journal of Medical Sciences*, *31*(2), 269–273. https://doi.org/http://dx.doi.org/10.12669/pjms.312.6569

17. Xu, Q., Zhang, Z., Sun, W., Hu, B., Q, X., Z, Z., … W., S. (2017). Haplotype analysis on relationship of ERCC2 and ERCC3 gene polymorphisms with osteosarcoma risk in Chinese young population. *Mammalian Genome*, *28*(5–6), 227–233. https://doi.org/http://dx.doi.org/10.1007/s00335-017-9693-8

18. Biason, P., Hattinger, C. M., Innocenti, F., Talamini, R., Alberghini, M., Scotlandi, K., … Serra, M. (2012). Nucleotide excision repair gene variants and association with survival in osteosarcoma patients treated with neoadjuvant chemotherapy. *Pharmacogenomics Journal*, *12*(6), 476–483. https://doi.org/http://dx.doi.org/10.1038/tpj.2011.33

19. Gómez-Díaz, B., De La Luz Ayala-Madrigal, M., Gutiérrez-Angulo, M., Valle-Solis, A. E., Linares-González, L. M., González-Guzmán, R., … López-Hernández, L. B. (2015). Analysis of ercc1 and ercc2 gene variants in osteosarcoma, colorectal and breast cancer. *Oncology Letters*, *9*(4), 1657–1661. https://doi.org/10.3892/ol.2015.2894

20. Ma, X., Zhang, Y., Sun, T. S., & Yao, J. H. (2016). Role of ERCC2 and ERCC3 gene polymorphisms in the development of osteosarcoma. *Genetics and molecular research : GMR*, *15*(1). https://doi.org/10.4238/gmr.15017302

21. Wang, K., Zhao, J., He, M., Fowdur, M., Jiang, T., Kun, W., … Shuju, L. (2016). Association of GRM4 gene polymorphisms with susceptibility and clinicopathological characteristics of osteosarcoma in Guangxi Chinese population. *Tumor Biology*, *37*(1), 1105–1112. https://doi.org/10.1007/s13277-015-3904-2

22. Jiang, C., Chen, H., Shao, L., Chaoyin, J., Hua, C., Lei, S., & Yang, D. (2014). GRM4 gene polymorphism is associated with susceptibility and prognosis of osteosarcoma in a Chinese Han population. *Medical Oncology*, *31*(7), 50. https://doi.org/http://dx.doi.org/10.1007/s12032-014-0050-4

23. Barnette, P., Scholl, R., Blandford, M., Ballard, L., Tsodikov, A., Magee, J., … Keller, C. (2004). High-Throughput Detection of Glutathione S-Transferase Polymorphic Alleles in a Pediatric Cancer Population. *Cancer Epidemiology Biomarkers and Prevention*, *13*(2), 304–313. https://doi.org/http://dx.doi.org/10.1158/1055-9965.EPI-03-0178

24. Salinas-Souza, C., Petrilli, A. S., & de Toledo, S. R. C. (2010). Glutathione S-transferase polymorphisms in osteosarcoma patients. *Pharmacogenetics and Genomics*, *20*(8), 507–515. https://doi.org/http://dx.doi.org/10.1097/FPC.0b013e32833caa45

25. Lu, X.-F. F., Yang, W.-L. L., Wan, Z.-H., Li, J., Bi, Z.-G., X.F., L., … J., L. (2011). Glutathione S-transferase polymorphisms and bone tumor risk in China. *Asian Pacific journal of cancer prevention : APJCP*, *12*(12), 3357–3360. Retrieved from http://ovidsp.ovid.com/ovidweb.cgi?T=JS&PAGE=reference&D=emed12&NEWS=N&AN=365353534

26. Li, L., Li, J. G., Liu, C. Y., & Ding, Y. J. (2015). Effect of CYP1A1 and GSTM1 genetic polymorphisms on bone tumor susceptibility. *Genetics and molecular research : GMR*, *14*(4), 16600–16607. https://doi.org/10.4238/2015.December.11.7

27. Qu, W. R., Wu, J., & Li, R. (2016). Contribution of the GSTP1 gene polymorphism to the development of osteosarcoma in a Chinese population. *Genetics and Molecular Research*, *15*(3), gmr.15038034. https://doi.org/http://dx.doi.org/10.4238/gmr.15038034

28. Moghimi, M., Sobhan, M. R., Jarahzadeh, M. H., Morovati-Sharifabad, M., Aghili, K., Ahrar, H., … M., Z.-S. (2019). Association of GSTM1, GSTT1, GSTM3, and GSTP1 Genes Polymorphisms with Susceptibility to Osteosarcoma: a Case- Control Study and Meta-Analysis. *Asian Pacific journal of cancer prevention : APJCP*, *20*(3), 675–682. https://doi.org/10.31557/APJCP.2019.20.3.675

29. He, T.-D., Xu, D., Sui, T., Zhu, J.-K., Wei, Z.-X., & Wang, Y.-M. (2017). Association between H19 polymorphisms and osteosarcoma risk. *European review for medical and pharmacological sciences*, *21*(17), 3775–3780. Retrieved from http://ovidsp.ovid.com/ovidweb.cgi?T=JS&PAGE=reference&D=emed18&NEWS=N&AN=623064590

30. Xin, D.-J., Shen, G.-D., & Song, J. (2015). Single nucleotide polymorphisms of HER2 related to osteosarcoma susceptibility. *International journal of clinical and experimental pathology*, *8*(8), 9494–9499. Retrieved from http://ovidsp.ovid.com/ovidweb.cgi?T=JS&PAGE=reference&D=emed16&NEWS=N&AN=615877573

31. Zhou, Q., Chen, F., Fei, Z., Zhao, J., Liang, Y., Pan, W., … Zheng, D. (2016). Genetic variants of lncRNA HOTAIR contribute to the risk of osteosarcoma. *Oncotarget*, *7*(15), 19928–19934. https://doi.org/http://dx.doi.org/10.18632/oncotarget.7957

32. Savage, S. A., Woodson, K., Walk, E., Modi, W., Liao, J., Douglass, C., … Chanock, S. J. (2007). Analysis of genes critical for growth regulation identifies Insulin-like Growth Factor 2 Receptor variations with possible functional significance as risk factors for osteosarcoma. *Cancer epidemiology, biomarkers & prevention : a publication of the American Association for Cancer Research, cosponsored by the American Society of Preventive Oncology*, *16*(8), 1667–1674. https://doi.org/10.1158/1055-9965.EPI-07-0214

33. He, Y., Liang, X., Meng, C., Shao, Z., Gao, Y., Wu, Q., … H, W. (2014). Genetic polymorphisms of interleukin-1 beta and osteosarcoma risk. *International orthopaedics*, *38*(8), 1671–1676. https://doi.org/10.1007/s00264-014-2374-2

34. Shang, Z., Zhang, F., Zhou, C., Wei, G., Yan, C., Jin, T., … Liang, J. (2017). Association of IL-6 -634C/G polymorphism and the risk of osteosarcoma in a chinese population. *Biomedical Research (India)*, *28*(22), 9761–9763. Retrieved from https://www.biomedres.info/biomedical-research/association-of-il6-634cg-polymorphism-and-the-risk-of-osteosarcoma-in-a-chinese-population-9348.html

35. Qi, Y., Zhao, C., Li, H., Zhang, B., Tada, K., Abe, H., & Tada, M. (2016). Genetic variations in interleukin-6 polymorphism and the association with susceptibility and overall survival of osteosarcoma. *Tumour biology : the journal of the International Society for Oncodevelopmental Biology and Medicine*, *37*(7), 9807–9811. https://doi.org/10.1007/s13277-016-4876-6

36. Oliveira, I. D., Petrilli, A. S., Tavela, M. H., Zago, M. A., & de Toledo, S. R. C. (2007). TNF-alpha, TNF-beta, IL-6, IL-10, PECAM-1 and the MPO inflammatory gene polymorphisms in osteosarcoma. *Journal of pediatric hematology/oncology*, *29*(5), 293–297. https://doi.org/10.1097/MPH.0b013e3180587e69

37. Tian, X., Chen, Y., Fang, G., Zhou, Z., Dong, J., & Guo, Q. (2016). Association between IL-8 rs4073 polymorphisms and osteosarcoma risk in Chinese population: A case control study. *International Journal of Clinical and Experimental Medicine*, *9*(7), 13172–13177. Retrieved from http://www.ijcem.com/files/ijcem0020748.pdf

38. Y, C., Y, Y., S, L., S, Z., H, J., & J, D. (2016). Association between interleukin 8 -251 A/T and +781 C/T polymorphisms and osteosarcoma risk in Chinese population: a case-control study. *Tumour biology : the journal of the International Society for Oncodevelopmental Biology and Medicine*, *37*(5), 6191–6196. https://doi.org/10.1007/S13277-015-4435-6

39. Cui, Y., Zhu, J.-J. jun, Ma, C.-B. bin, Cui, K., Wang, F., Ni, S.-H. hui, & Zhang, Z.-Y. yu. (2016). Interleukin 10 gene -1082A/G polymorphism is associated with osteosarcoma risk and poor outcomes in the Chinese population. *Tumour biology : the journal of the International Society for Oncodevelopmental Biology and Medicine*, *37*(4), 4517–4522. https://doi.org/10.1007/S13277-015-4238-9

40. Wang, J., Nong, L., Wei, Y., Qin, S., Zhou, Y., & Tang, Y. (2013). Association of interleukin-12 polymorphisms and serum IL-12p40 levels with osteosarcoma risk. *DNA and Cell Biology*, *32*(10), 605–610. https://doi.org/10.1089/dna.2013.2098

41. Tang, Y. J., Wang, J. L., Xie, K. G., & Lan, C. G. (2016). Association of interleukin 16 gene polymorphisms and plasma IL16 level with osteosarcoma risk. *Scientific Reports*, *6*. https://doi.org/10.1038/srep34607

42. Tang, Y.-J., Wang, J.-L., Nong, L.-G., Lan, C.-G., Zha, Z.-G., & Liao, P.-H. (2014). Associations of IL-27 polymorphisms and serum IL-27p28 levels with osteosarcoma risk. *Medicine (United States)*, *93*(10), e56. https://doi.org/http://dx.doi.org/10.1097/MD.0000000000000056

43. Yang, W., He, M., Zhao, J., & Wang, Z. (2014). Association of ITGA3 gene polymorphisms with susceptibility and clinicopathological characteristics of osteosarcoma. *Medical Oncology*, *31*(2), 826. https://doi.org/http://dx.doi.org/10.1007/s12032-013-0826-y

44. Liu, Y., Lv, B., He, Z., Zhou, Y., Han, C., Shi, G., … Yuan, W. (2012). Lysyl oxidase polymorphisms and susceptibility to osteosarcoma. *PloS one*, *7*(7), e41610. https://doi.org/10.1371/journal.pone.0041610

45. Bilbao-Aldaiturriaga, N., Askaiturrieta, Z., Granado-Tajada, I., Goričar, K., Dolžan, V., Group, F. T. S. O. S., … Garcia-Orad, A. (2016). A systematic review and meta-analysis of MDM2 polymorphisms in osteosarcoma susceptibility. *Pediatric Research*, *80*(4), 472–479. https://doi.org/http://dx.doi.org/10.1038/pr.2016.120

46. Naumov, V. A., Generozov, E. V., Solovyov, Y. N., Aliev, M. D., & Kushlinsky, N. E. (2012). Association of FGFR3 and MDM2 gene nucleotide polymorphisms with bone tumors. *Bulletin of Experimental Biology and Medicine*, *153*(6), 870–874. https://doi.org/10.1007/s10517-012-1847-9

47. Mirabello, L., Yu, K., Berndt, S. I., Burdett, L., Wang, Z., Chowdhury, S., … Savage, S. A. (2011). A comprehensive candidate gene approach identifies genetic variation associated with osteosarcoma. *BMC Cancer*, *11*. https://doi.org/10.1186/1471-2407-11-209

48. Toffoli, G., Biason, P., Russo, A., De Mattia, E., Cecchin, E., Hattinger, C. M., … Serra, M. (2009). Effect of TP53 Arg72Pro and MDM2 SNP309 polymorphisms on the risk of high-grade osteosarcoma development and survival. *Clinical Cancer Research*, *15*(10), 3550–3556. https://doi.org/10.1158/1078-0432.CCR-08-2249

49. Cui, Y., Zhu, J.-J., Ma, C.-B., Cui, K., Wang, F., Ni, S.-H., … S.-H, N. (2016). Genetic polymorphisms in MMP 2, 3 and 9 genes and the susceptibility of osteosarcoma in a Chinese Han population. *Biomarkers : biochemical indicators of exposure, response, and susceptibility to chemicals*, *21*(2), 160–163. https://doi.org/10.3109/1354750X.2015.1118550

50. Zhi, L., Liu, D., Wu, S. ., Li, T., Zhao, G., & Zhao, B. (2016). Association of common variants in MTAP with susceptibility and overall survival of osteosarcoma: A two-stage population-based study in Han Chinese. *Journal of Cancer*, *7*(15), 2179–2186. https://doi.org/http://dx.doi.org/10.7150/jca.16609

51. Huang, Z., Yuan, L., & Jiang, Z. (2015). Associations of polymorphisms in NAT2 gene with risk and metastasis of osteosarcoma in young Chinese population. *OncoTargets and Therapy*, *8*, 2675–2680. https://doi.org/http://dx.doi.org/10.2147/OTT.S92275

52. Goričar, K., Kovač, V., Jazbec, J., Lamovec, J., Dolžan, V., K., G., … J., L. (2015). Homologous recombination repair polymorphisms and the risk for osteosarcoma. *Journal of Medical Biochemistry*, *34*(2), 200–206. https://doi.org/http://dx.doi.org/10.2478/jomb-2014-0031

53. Zhang, Y., Hu, X., Wang, H.-K., Shen, W.-W., Liao, T.-Q., Chen, P., & Chu, T.-W. (2014). Single-nucleotide polymorphisms of the PRKCG gene and osteosarcoma susceptibility. *Tumor Biology*, *35*(12), 12671–12677. https://doi.org/http://dx.doi.org/10.1007/s13277-014-2591-8

54. Lu, H., Zhu, L., Lian, L., Chen, M., Shi, D., Wang, K., … D., S. (2015). Genetic variations in the PRKCG gene and osteosarcoma risk in a Chinese population: a case-control study. *Tumour biology : the journal of the International Society for Oncodevelopmental Biology and Medicine*, *36*(7), 5241–5247. https://doi.org/10.1007/s13277-015-3182-z

55. Zhi, L.-Q., Ma, W., Zhang, H., Zeng, S.-X., Chen, B., L.-Q., Z., … S.-X., Z. (2014). Association of RECQL5 gene polymorphisms and osteosarcoma in a Chinese Han population. *Tumour biology : the journal of the International Society for Oncodevelopmental Biology and Medicine*, *35*(4), 3255–3259. https://doi.org/http://dx.doi.org/10.1007/s13277-013-1425-4

56. Dong, Y. Z., Huang, Y. X., & Lu, T. (2015). Single nucleotide polymorphism in the RECQl5 gene increased osteosarcoma susceptibility in a Chinese han population. *Genetics and Molecular Research*, *14*(1), 1899–1902. https://doi.org/http://dx.doi.org/10.4238/2015.March.13.18

57. Wu, Y., Zhao, J., & He, M. (2015). Correlation between TGF-β1 gene 29 T > C single nucleotide polymorphism and clinicopathological characteristics of osteosarcoma. *Tumour biology : the journal of the International Society for Oncodevelopmental Biology and Medicine*, *36*(7), 5149–5156. https://doi.org/10.1007/s13277-015-3168-x

58. Xu, S., Yang, S., Sun, G., Huang, W., & Zhang, Y. (2014). Transforming Growth Factor-Beta Polymorphisms and Serum Level in the Development of Osteosarcoma. *DNA and Cell Biology*, *33*(11), 802–806. https://doi.org/10.1089/dna.2014.2527

59. Zhao, Z., Tang, X., Song, K., Li, X., & Zhang, Y. (2015). Association of -308G/A and -238G/A polymorphisms of TNF-α and osteosarcoma risk. *International Journal of Clinical and Experimental Pathology*, *8*(4), 4177. Retrieved from /pmc/articles/PMC4466997/

60. Patio-Garcia, A., Sotillo-Pieiro, E., Modesto, C., Sierrases-Maga, L., A., P.-G., E., S.-P., … Sierrasesumaga, L. (2000). Analysis of the human tumour necrosis factor-alpha (TNFα) gene promoter polymorphisms in children with bone cancer [3]. *Journal of Medical Genetics*, *37*(10), 789–791. https://doi.org/10.1136/jmg.37.10.789

61. Savage, S. A., Burdett, L., Troisi, R., Douglass, C., Hoover, R. N., & Chanock, S. J. (2007). Germ-line genetic variation of TP53 in osteosarcoma. *Pediatric Blood and Cancer*, *49*(1), 28–33. https://doi.org/10.1002/pbc.21077

62. Ru, J.-Y., Cong, Y., Kang, W.-B., Yu, L., Guo, T., Zhao, J.-N., … T., G. (2015). Polymorphisms in TP53 are associated with risk and survival of osteosarcoma in a Chinese population. *International journal of clinical and experimental pathology*, *8*(3), 3198–3203. Retrieved from http://ovidsp.ovid.com/ovidweb.cgi?T=JS&PAGE=reference&D=emed16&NEWS=N&AN=615679648

63. Zhang, J., Kai, L., Zhang, W., & Yin, Y. (2018). Association between genetic variants in p53 binding sites and risks of osteosarcoma in a Chinese population: a two-stage case-control study. *Cancer Biology and Therapy*, *19*(11), 994–997. https://doi.org/http://dx.doi.org/10.1080/15384047.2018.1456607

64. Zhuang, Y. S., Han, G. Q., Zhang, H. ., & Yan, J. . (2015). Association between angiogenic growth factor genetic polymorphisms and the risk of osteosarcoma. *Genetics and Molecular Research*, *14*(3), 10524–10529. https://doi.org/http://dx.doi.org/10.4238/2015.September.8.14

65. Zhao, L. L., Lin, W., & Lei, S. (2015). Investigation on the role of VEGF gene polymorphisms in the risk of osteosarcoma. *Pakistan Journal of Medical Sciences*, *31*(2), 364–368. https://doi.org/http://dx.doi.org/10.12669/pjms.312.6533

66. Tie, Z., Bai, R., Zhai, Z., Zhang, G., Zhang, H., Zhao, Z., … D., Z. (2014). Single nucleotide polymorphisms in VEGF gene are associated with an increased risk of osteosarcoma. *International journal of clinical and experimental pathology*, *7*(11), 8143–8149. Retrieved from http://ovidsp.ovid.com/ovidweb.cgi?T=JS&PAGE=reference&D=emed15&NEWS=N&AN=609753017

67. Liu, J.-Q., Bai, X., Duan, D.-C., & Duo, A.-X. (2015). Role of five small nucleotide polymorphisms in the VEGF gene on the susceptibility to osteosarcoma and overall survival of patients. *Oncology Letters*, *10*(3), 1481–1486. https://doi.org/10.3892/ol.2015.3396

68. Wang, Z., Wen, P., Luo, X., Fang, X., Wang, Q., Ma, F., & Lv, J. (2014). Association of the vascular endothelial growth factor (VEGF) gene single-nucleotide polymorphisms with osteosarcoma susceptibility in a Chinese population. *Tumor Biology*, *35*(4), 3605–3610. https://doi.org/http://dx.doi.org/10.1007/s13277-013-1475-7

69. Zhang, G., Bai, R., Zhang, T., Zhang, H., Wen, S. Z., Jiang, D. M., … S.Z., W. (2015). Investigation of the role of VEGF gene polymorphisms in the risk of osteosarcoma. *Genetics and Molecular Research*, *14*(3), 8283–8289. https://doi.org/http://dx.doi.org/10.4238/2015.July.27.16

70. Li, J., Zhou, T., Lin, H., Chen, X., Wang, S., Yin, Z., … S., W. (2017). Vascular endothelial growth factor (VEGF) rs3025039 polymorphism is associated with increased risk of osteosarcoma. *Biomedical Research (India)*, *28*(14), 6300–6302. Retrieved from http://www.biomedres.info/biomedical-research/vascular-endothelial-growth-factor-vegf-rs3025039-polymorphism-is-associated-with-increased-risk-of-osteosarcoma.pdf

71. Cao Lei, Zhang Shailin, M. W. (2016). The vascular endothelial growth factor (VEGF) gene rs2010963 and rs3025039 polymorphisms and risk of osteosarcoma in Chinese population: evidence from a case-control study and a meta-analysis. *Int J Clin Exp Pathol*, *9*(11), 11276–11288.

72. Hu, G. L., Ma, G., Ming, J. H., G.L., H., & G., M. (2015). Impact of common SNPs in VEGF gene on the susceptibility of osteosarcoma. *Genetics and Molecular Research*, *14*(4), 14561–14566. https://doi.org/http://dx.doi.org/10.4238/2015.November.18.19

73. Guo, J., Lv, H. C., Shi, R. H., Liu, W. L., J., G., H.C., L., & R.H., S. (2015). Association between XRCC3 Thr241Met polymorphism and risk of osteosarcoma in a Chinese population. *Genetics and Molecular Research*, *14*(4), 16484–16490. https://doi.org/http://dx.doi.org/10.4238/2015.December.9.20

74. Yang, L., An, Y., Wang, G., Lu, T., & Yang, S. (2015). Association between XRCC3 Thr241Met polymorphism and risk of osteosarcoma in a Chinese population. *International journal of clinical and experimental pathology*, *8*(9), 11670–11674. Retrieved from http://ovidsp.ovid.com/ovidweb.cgi?T=JS&PAGE=reference&D=emed16&NEWS=N&AN=615936127

75. Koshkina, N. V, Kleinerman, E. S., Li, G., Zhao, C. C., Wei, Q., & Sturgis, E. M. (2007). Exploratory analysis of Fas gene polymorphisms in pediatric osteosarcoma patients. *Journal of Pediatric Hematology/Oncology*, *29*(12), 815–821. https://doi.org/http://dx.doi.org/10.1097/MPH.0b013e3181581506

**Supplementary Figures**

*Supplementary Figure 1:*

*Graphical display of random-effects meta-analyses results*

### VEGF rs699947

**1)**


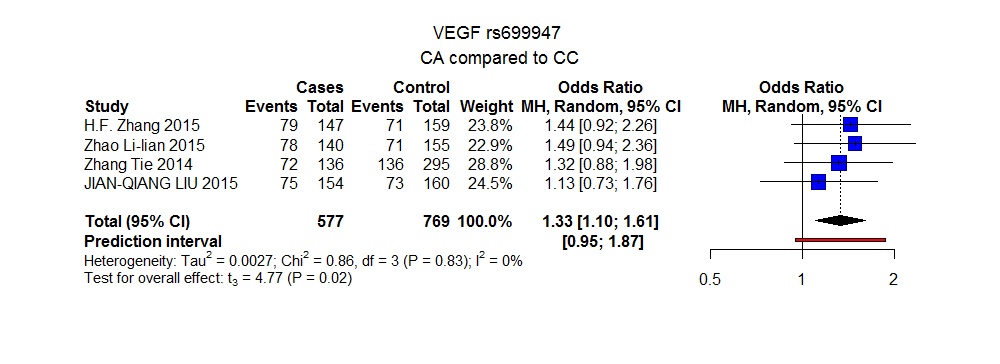


**2)**


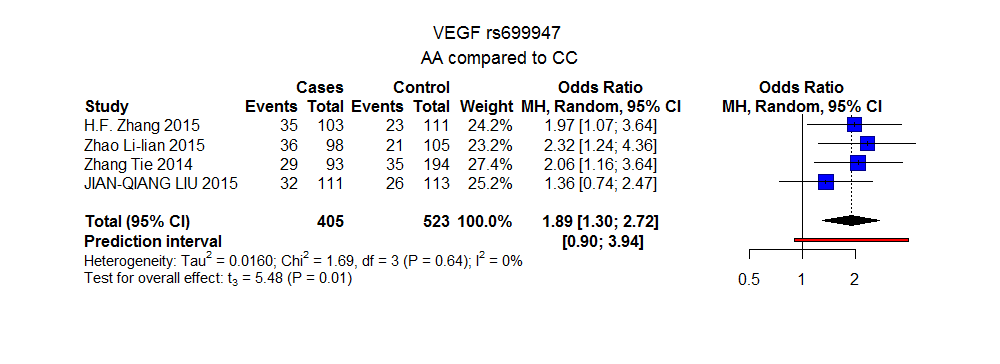


**3)**


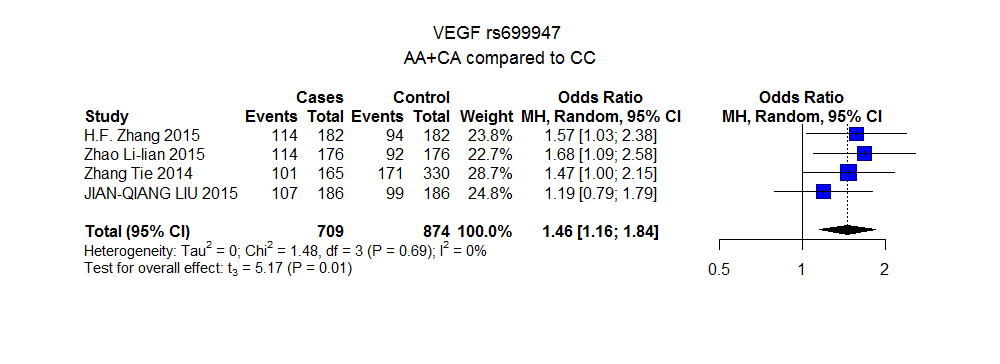


**4)**


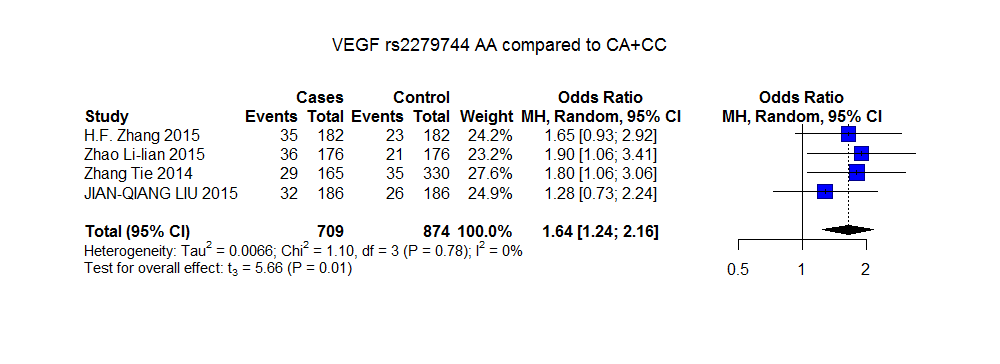


**5)**


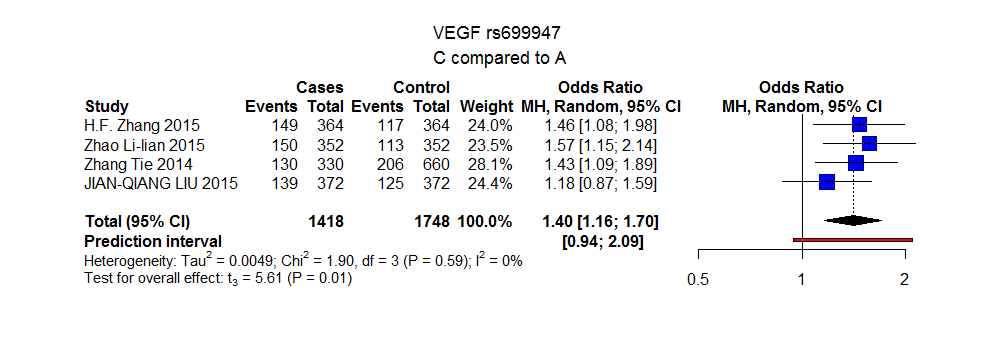


### VEGF rs3025039

**1)**

**
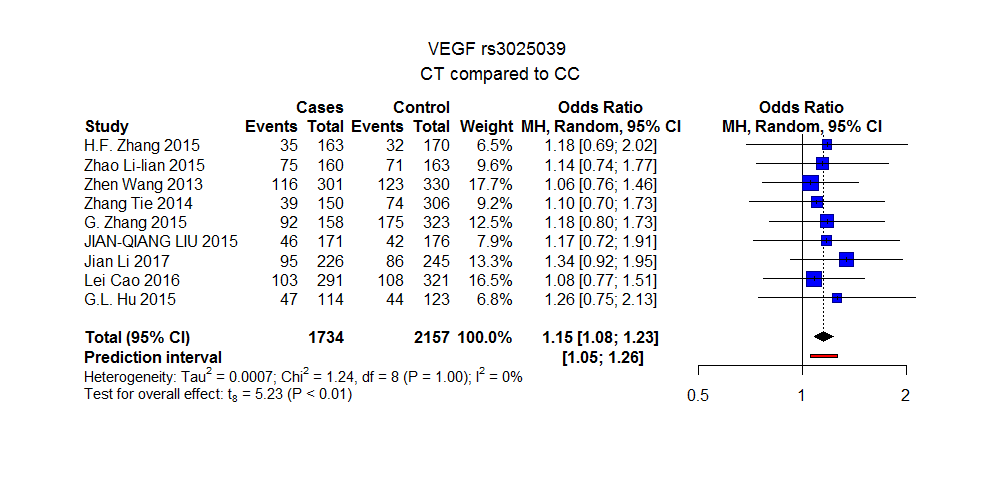
**

**2)**


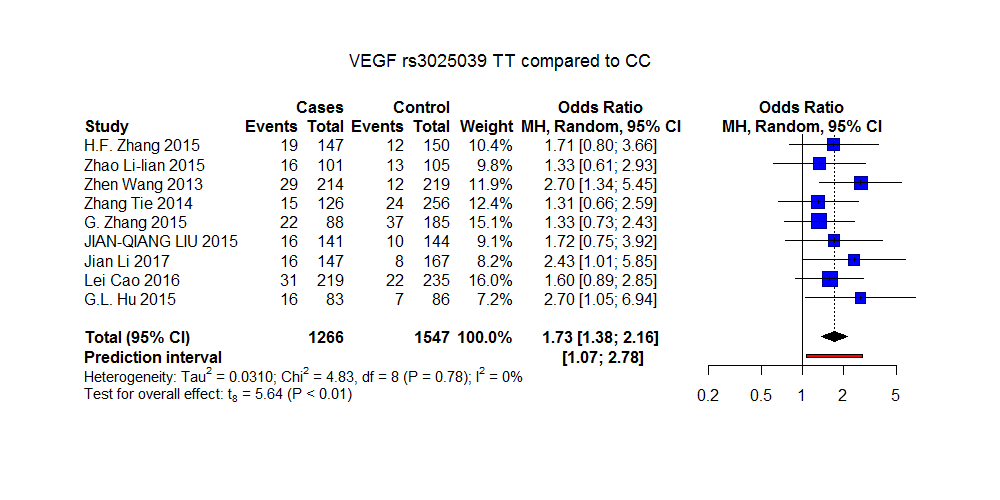


**3)**

**
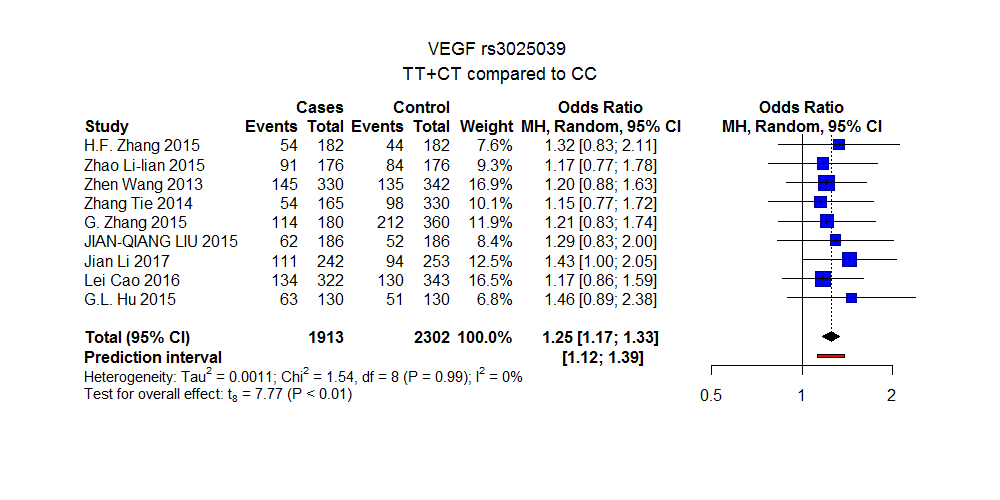
**

**4)** **
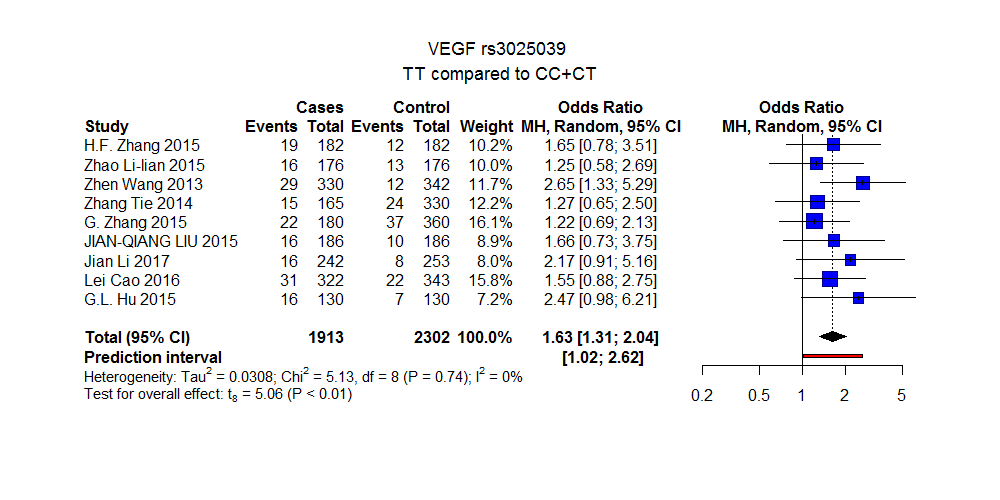
**

**5)**

**
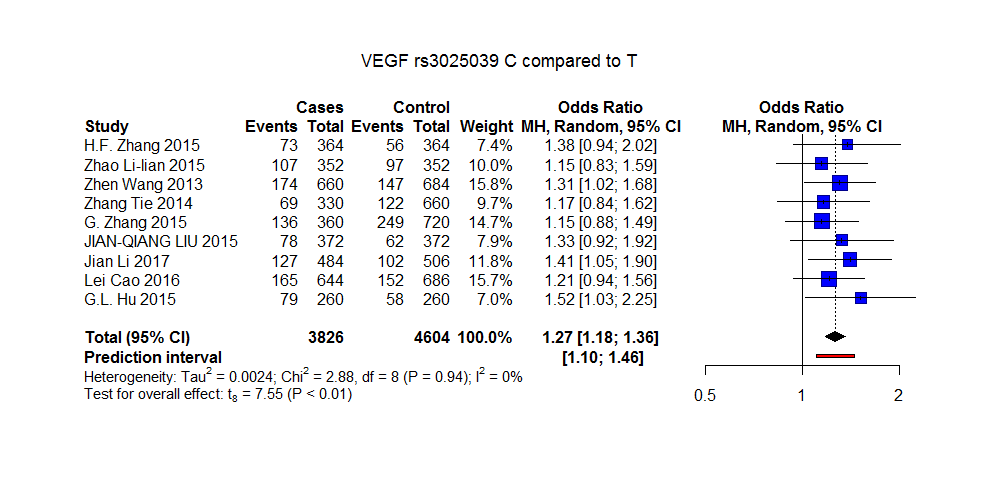
**

## VEGF rs1570360

1)


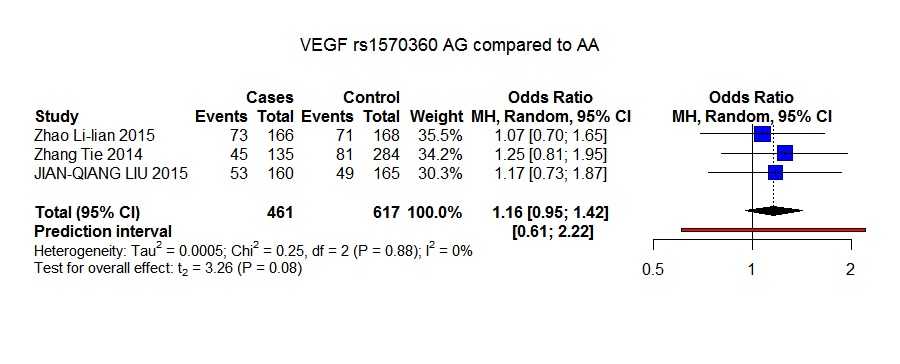


2)


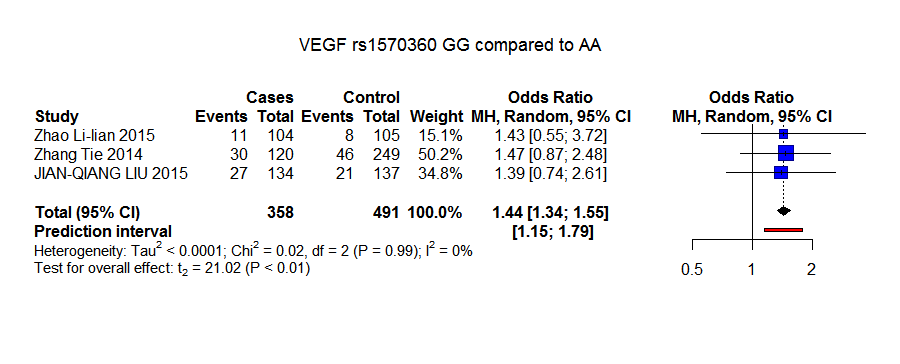


3)


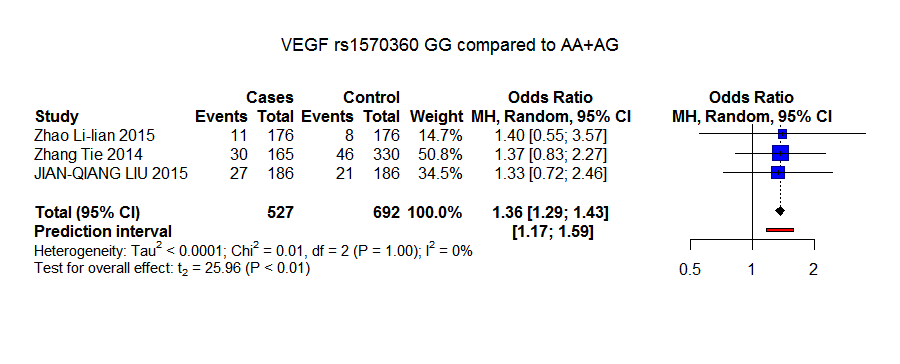


4)
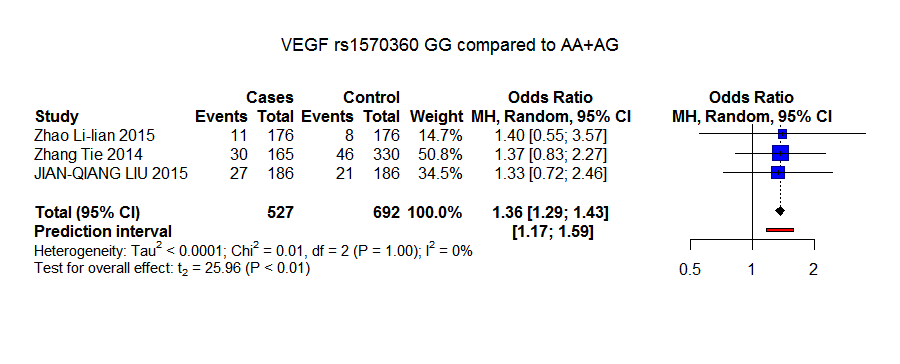


5)


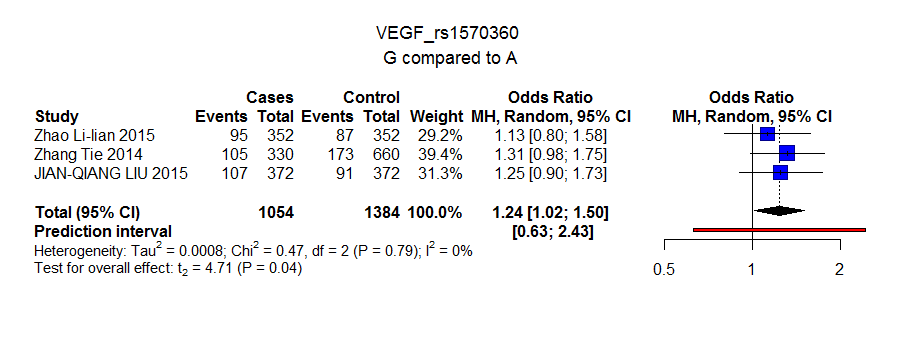


## VEGF rs2010963

1)


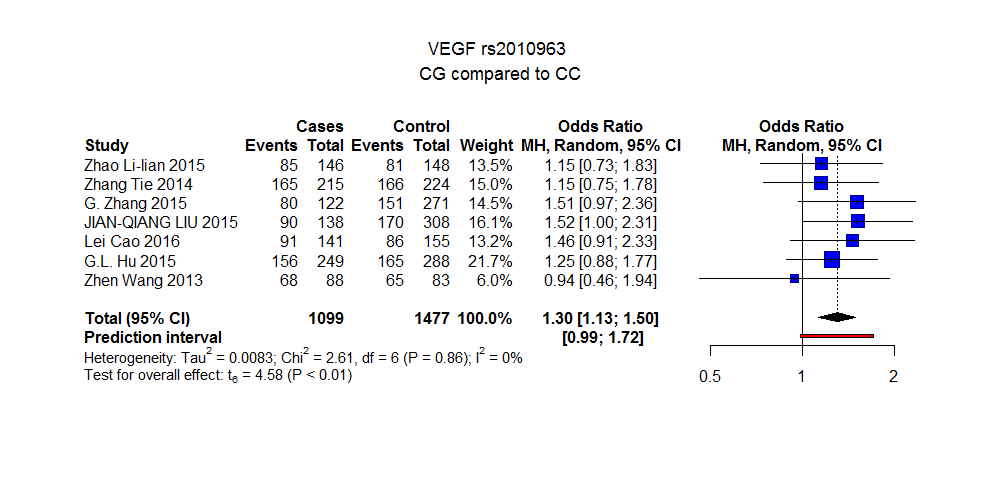


2)


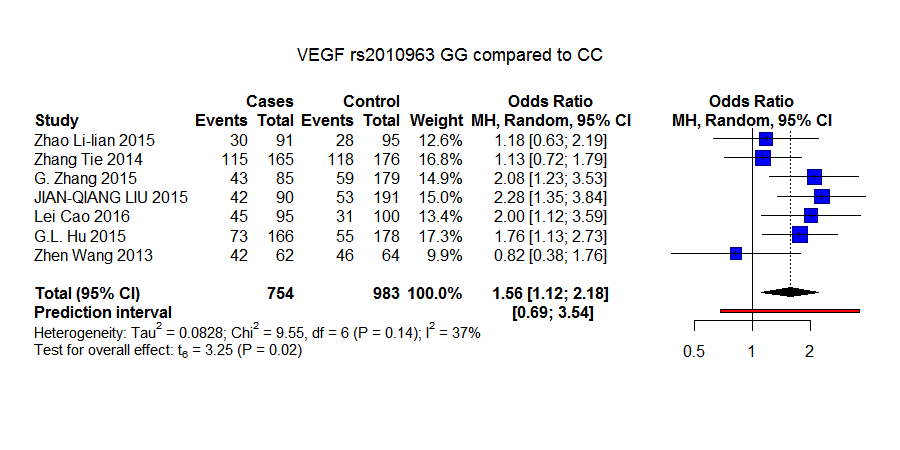


3)


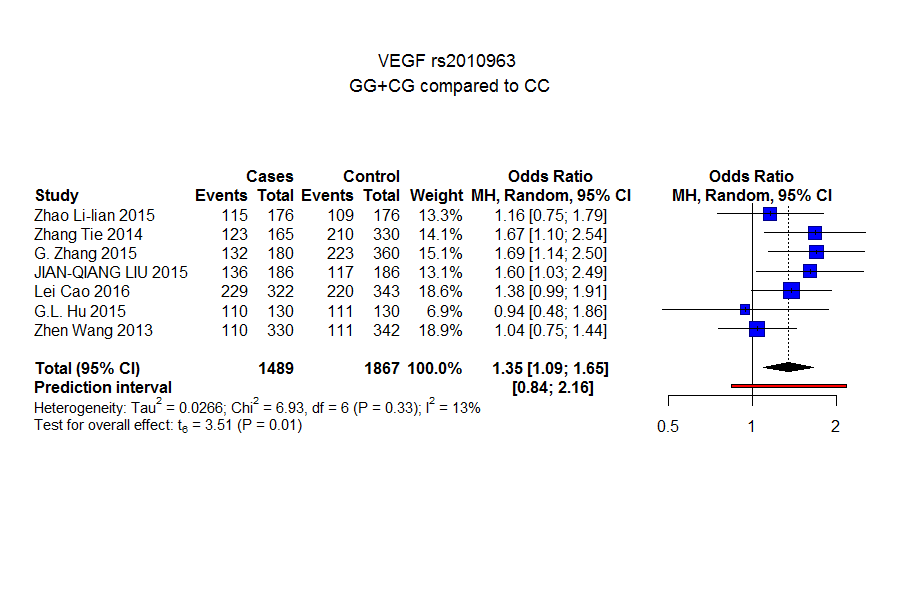


4)


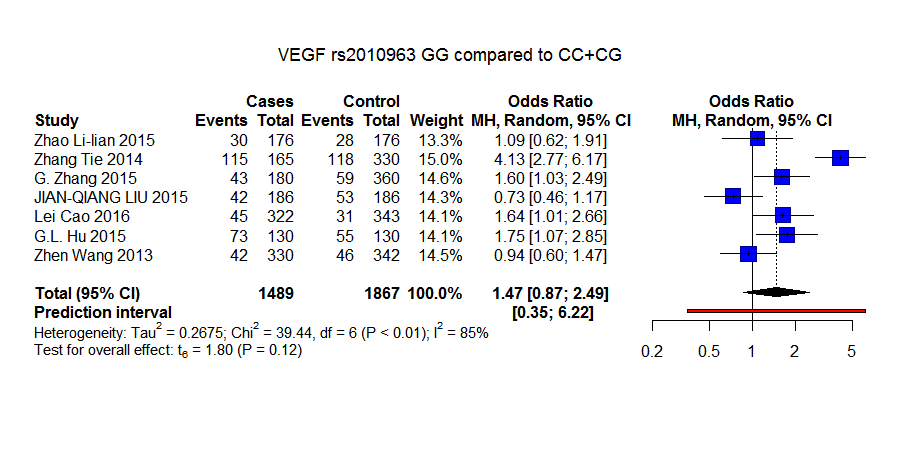


5)


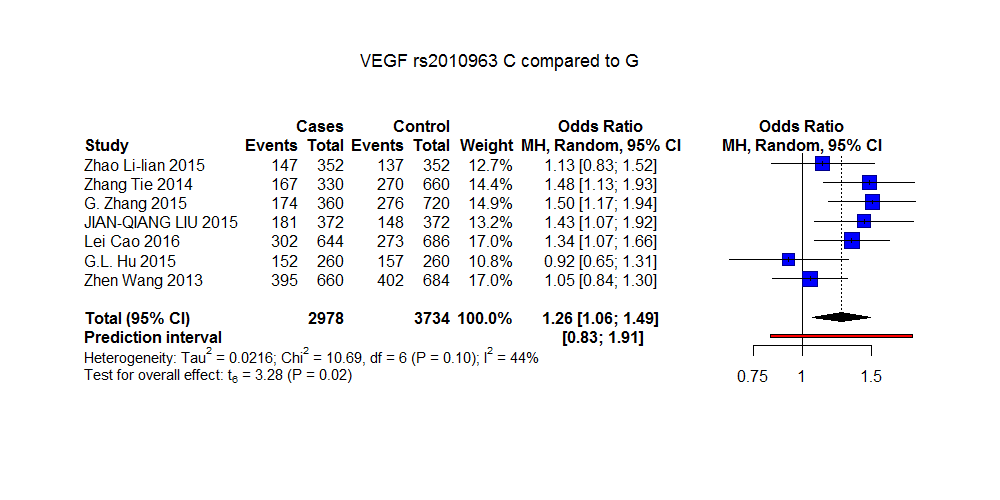


## VEGF rs10434

1)


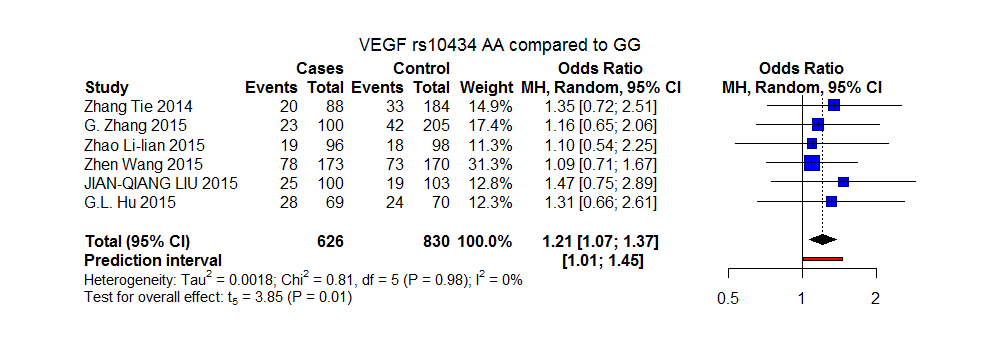


2)


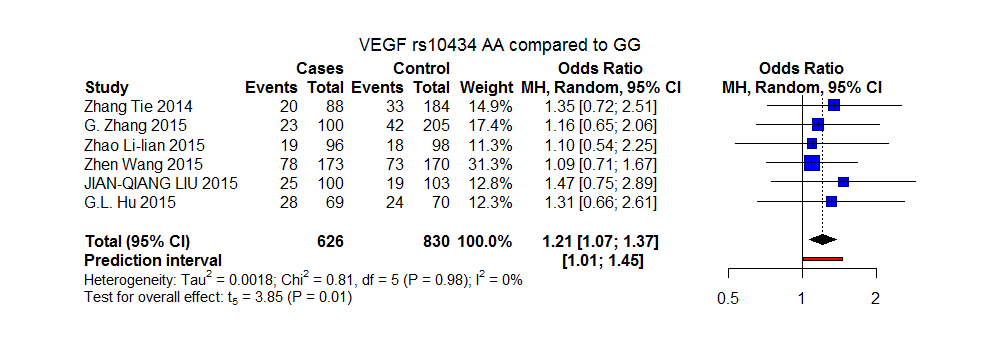


3)


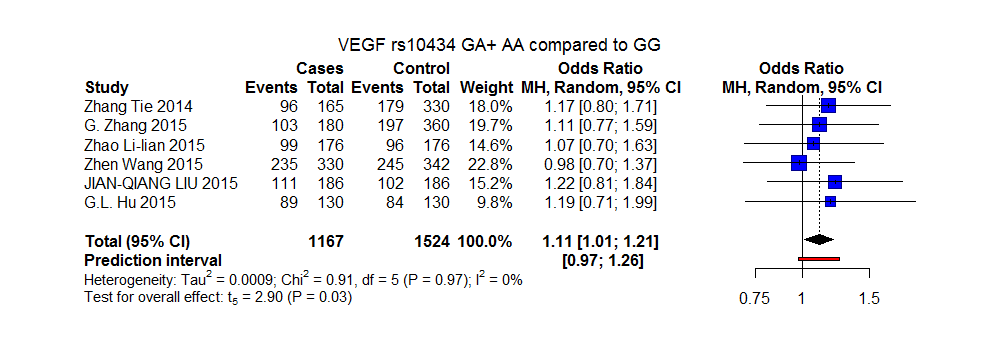


4)


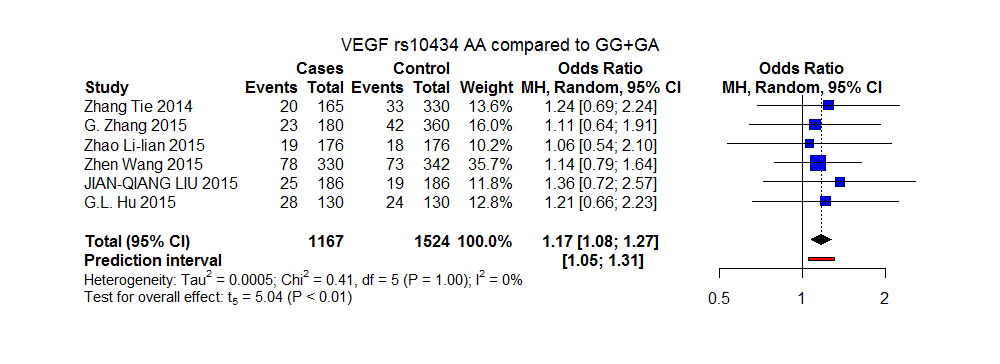


5)


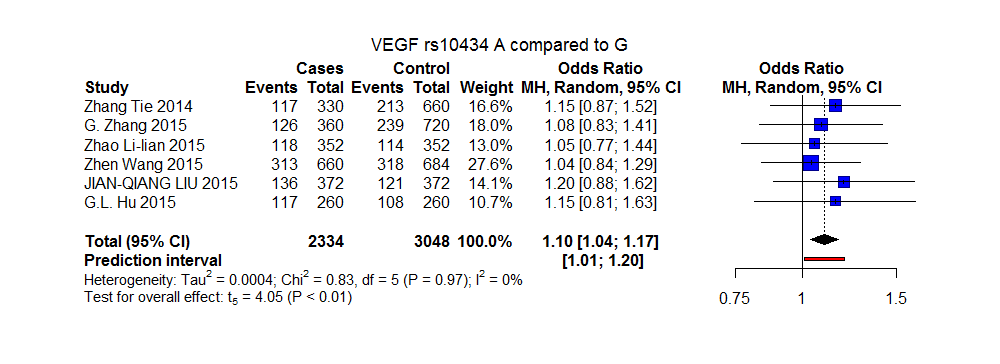


## IL-8 rs4073

1)


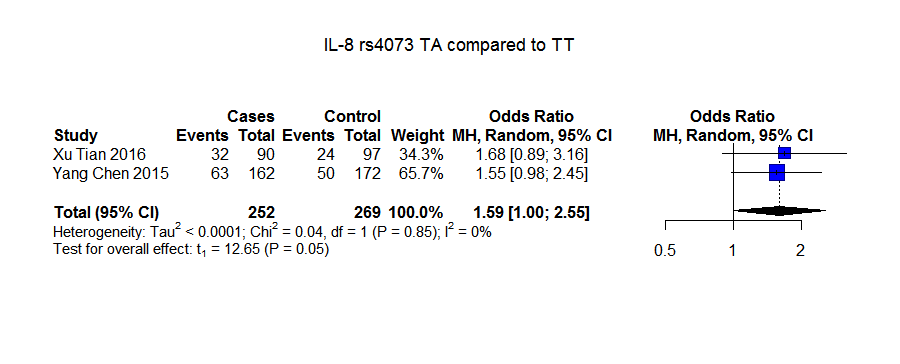


2)


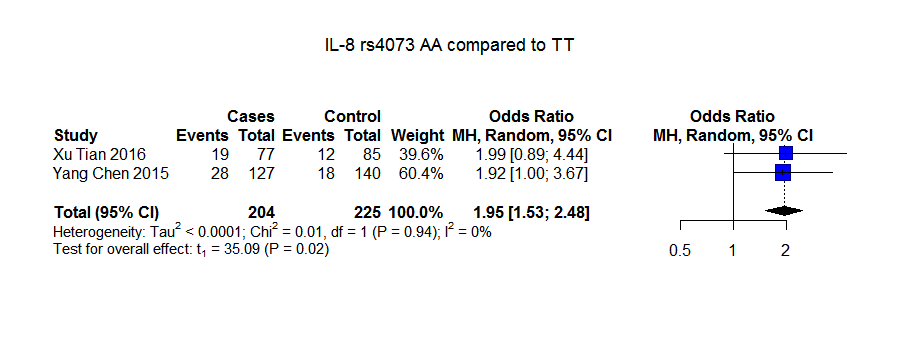


3)


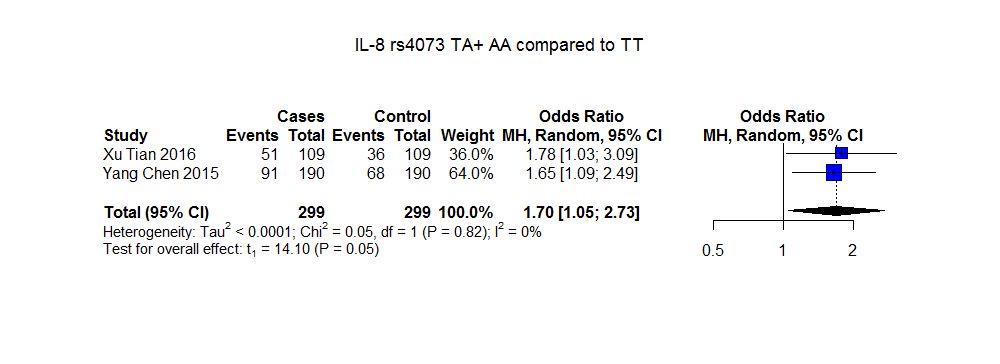


4)


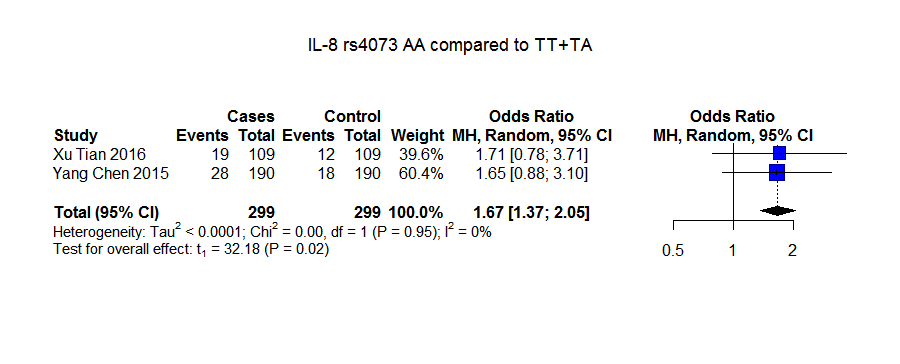


5)


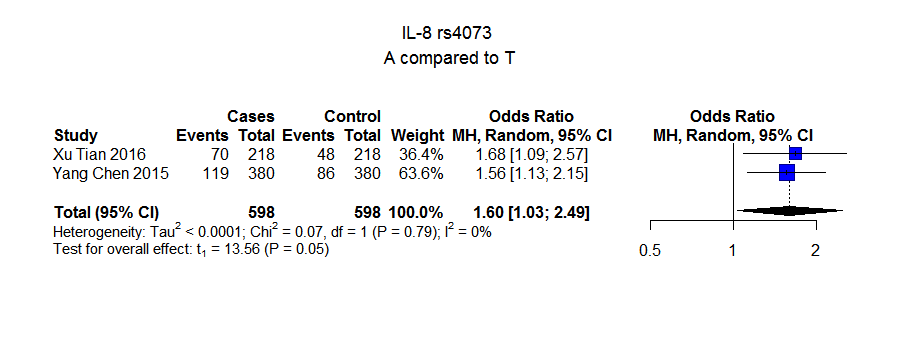


### CTLA-4 rs5742909

1)


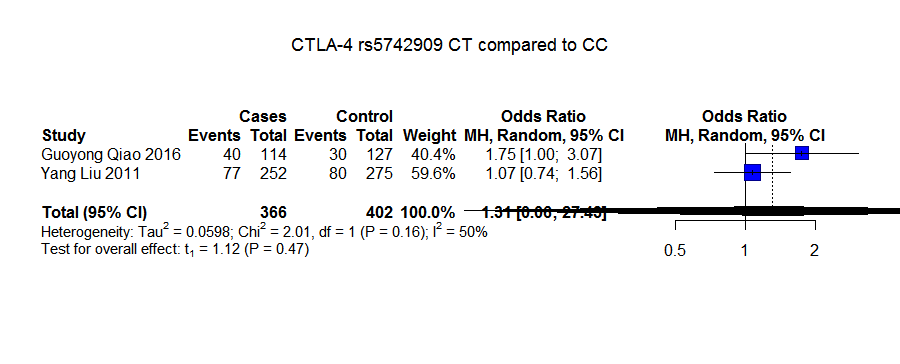


2)


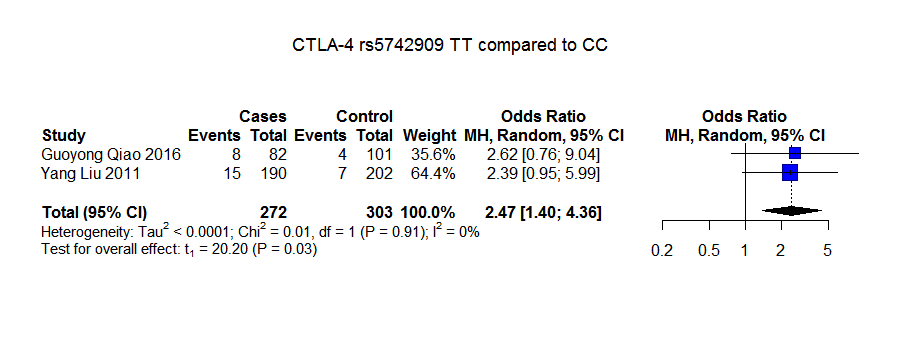


3)
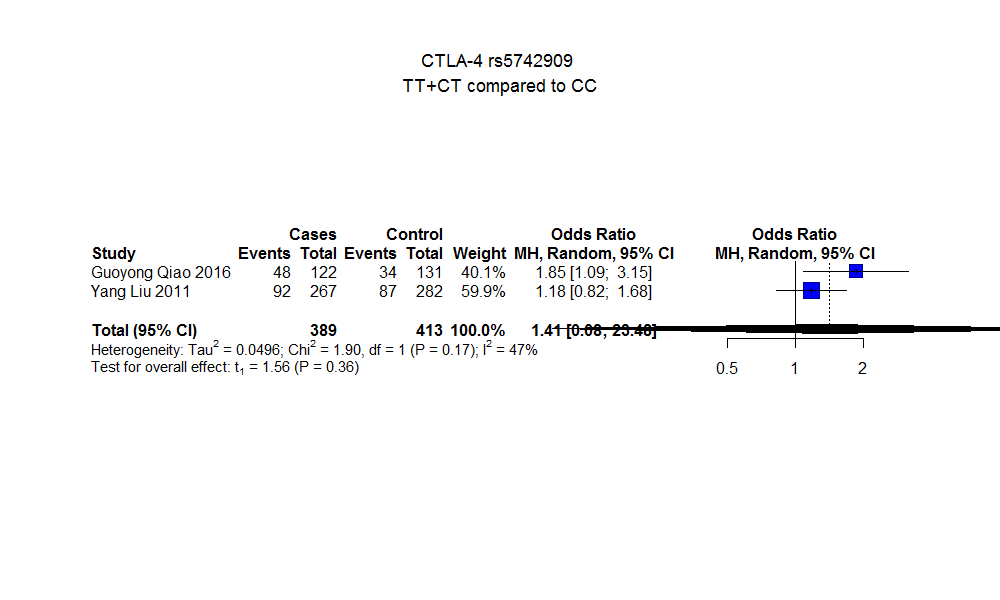


4)


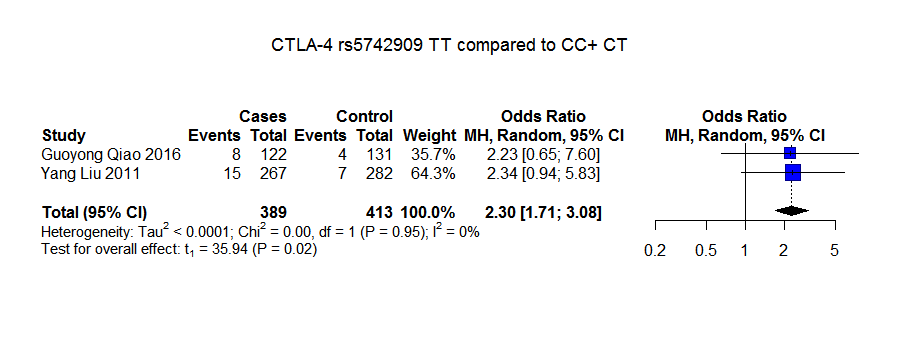


5)


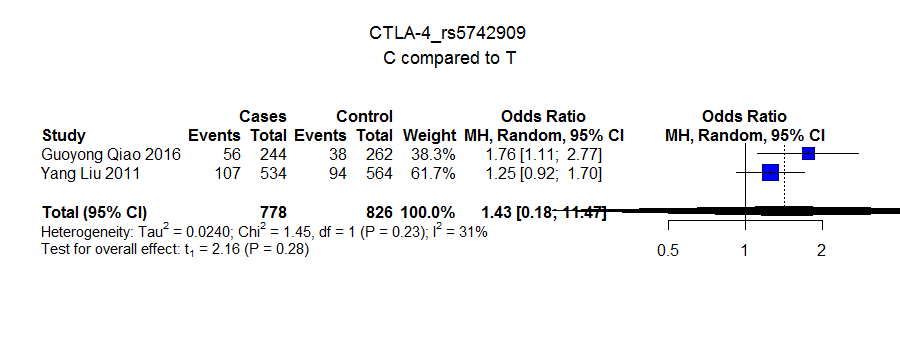


### XRCC3 rs861539


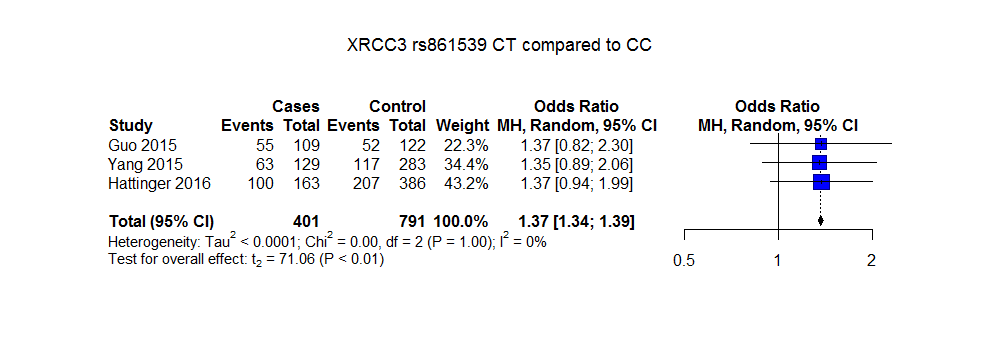


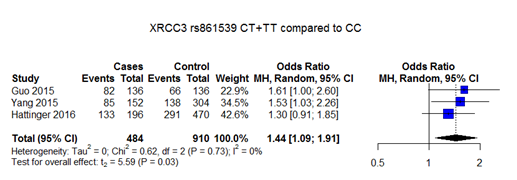


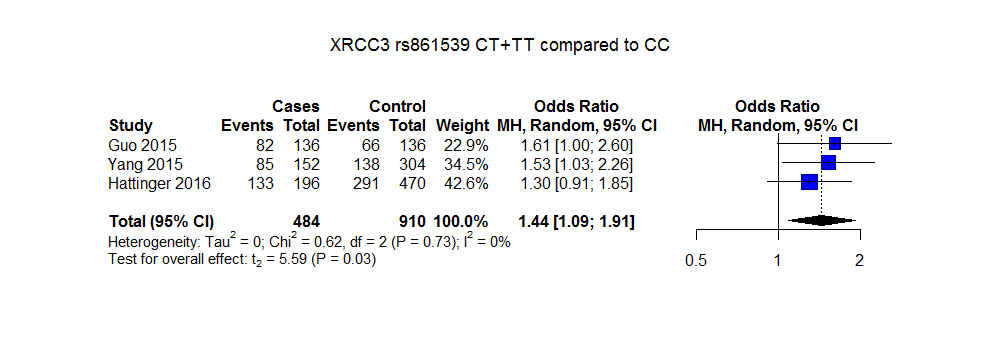


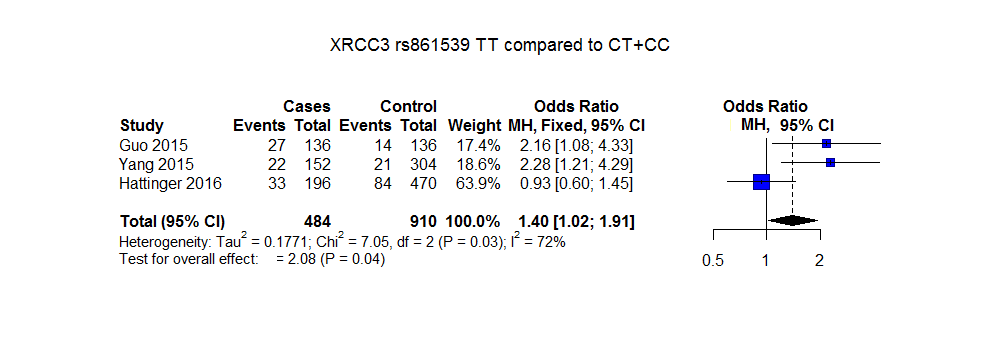


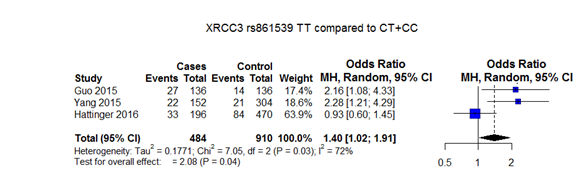


MDM2

### MDM2 rs1690916


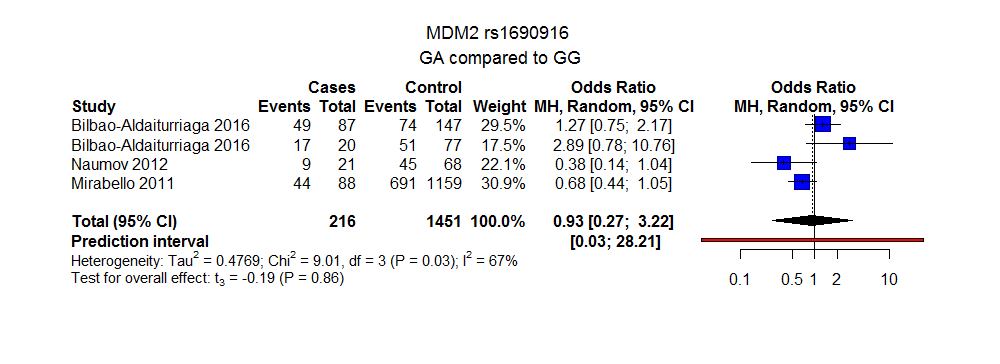


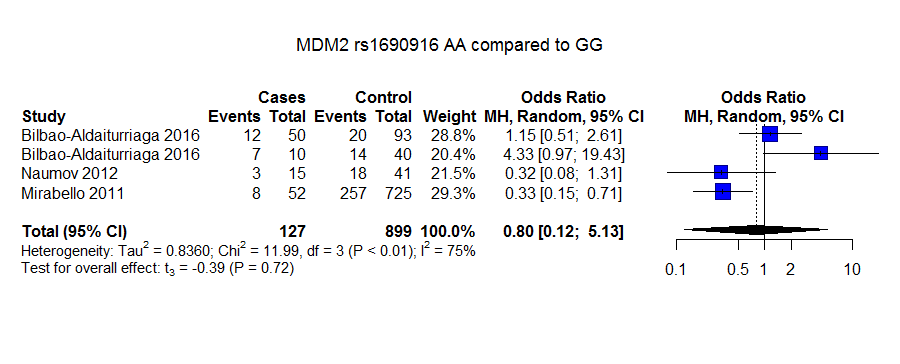


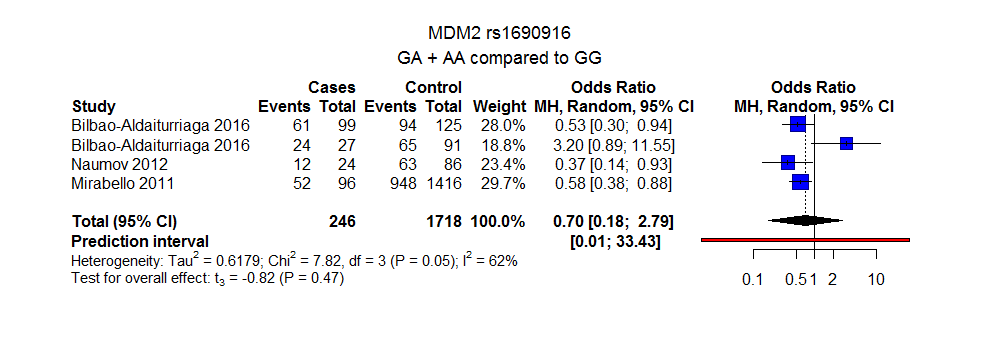


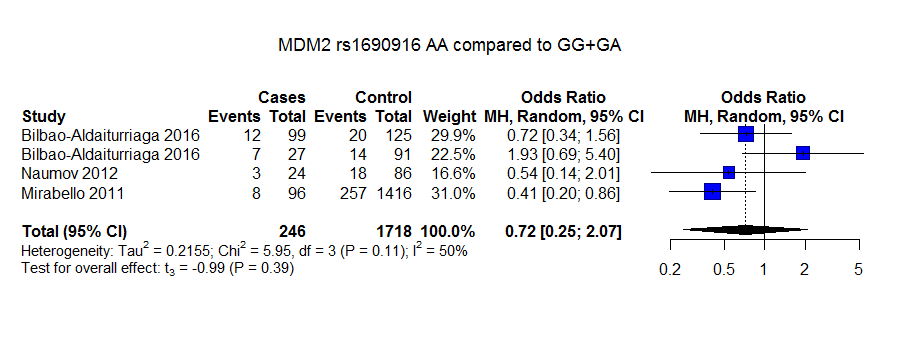


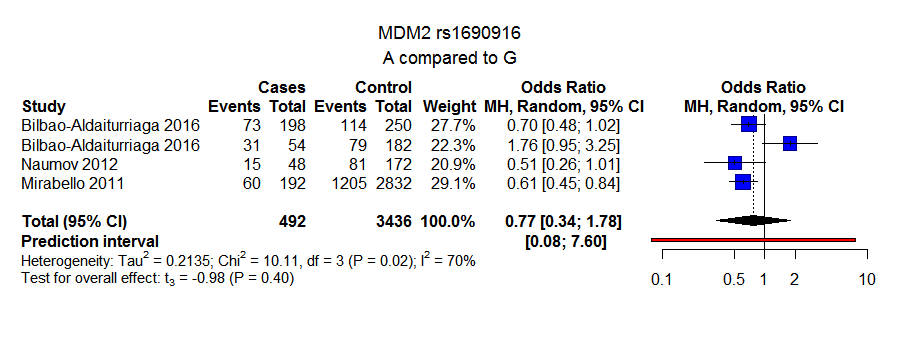


### MDM2 rs2279744


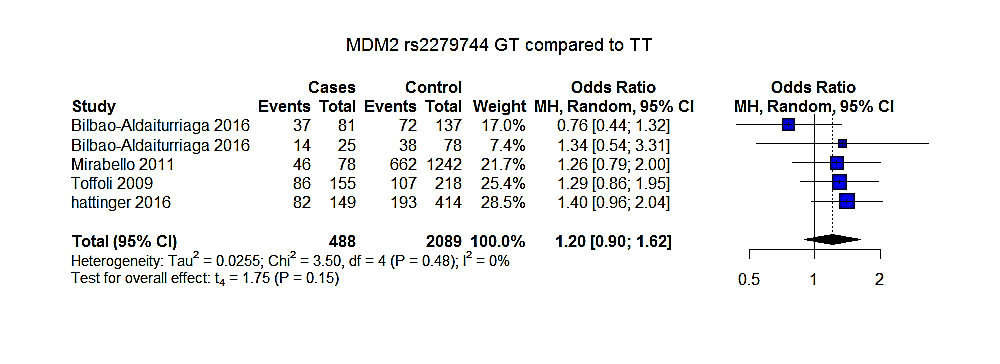


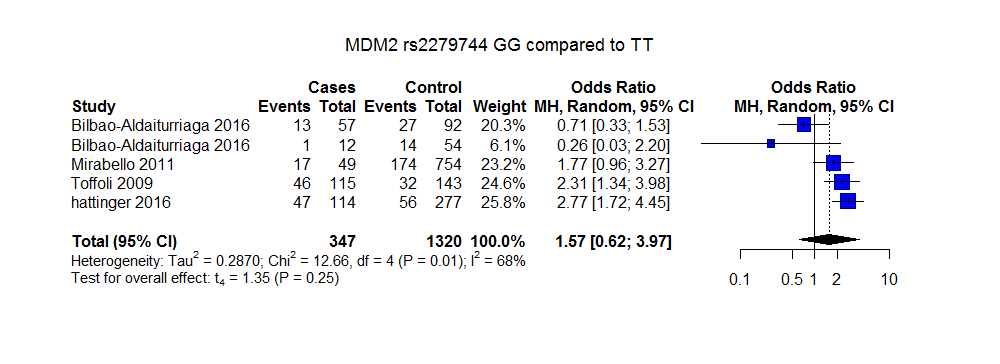


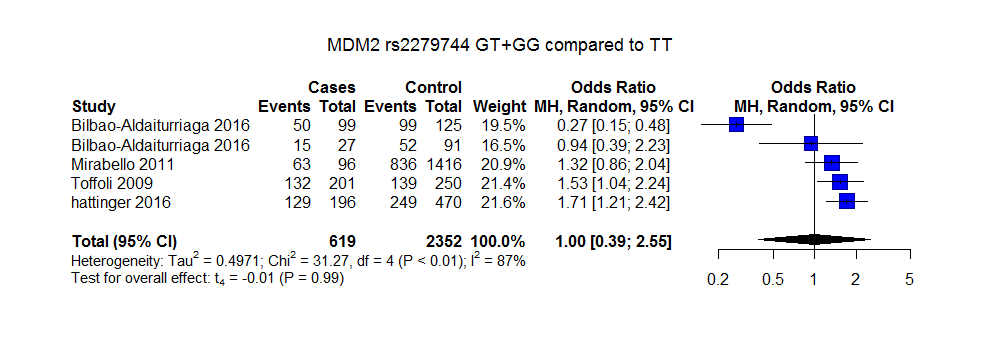


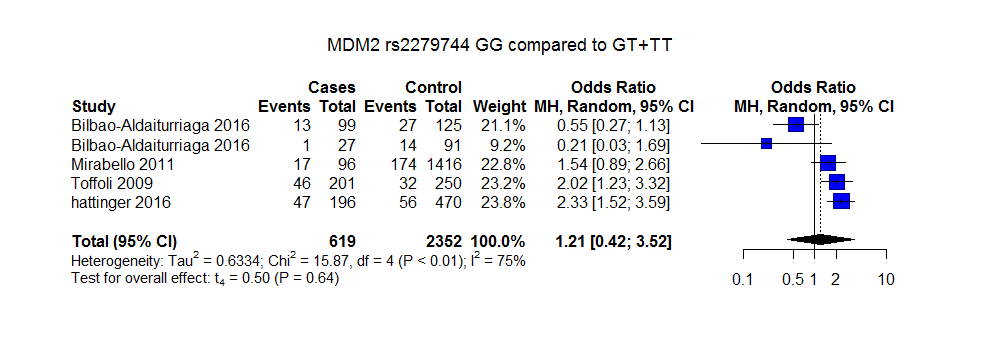


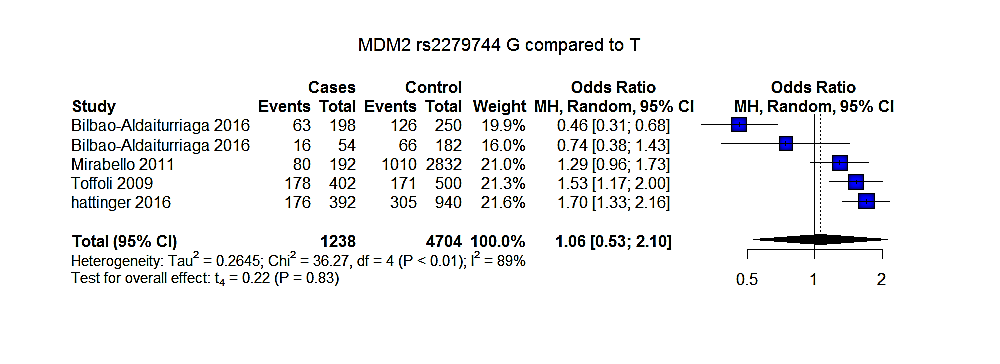


PRKCG rs454006


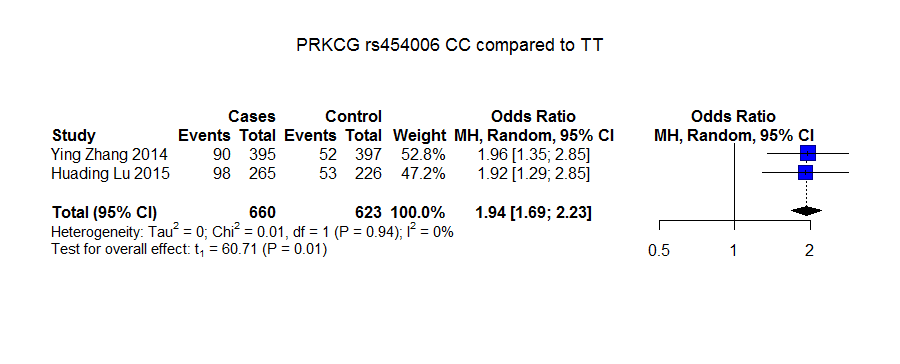


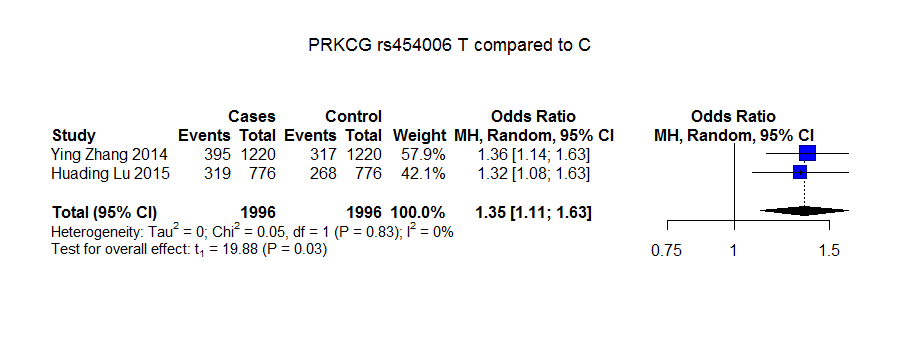


## RECQL5 rs820196


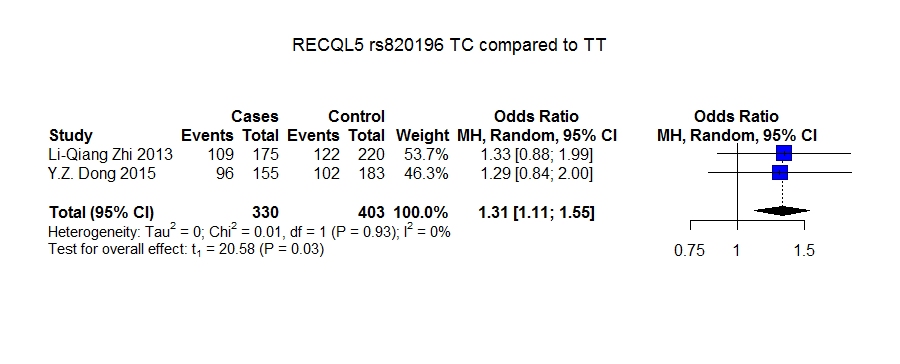


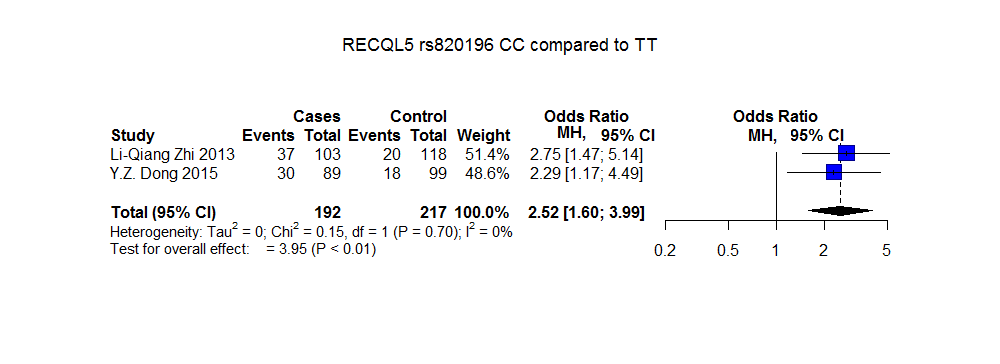


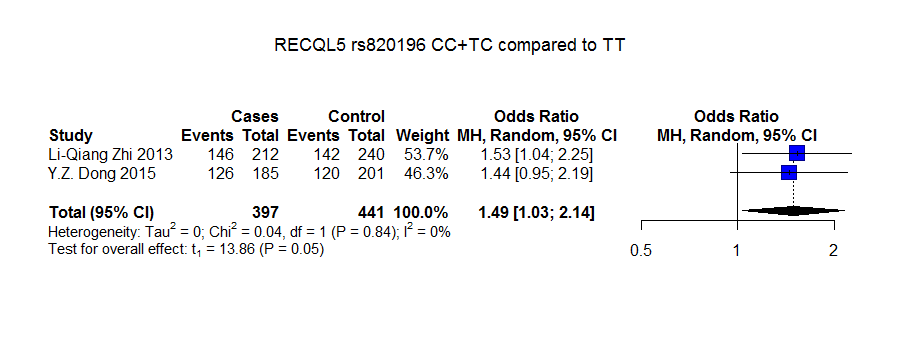


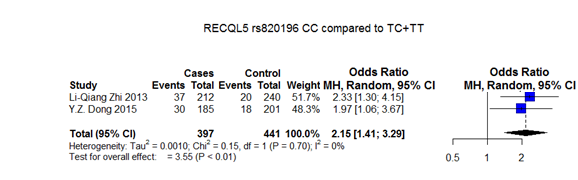


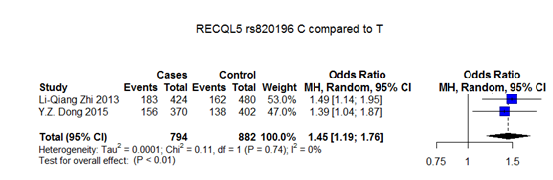


## TNF-α r*s*361525


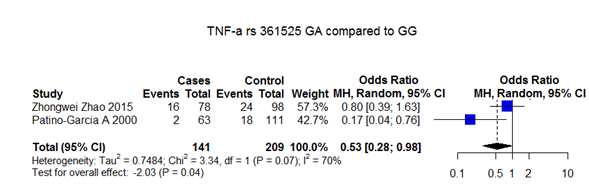


## ERCC3 rs4150506


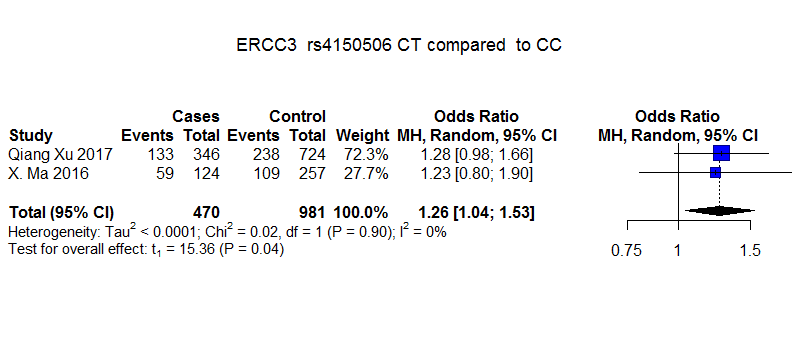


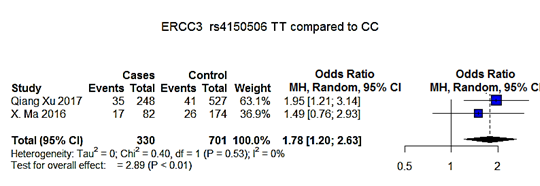


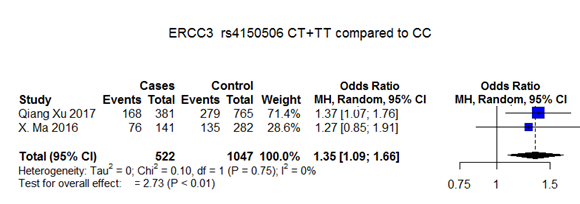


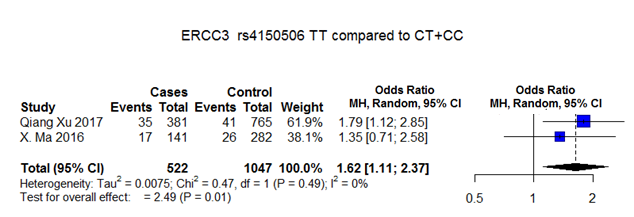


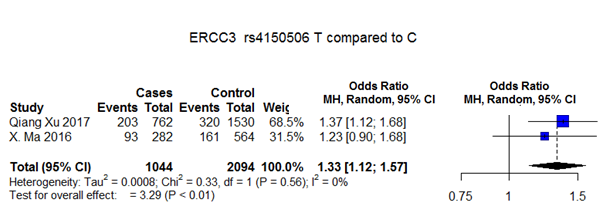


**Supplementary Figure S2**

# Sensitivity Analyses.

## VEGF rs699947

## leave-one-out- Analysis

### CA vs CC


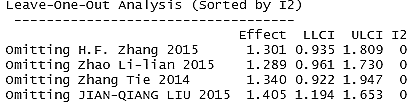


### AA vs CC


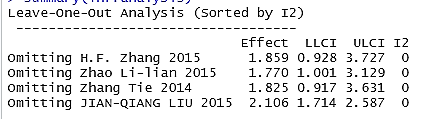


### AA +AC vs CC


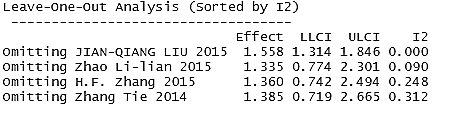


### AA vs AC+CC


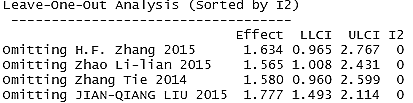


### A vs C


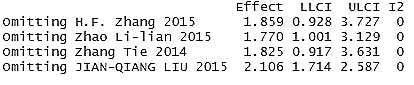


## rs1570360

## Leave-one-out Analysis

### AG vs AA


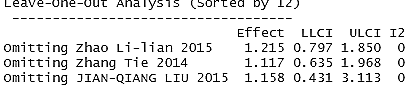


### GG vs AA


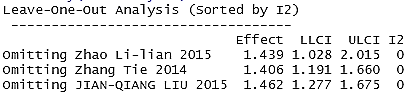


### GA+GG vs AA


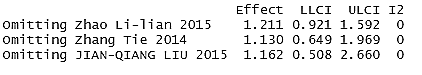


### GG vs AA+GA


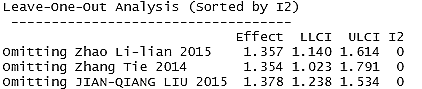


### G vs A


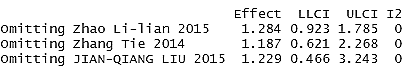


## VEGF rs10434

## leave-one-out- Analysis


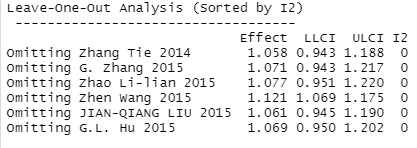


AA vs GG


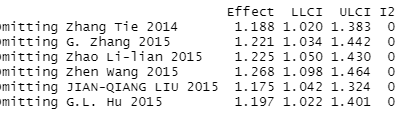


GA+AA vs GG


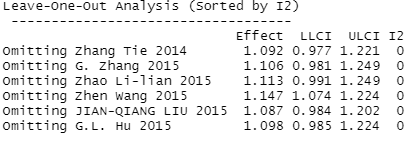


AA s GA+GG


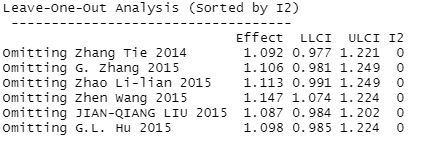


A vs G


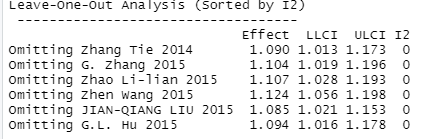


## XRCC3 rs861539

### CT vs CC


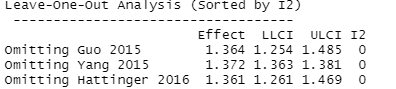


### TT vs CC


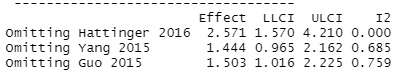


### CT+TT vs CC


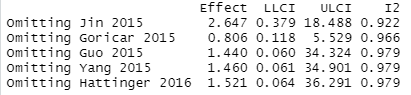


### TT vs CC+CT

###
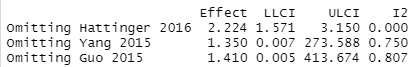


### T vs C


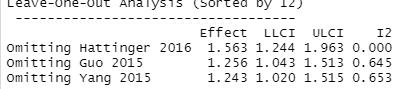


## CTLA-4 rs231775

### GA vs GG


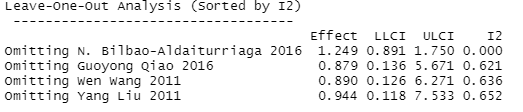


### AA vs GG


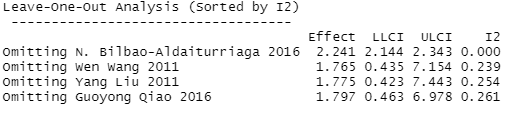


### GA +AA vs GG


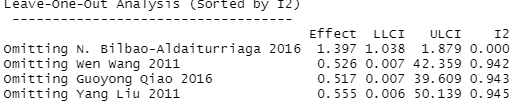


### AA vs GG+GA


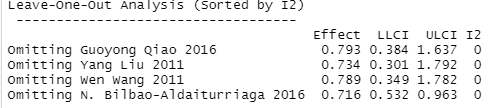


### G vs A


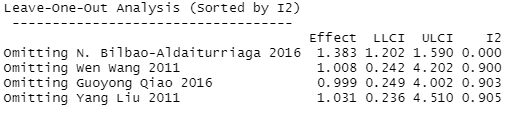


## MDM2 rs1690916

### GA +AA vs GG


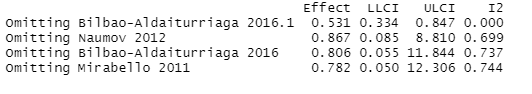


### A vs G


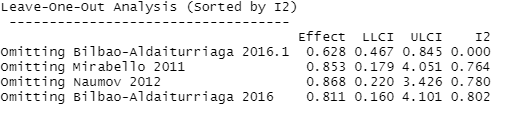


## MDM2 rs2279744

### GT vs TT


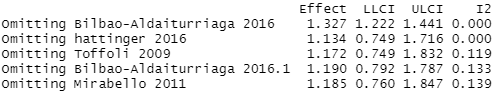


### GG vs TT


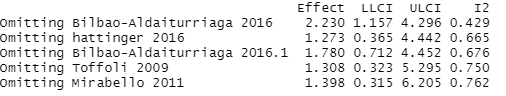


### GT+GG vs TT


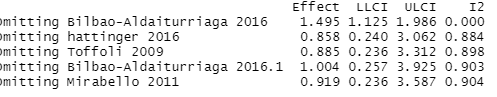

Supplement: Supplementary file 1 — Supplementary Information. [file 41598_2024_53802_MOESM1_ESM.docx]
